# Modeling the effects of climate risk on primates globally: New perspectives on conservation priorities

**DOI:** 10.1126/sciadv.aeg0031

**Published:** 2026-08-21

**Authors:** Yang Teng, Yueqi Yin, Ying Shen, Yue Sun, Jiawen Liu, Jiwei Qi, Xiaochen Wang, Mingyi Zhang, Paul A. Garber, Zuofu Xiang, Xumao Zhao, Ming Li

**Affiliations:** ^1^ State Key Laboratory of Animal Biodiversity Conservation and Integrated Pest Management, Institute of Zoology, Chinese Academy of Sciences, Beijing 100101, China.; ^2^ University of Chinese Academy of Sciences, Beijing 100049, China.; ^3^ School of Biological Sciences, Guizhou Education University, Guiyang 550018, China.; ^4^ International Centre of Biodiversity and Primate Conservation, Dali University, Dali, Yunnan 671000, China.; ^5^ Department of Anthropology and Program in Ecology, Evolution, and Conservation Biology, University of Illinois, Urbana, IL, USA.; ^6^ College of Forestry, Central South University of Forestry and Technology, Changsha, Hunan 410004, China.; ^7^ College of Ecology, Management Center of Scientific Observations, Lanzhou University, Lanzhou, Gansu 730000, China.

## Abstract

Rapid climate change poses a severe threat to biodiversity, and phylogenetic diversity—a key metric capturing evolutionary uniqueness and adaptive potential—is critical for conservation. Integrating a well-resolved phylogenetic tree of 424 primate species, we combined spatial analysis and climate risk modeling to explore global spatiotemporal patterns of primates and assess their vulnerability under future climate scenarios. The results suggest a pronounced latitudinal pattern in primate distribution, with isothermality and annual mean precipitation as key drivers. Primate species in the American tropics (Mexico, Central, and South America) and southern China showed later divergence times and lower phylogenetic diversity, emerging as neo-hotspots for primates. Under future climate change, species in these neo-hotspots will face higher climate risks than those in paleo-hotspots of primate diversity. In addition, high human pressure, high climate risk, and limited protected area coverage increase species and population survival risks in hotspot regions. Overall, this global study deepens our understanding of biodiversity conservation under current climate change scenarios and synergistically advances the preservation of numerous natural services essential to ecosystem health and human well-being. Our study provides a spatially explicit framework to fulfill Targets 3 and 8 of the Kunming-Montreal Global Biodiversity Framework.

## INTRODUCTION

Globally, human-activated environmental changes have caused a marked loss of biodiversity, raising concerns about the sixth mass extinction event (*1*). Over the past 400 years, more than 500 terrestrial vertebrate species have been declared or presumed extinct (*2*, *3*). Moreover, climate change has been widely recognized as one of the primary drivers of population extinctions and overall species loss over the next 100 years (*4*, *5*). In addition, the interplay between climate change and human activities further complicates biodiversity loss, which is expected to directly or indirectly negatively affect human health and well-being, threatening the functioning and sustainability of ecosystems and human societies worldwide (*6*). Therefore, conducting quantitative, comprehensive, and global-scale analyses to identify animal genealogies and geographic regions likely to decline due to climate change is essential for implementing the set of effective conservation actions needed to alleviate the continuing loss of global biodiversity.

Although numerous studies have examined the impacts of climate change on biodiversity, most have focused on shifts in species ranges and species richness, while other dimensions of biodiversity—such as phylogenetic diversity and evolutionary history—have received far less attention (*7*, *8*). Given that closely related species commonly share similar ecological requirements and are expected to maintain phylogenetic conservatism in response to climate change (*9*), taxa with different evolutionary histories are likely to face alternative levels of risk under climate change. In particular, evolutionarily unique species should be assigned higher conservation priority because they represent irreplaceable evolutionary history and adaptive potential (*10*, *11*). Unlike species richness, which only counts the number of species, phylogenetic diversity quantifies the total evolutionary differences among species in a community, preserving deep phylogenetic relationships and unique genetic adaptability, thereby enhancing resilience to future changes (*12*). Incorporating phylogenetic diversity also serves to avoid the nonrandom extinction of ancient or unique lineages, reducing functional redundancy and enhancing the response ability to future climatic conditions. Phylogenetic diversity has high information value in global conservation priorities, climate adaptation planning, and protection of evolutionary potential. Identifying and protecting long-term recovery ability and uniqueness have greater conservation importance than short-term maximization of species numbers (*13*).

Phylogenetic diversity is defined as the sum of the branch lengths of a phylogenetic tree connecting all species distributed within a given area (*14*). By capturing the temporal scale of species evolution and history, phylogenetic diversity can inform the identification of priority regions for biodiversity conservation (*15*–*17*). However, previous studies have largely focused on the phylogenetic relationships of primates and the estimation of their divergence times, with little attention paid to the spatial patterns of phylogenetic diversity or its integration with climate risk assessments. At the same time, existing protected area networks and global conservation priority schemes primarily focus on species richness as an indicator, with virtually no consideration given to phylogenetic diversity. This approach risks driving primate lineages with unique evolutionary status toward extinction. Existing research indicates that areas with high phylogenetic diversity do not always overlap with areas of high species richness, meaning that planning based solely on species richness may overlook regions that are evolutionarily unique but have lower diversity (*15*, *18*). While species richness focuses on quantity, phylogenetic diversity focuses on the quality of biodiversity. Conserving areas of high phylogenetic diversity safeguards unique evolutionary lineages that cannot be identified by species richness alone, thereby reducing the risk of losing irreplaceable evolutionary potential. Therefore, monitoring the levels and spatial distribution of phylogenetic diversity is essential for effective biodiversity conservation. Furthermore, phylogenetic diversity reflects the evolutionary history of all species communities. Given that communities with differing phylogenetic diversity may exhibit alternative responses to climate change (*19*, *20*), evaluating phylogenetic diversity indices is importantly contribute to a more comprehensive approach to the conservation of the Tree of Life in the face of climate change.

Conservation planning targets the identification of priority sites for protection, namely, legally designated protected areas that conserve biodiversity and cultural resources (*21*). Protected areas are vital for sustaining global biodiversity and mitigating diverse anthropogenic pressures including climate change (*22*–*24*). Most existing protected-area networks were created to conserve present-day biodiversity and ecosystems (*25*, *26*). As for primates, ∼60% of primate species are currently threatened with extinction, a large number of core habitats lack protection, and assessments of the effectiveness of protected areas remain inadequate. At the same time, relevant habitat assessments have been conducted for only a limited number of species and mainly at regional scales. There has been no formal evaluation of the degree to which existing protected areas continue to play an effective role in conservation protection under intensifying climate change, and this can substantially limit the implementation of the Kunming-Montreal Global Biodiversity Framework’s target of “30 × 30” (*27*). Moreover, climate change is accelerating or forcing shifts in species’ distributions as species track emerging suitable habitats, which may limit the ability of currently protected areas to effectively buffer biodiversity against climate-driven threats (*28*, *29*). While the Kunming-Montreal Global Biodiversity Framework identifies conservation targets as essential requirements for halting ecological degradation and reversing biodiversity loss, relying solely on existing climate-vulnerable protected area networks, even if area targets are met, will likely fall short of achieving the core objective of effective conservation (*27*). Therefore, a global-scale analysis is needed to assess the future conservation effectiveness of protected areas under alternative climate scenarios and to provide timely recommendations to meet changing conservation management strategies.

Species distribution models (SDMs) are widely used to predict species’ responses to climate change (*30*, *31*). With the increasing availability of large-scale species distribution datasets and environmental layers, reliable predictions of species’ geographic ranges have become possible (*32*, *33*). Currently, SDMs have been widely applied to various taxonomic groups, including birds, amphibians, and terrestrial mammals, to map species’ suitable habitats, predict distribution shifts, and identify climate refugia (*23*, *34*, *35*). However, compared to other taxonomic groups, the application of SDMs in primate research remains relatively limited. Although regional studies in Africa, Asia, and elsewhere have analyzed habitat loss and climate threats to local primate populations, there is still a lack of long-term climate risk assessments for primates on a global scale (*36*, *37*). SDM-based analyses can elucidate how environmental change has influenced and will continue to influence the historical and future dynamics of species distributions (*30*, *31*). Consequently, simulating current and future species distributions to predict climate-related risks, while simultaneously evaluating the role of protected areas under climate change and formulating effective conservation strategies, has become a central tool to protect biodiversity (*25*, *38*, *39*).

Nonhuman primates (hereafter primates) are among the most species-rich and threatened mammalian clades worldwide and constitute an iconic component of tropical ecosystems. Primate assemblages sustain key ecological processes and provide multiple ecosystem services that benefit human communities (*37*, *40*, *41*). The evolution of primates, their feeding ecology, role in promoting forest regeneration, and their geographical distribution are closely related to the diversification of angiosperms (flowering plants), which are regarded as the main food sources by many animals and humans (pollen, nectar, fruits, and seeds) (*42*). Many primates have been confirmed or suspected to be important pollinators because they have adaptable and nondestructive behaviors of feeding on flowers and nectar (*43*, *44*). At the same time, their relatively large body size enables them to disperse small and large seeds over long distances, thereby promoting forest regeneration (*44*). Moreover, the loss of primates as seed dispersers will have a significant negative impact on human communities in the same ecosystem. For example, in the western region of Côte d’Ivoire, 48% of the plants whose seeds are primarily dispersed by primates and 42% of primate dispersed plants in Uganda have economic or cultural value for local residents (*45*). In southern Nigeria, rural residents rely on collecting fruits and seed plants dispersed by primates for food. While direct anthropogenic activities such as hunting and habitat conversion currently threaten many primate species, long-term climatic fluctuations have emerged as one of the most overarching and pervasive drivers that will dictate the fundamental physiological and ecological boundaries of primate survival in the coming decades (*46*, *47*). Although direct empirical evidence of climate-change impacts on primate populations remains limited, recent analyses indicate that, relative to other mammals, primates have experienced among the greatest amount of recent historical exposure to climatic extremes such as cyclones and droughts (*47*, *48*). Unlike localized habitat threats, macroclimatic shifts operate on a global scale, fundamentally altering baseline habitat suitability and rendering entire regions uninhabitable regardless of local land preservation. Primate species with restricted geographic ranges and slow life histories are extremely vulnerable to shifts in ecological conditions and are thus expected to experience the most severe impacts under climate change (*49*, *50*). In this regard, Li *et al.* (*51*) found that future climate change will adversely affect several primate species in China. Among these, the Critically Endangered white-headed langur (*Trachypithecus leucocephalus*) and the Endangered white-cheeked macaque (*Macaca leucogenys*), both with extremely restricted distributions, are predicted to experience severe loss of suitable habitat and a high risk of extinction. Furthermore, climate-change–induced shifts often outpace the dispersal capabilities of primates, leading to severe negative demographic and genomic consequences (*52*, *53*). For example, Teng *et al.* (*54*) showed that under future climate change, Near Threatened Tibetan macaque (*Macaca thibetana*) populations in eastern China, particularly those in Huangshan, Anhui Province, not only face a high risk of habitat fragmentation but also exhibit high genomic vulnerabilities. Moreover, as climate change alters local resource availability and ecosystem compositions, it introduces novel predators, pathogens, and competitors (*55*), along with increasing the frequency and intensity of floods and droughts, negatively affecting population health, reproductive performance, and survival rates (*56*, *57*). Therefore, evaluating the distinct and long-term risks posed by climate change, particularly through the lens of evolutionary history and phylogenetic diversity, is a critical step for delineating future conservation priority regions and ensuring the persistence of the primate Tree of Life. However, although dynamic processes such as diffusion limitations, a time lag in range migration, and changes in interspecies interactions are undoubtedly the core factors of these ecological impacts, our global-scale analysis focuses on quantifying static climate exposure risks by comparing current and future climate conditions. This is due to the limited high-resolution, species-specific data on diffusion and biological interactions for most primate species. To avoid introducing significant model uncertainties, our results provide a robust and conservative baseline assessment. If a species is identified as high risk within this static framework, its actual vulnerability is likely to be even greater, due to dynamic pressures and anthropogenic barriers that could not be taken into account.

Our research aims to explore quantitative gaps in two goals of the “Kunming-Montreal Global Biodiversity Framework” (Target 3: expand protected area coverage to 30%, and Target 8: minimize the impact of climate change on biodiversity) (*58*, *59*). By identifying protected hotspots that are currently not covered by protected areas and are vulnerable to climate change, we provide a quantitative decision-making basis to accomplish these key policy goals. Therefore, by analyzing a large existing dataset and integrating robustly dated phylogenetic trees with species distribution data and supplementing these with spatial analysis and climate risk modeling, we focused on identifying patterns of phylogenetic diversity and conservation priority regions for global primate species under models of future climate change. The results show that (i) primate distributions exhibit distinct latitudinal patterns; (ii) isothermicity and annual average precipitation are key drivers; (iii) identify hotspots of high primate species richness and high phylogenetic diversity; (iv) predict the static climate suitability risks for primates under future climate scenarios; and (v) identify priority regions for primate biodiversity conservation. The methods and goals of studying species richness, phylogenetic diversity, and conservation strategies to mitigate the effects of climate change on primates used here can be successfully applied to other vertebrate taxa.

## RESULTS

### Spatial patterns of primate diversity

**Fig. 1.**
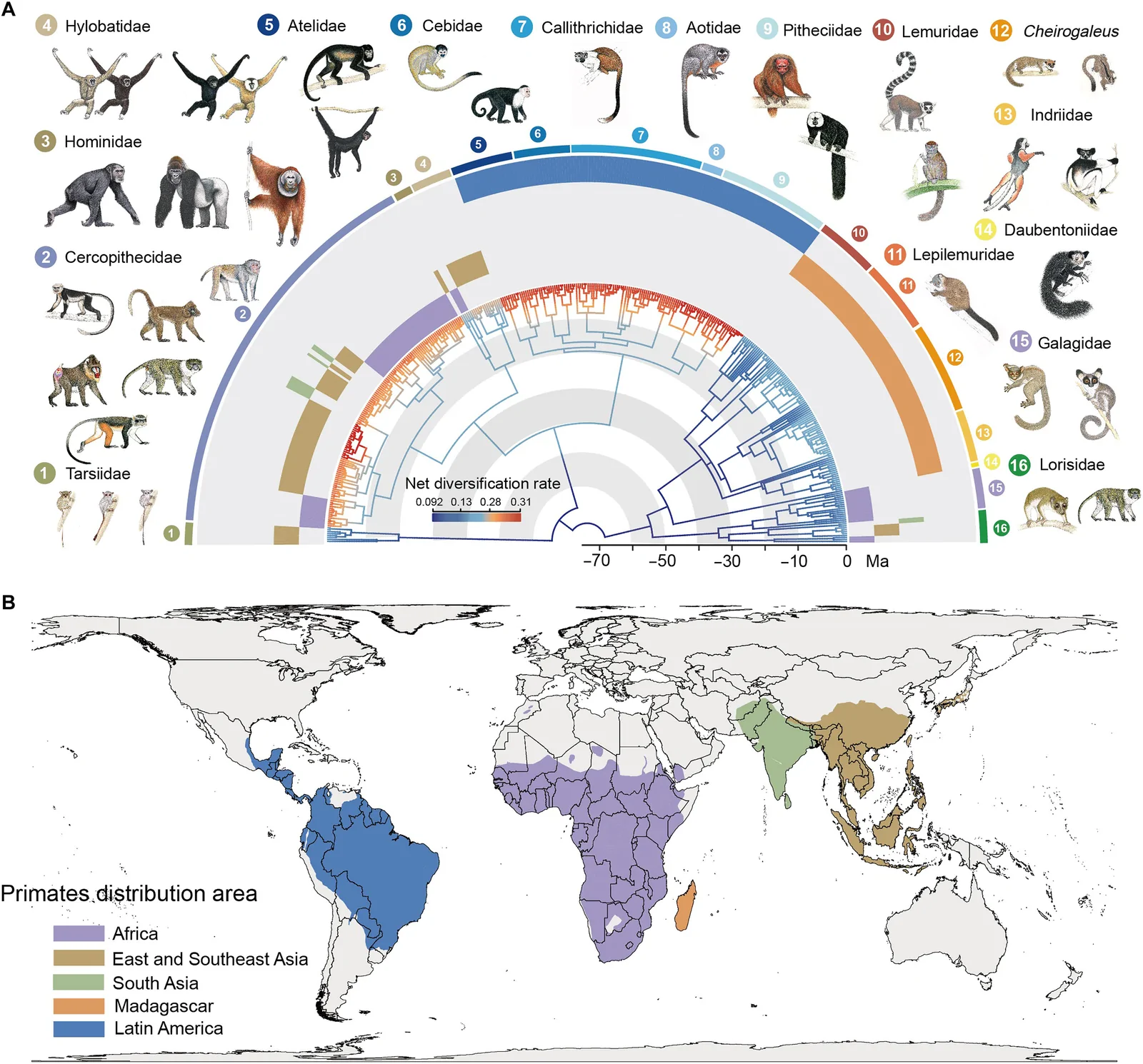
Time-calibrated phylogenetic tree of 424 primate species, showing the biogeographic distribution of extant species and species diversification rates estimated by Bayesian analyses of macroevolutionary mixtures. (**A**) The colored bands in the outer ring represent the biogeographic distribution of each taxon in the tree. Branch colors reflect the average posterior net diversification rate from BAMM’s birth-death analysis, ranging from blue (low diversification rate) to red (high diversification rate). [Illustrations copyright 2025 S. D. Nash/International Union for Conservation of Nautre (IUCN) Species Survival Commission (SSC) Primate Specialist Group]. (**B**) Biogeographic distribution of extant primates: Blue represents the American tropics; purple represents mainland Africa; orange represents Madagascar; green represents South Asia; brown represents East and Southeast Asia.

On the basis of the coding sequences of five mitochondrial genes (*Cytb, CO1, ND1, 12S rRNA*, and *16S rRNA*), we constructed a phylogenetic tree of primate species and created a species-level evaluation of phylogenetic diversity. In total, phylogenetic diversity was assessed for 424 primate species, which collectively represents 80.5% of all recognized (*n* = 527) primate species. This dataset covered every primate genus, with at least one usable mitochondrial gene sequence available for each species (fig. S2 and table S1). Using a maximum-likelihood phylogenetic tree, together with 40 divergence-time constraints from the TimeTree database and literature (table S2), divergence times for each primate lineage were estimated. The most recent common ancestor of all primates was inferred to have originated ∼66.9 million years ago (Ma) at the Cretaceous-Paleogene boundary, an estimate closely matching those from recent large-scale mammalian phylogenetic studies and primate phylogenetic analyses (*60*, *61*) (Fig. 1A). In addition, the divergence between monkeys of the American tropics (Mexico, Central, and South America) and Old World monkeys (Africa and Asia) was dated to ∼40.2 Ma, with subsequent diversification of primates of the American tropics beginning some 19.7 Ma and proceeding at a relatively high rate of diversification (Fig. 1A).

**Fig. 2.**
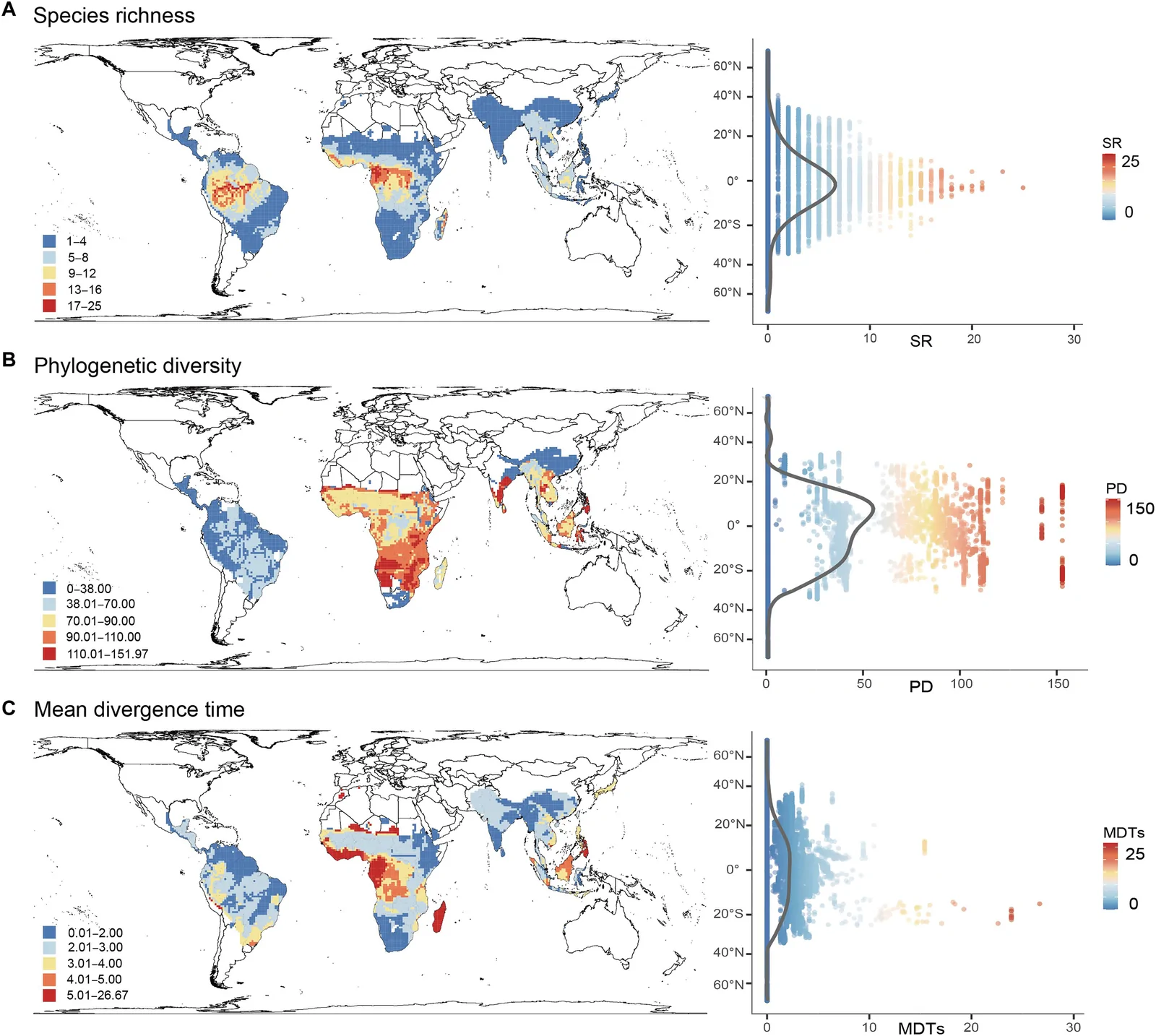
Distribution patterns and latitudinal distribution of primate diversity. (**A**) SR. (**B**) Phylogenetic diversity (PD). (**C**) MDT. The scatter plot on the right displays map data, with all latitudinal gradient curves fitted by generalized additive model (GAM) regression models. Maps are rendered using the Berman equal-area projection.

A total of 98,103 occurrence records for 424 primate species were compiled from field surveys and published distribution datasets. Species ranges were subsequently estimated and aggregated to a 1° × 1° grid (Fig. 1B and fig. S1). Across 18,389 occupied grid cells, spatial patterns of species richness (SR), phylogenetic diversity, and mean divergence time (MDT) were quantified for these 424 species. Species richness exhibited a pronounced latitudinal gradient, with peak richness between 20°N and 20°S, primarily focusing on regions of Amazonian and African tropical rainforests (17 to 25 species per grid cell). Elevated richness was also observed on Madagascar (13 to 16 species per grid cell), in Indonesia (9 to 12 species per grid cell), and in southwestern China (9 to 12 species per grid cell) (Fig. 2A). Analyses based on the species-level phylogenetic tree revealed marked spatial variation in phylogenetic structure. Africa, particularly southern Africa, Madagascar, Southeast Asia, and southwestern China (such as Yunnan Province) exhibited phylogenetic overdispersion, corresponding to relatively high phylogenetic diversity, whereas the American tropics showed pronounced phylogenetic clustering and comparatively low phylogenetic diversity (Fig. 2B). MDT distribution patterns revealed more ancient genealogies in Central Africa, Madagascar, and Southeast Asia, whereas species differentiation occurred later in southern China and the American tropics (Fig. 2C).

### Relationships between environmental factors and species richness pattern

**Fig. 3.**
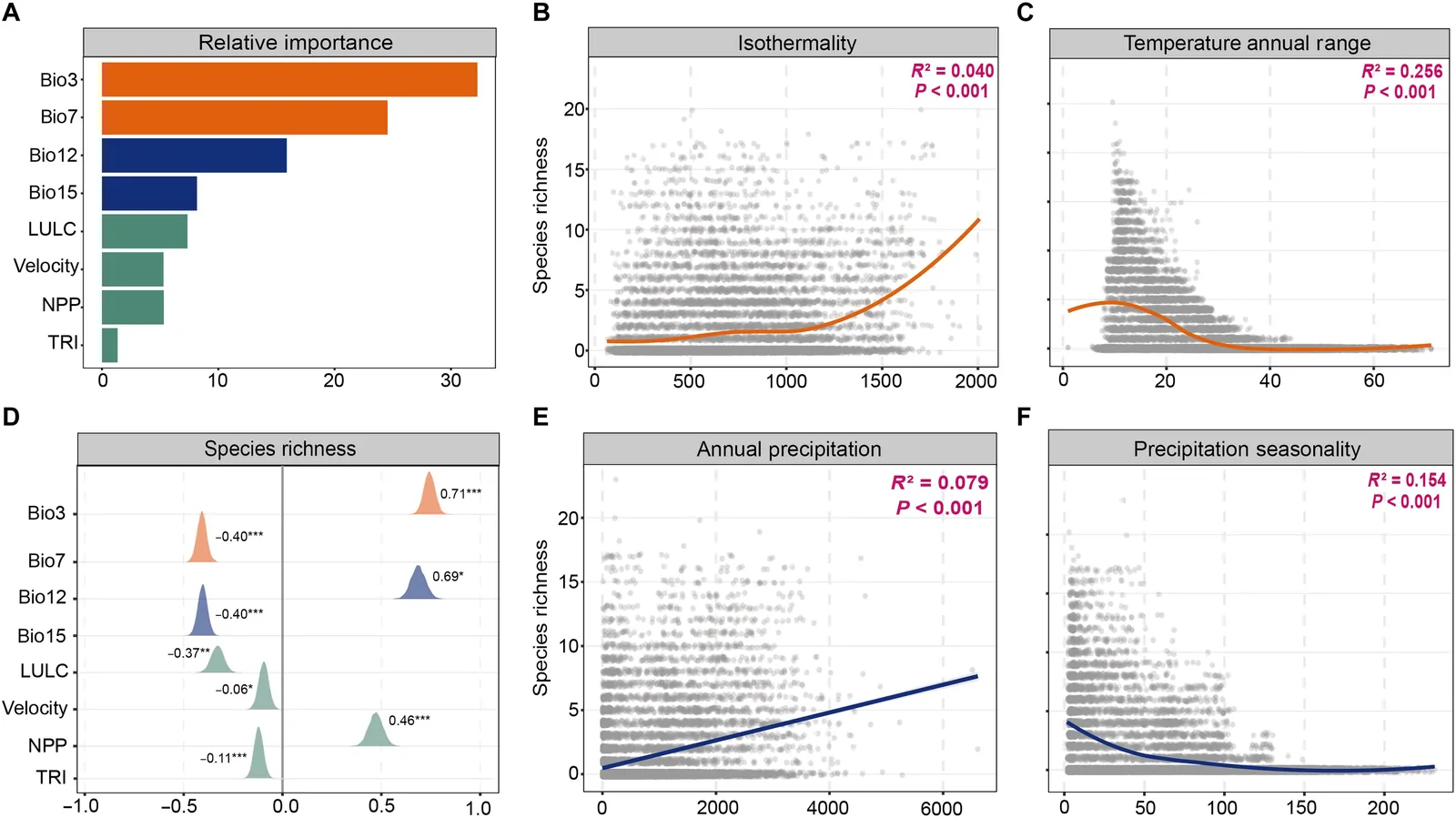
Drivers of primate species richness distribution pattern. (**A**) The relative importance of eight fixed effects in the final negative binomial generalized linear mixed model, adjusted for sampling bias, (**B**) isothermal, (**C**) annual temperature range, (**E**) annual mean precipitation, and (**F**) coefficient of variation in precipitation. Lines indicate median predicted values, and shaded areas represent 95% confidence intervals. (**D**) Regression coefficients for each environmental factor, with 95% confidence intervals on either side of each point; * denotes significant effect level (*P < 0.05; **P < 0.01; ***P < 0.001).

To evaluate the influence of environmental factors on spatial patterns of species richness, we fitted a negative binomial generalized linear mixed-effects model, and the final model was constructed by retaining fixed effects with a relative importance > 30%. The final model included seven predictors: isothermality (BIO3), annual temperature range (BIO7), annual precipitation (BIO12), precipitation seasonality (BIO15), climate velocity (velocity), net primary productivity (NPP), the terrain ruggedness index (TRI), and land use and land cover (LULC) (table S3). No significant spatial autocorrelation was detected for these variables, indicating that spatial autocorrelation had only a limited effect on model results. Among all predictors, species richness showed the strongest associations with temperature-related variables, particularly isothermality and annual temperature range (Fig. 3). Isothermality emerged as the most important environmental driver of species richness, accounting for 35.16% of the total explained environmental contribution, followed by annual temperature range (25.47%), annual precipitation (16.83%), and the coefficient of variation of precipitation (9.13%) (Fig. 3A). Isothermality and annual precipitation were positively associated with species richness (Fig. 3, B, D, and E), whereas annual temperature range and the coefficient of variation of precipitation were negatively associated with species richness (Fig. 3, C, D, and F). These results confirm that areas with stable temperatures and abundant annual precipitation provide sufficient resources to support species-diverse primate communities (*62*, *63*).

### Hotspot identification and assessment of climate risk

**Fig. 4.**
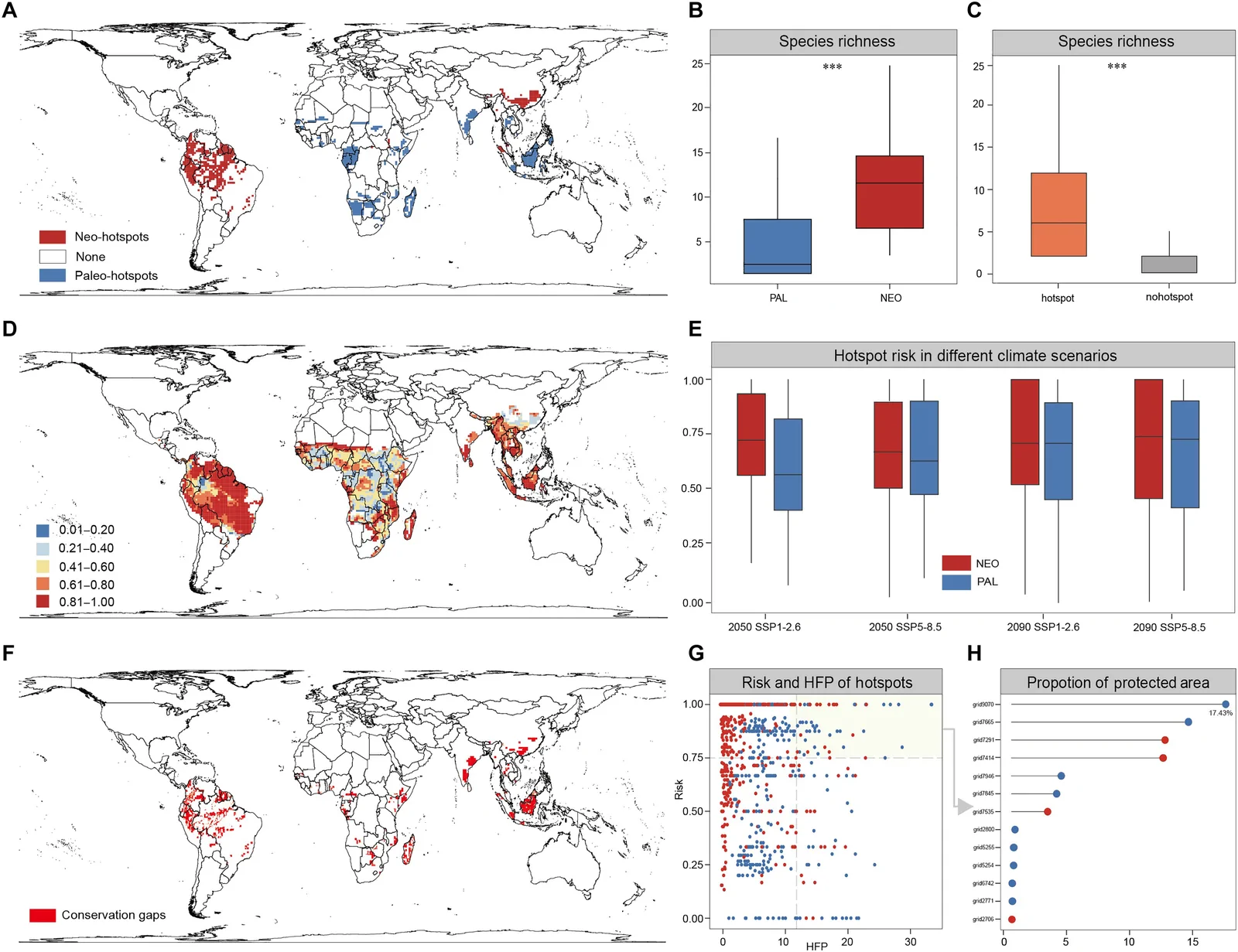
Distribution of hotspots and climate risk predictions. (**A**) Distribution of hotspots based on three measurement methods, with red indicating neo-hotspots and blue representing paleo-hotspots. (**B**) Comparison of species richness between neo-hotspots and paleo-hotspots. (**C**) Comparison of species richness between hotspots and nonhotspots. (**D**) Projected proportion of primate species facing high risks from climate and land-use changes under SSP5-8.5 climate scenarios, with blue to red indicating increasing risk levels. (**E**) Proportion of primate species at risk in neo-hotspot regions versus paleo-hotspot regions under different future climate scenarios. (**F**) Distribution of primate conservation gaps. (**G**) Spatial overlap between the proportion of climate risk and the human footprint index in hotspot grid cells under the SSP5-8.5 climate scenario. (**H**) The proportion of conservation coverage for each high-risk hotspot grid cell.

To control for the confounding effect of species richness, we calculated standardized effect sizes of phylogenetic diversity (*SES-PD*) using a randomization procedure. Then, grid cell *SES-MDT* and residual phylogenetic diversity were quantified. The three metrics were jointly used to identify areas where primate phylogenetic diversity was substantially higher or lower than expected, thereby delineating primate hotspot regions. The results indicate substantially higher phylogenetic diversity in Central Africa (primarily comprising Gabon and the Republic of the Congo), Namibia, Madagascar, southern India, and Indonesia, involving a total of 569 grid cells. These regions appear to preserve a large number of ancient primate lineages, serving as veritable “evolutionary museums,” termed paleo-hotspots (Fig. 4A) (*64*). By contrast, regions identified as neo-hotspots, primarily Colombia, Ecuador, Peru, northern Brazil, and southern China (including Guangxi, Guizhou, Hunan, Jiangxi, and northern Yunnan Provinces), exhibited lower phylogenetic diversity across 499 grid cells. This indicates that these areas are hotspots for recent speciation events and are predominantly dominated by younger lineages. We regard these regions as “evolutionary cradles,” termed neo-hotspots (Fig. 4A) (*64*). Species richness in neo-hotspots was significantly higher than in paleo-hotspots (*P* < 0.001; Fig. 4B), and richness in all hotspot regions was significantly higher than in nonhotspot regions (*P* < 0.001; Fig. 4C), indicating that hotspots, especially neo-hotspots, are important locations for biodiversity conservation (*58*).

We defined a species’ climate risk as the difference between a grid cell’s exposure to future climate conditions and the boundaries of that species’ climatic niche (*65*). To assess climate risk, we calculated the proportion of risk within a grid cell as the percentage of species within that cell experiencing climate risk (*66*). Our results indicate that under the 2050 SSP1-2.6 scenario, future changes in climate and land use were predicted to result in higher proportions of species facing climate risk in the American tropics (including Colombia, Peru, and southern Brazil), southern Africa (including Zambia), Madagascar, southern India (including the states of Tamil Nadu and Andhra Pradesh), and Southeast Asia (including Myanmar, Thailand, and Indonesia) (figs. S9 and S10). Under the 2090 SSP5-8.5 scenario, nearly all primate habitats in the American tropics will experience high risks from climate and land-use change, apart from the Amazon region (Fig. 4D). In addition, these results suggest that under the 2050 SSP1-2.6 and 2090 SSP5-8.5 scenarios, 59.05% (^310^/_525_ grid cells) and 64.57% (^339^/_525_ grid cells) of species, respectively, will be under high climate risk. Under the 2050 SSP1-2.6 scenario, 124 primate species in neo-hotspots and 109 in paleo-hotspots are expected to face high climate risk. On the basis of the 2090 SSP5-8.5 scenario, the number of high-risk species in neo-hotspots is expected to increase to 159, while in paleo-hotspots, it is expected to reach 125. Across our analyses, the proportion of species at high climate risk in neo-hotspots consistently remained higher than in the paleo-hotspots (Fig. 4E).

We also identified priority conservation regions that are characterized by high primate species richness, high phylogenetic diversity, high climate risk, and inadequately covered by existing protected areas. These regions were primarily distributed in northern Brazil (the state of Amazonas), Peru (the Amazonian region of Loreto), Madagascar (Toamasina and Tuléar), southern India (including the states of Tamil Nadu and Andhra Pradesh), Indonesia (South Sumatra, West Kalimantan, Central Kalimantan, and East Kalimantan), eastern China (Anhui Province), and southern China (e.g., Guangxi Province) (Fig. 4F). In addition, we assessed the spatial overlap between the human footprint index and grid cells with a high proportion of climate-risk species. Under the 2090 SSP5-8.5 scenario, high-risk hotspots (human footprint index ≥ 12 and a grid cell climate risk ratio ≥ 0.75) were predominantly concentrated in neo-hotspots (encompassing 42 hotspot grid cells). In contrast, 27 hotspot grid cells occurred in paleo-hotspots (Fig. 4G). Last, by overlaying protected areas with high climate risk grid cells, we projected that under the 2090 SSP5-8.5 scenario, protected area coverage for all high climate risk hotspots will not exceed 17.43% (Fig. 4H), falling far short of the Global Biodiversity Framework (GBF) Target 3 requirement of 30%. This quantitative gap indicates that nearly half of the required conservation expansion to achieve GBF Target 3 should be strategically directed toward these identified primate hotspots to ensure the persistence of the primate Tree of Life under future climate scenarios. Overall, these high–climate-risk hotspots contain limited protected areas and experience intense human pressures from population growth, road construction, and rapid urbanization. Furthermore, most species within these hotspots will face higher climate risks, which is more pronounced in neo-hotspots.

## DISCUSSION

Climate change has triggered more frequent and intense extreme weather events, including cyclones, extreme heat, droughts, wildfires, and flooding, which harm both human communities and wildlife survival (*9*, *67*, *68*). This includes many of the more than 300 species of currently threatened nonhuman primates (*69*). Given the critical roles that prosimians, tarsiers, monkeys, and apes play in maintaining ecosystem functions (*37*, *70*) and their contribution as umbrella species across many forested habitats (*70*), identifying priority primate conservation regions in response to future climate change is essential for guiding actions to prevent severe biodiversity loss (*71*). By reconstructing primate phylogenies and integrating analytical indicators such as species richness, phylogenetic diversity, mean time to divergence, diversification rates, and climate risk, we explored the spatiotemporal patterns of primate speciation and predicted priority conservation regions to protect primates under expectations of future climate change. Our results indicated strong latitudinal patterns in primate spatial diversity, with peak species richness occurring between 20°N and 20°S. We also found that regions expected to experience stable and relatively mild temperatures and abundant precipitation represent the most beneficial locations for primate survival. Last, we found areas of northern Brazil (the state of Amazonas), Peru (the region of Loreto), Madagascar (Toamasina and Tuléar), southern India (including the states of Tamil Nadu and Andhra Pradesh), Indonesia (South Sumatra, West Kalimantan, Central Kalimantan, and East Kalimantan), eastern China (Anhui Province), and southern China (Guangxi Province) should be designated as priority conservation regions to support and protect primate populations under future climate change.

Phylogenetic diversity represents the accumulation of evolutionary adaptations within species over time and correlates with species’ evolutionary potential for continued adaptive change (*16*). Prioritization based on phylogenetic diversity is most valuable when conservation efforts aim to preserve adaptive potential, prevent the loss of deep evolutionary lineages, and establish climate-resilient networks of protected areas (*16*, *17*). In contrast, approaches that focus solely on species richness may satisfy short-term statistical needs but fail to capture evolutionary uniqueness. On the basis of a global-scale assessment, we found that Cameroon, Gabon, Congo, and Madagascar in Africa, along with Indonesia and southwestern China (e.g., Yunnan Province) in Asia, exhibit both high species richness (Fig. 2A) and high phylogenetic diversity (Fig. 2B), highlighting these regions as high priority conservation areas. After controlling for species richness effects, we further identified key regions with high and low phylogenetic diversity, revealing primate “evolutionary museums and cradles” (Fig. 4A) (*65*). Notably, we identified inconsistencies between species richness and phylogenetic diversity across regions. Despite high primate species richness in South America, phylogenetic diversity remains low (Fig. 2), potentially attributable to evolutionary rate heterogeneity (*72*). Primates of the American tropics appear to have rapidly generated closely related species through accelerated speciation within a relatively short evolutionary time frame (*73*). Because of their close phylogenetic relationship, these species contribute little to phylogenetic diversity but contribute substantial to species richness within the region (*74*).

The distinct geographic delineations of primate paleo-hotspots and neo-hotspots operate under fundamentally divergent evolutionary and environmental mechanisms (*64*, *72*). Paleo-hotspots, predominantly localized in Central Africa and Madagascar, function as evolutionary museums. Mechanistically, these regions are heavily coupled with long-term Cenozoic and Quaternary climatic stability, acting as massive macrorefugia where low historical climate velocity permitted ancient lineages to persist uninterrupted by widespread lineage turnover (*75*). Conversely, neo-hotspots across the American tropics and southern China act as active evolutionary cradles. The emergence of these cradles is driven by highly dynamic environmental and geological historical processes—such as the Andean orogeny and the complex multitiered topography of southern China. This environmental heterogeneity catalyzed localized geographic isolation, creating abundant vacant ecological niches that stimulated high net diversification and recent speciation rates (*76*–*78*). In the context of global climate change, these different hotspots should adopt different conservation paradigms: For instance, in paleo-hotspots, due to the excessive dispersion and irrelevance of the system’s evolutionary diversity, strict requirements should be imposed for the static protection of large contiguous forest areas. The goal is to protect the ancient evolutionary history from the impacts of land use pressure and fragmentation (*11*, *15*). In neo-hotspots, younger species lineages have limited geographical distribution ranges and narrow climatic ecological niches, and static protection is no longer sufficient. Therefore, conservation measures must shift to dynamic landscape connectivity, prioritize the rapid expansion of natural vegetation buffer zones, and promote the active migration of species through the construction of artificial migration corridors to facilitate their movement within the human-dominated matrix areas (*36*, *79*).

We also examined the influence of abiotic and biotic factors driving primate spatial distribution patterns at the scale of individual grid cells. Overall, the effects of these factors on primate species distribution patterns align with ecological expectations. The habitats of primate species are mainly located in forested landscapes, and isothermality and high annual mean precipitation exerted significant positive effects on species richness (Fig. 3D). This might be because the stable temperature pattern minimizes the metabolic cost of thermoregulation for primates, allowing more energy to be allocated to behaviors such as reproduction and foraging. At the same time, abundant precipitation not only increases the availability of food resources but also maintains the multilayered tropical forest structure. This structure maximizes the available ecological niches and ensures the supply of various food resources, thereby promoting species survival, population expansion, and species formation (*80*, *81*). NPP also was found to contribute positively to primate species richness (*81*). In contrast, greater seasonal and year-to-year variability in temperature, precipitation, climate change rate, and an increase in the TRI were found to negatively affect primate species richness (Fig. 3D) (*82*). This might be because most primates are nonmigratory animals that rely on complex forest structures. The significant temperature difference could lead to physiological heat stress and a more severe energy bottleneck. At the same time, the larger seasonal precipitation pattern might trigger a dry season characterized by leaf fall, resulting in a shortage of food resources and ultimately reducing the local species diversity.

Hotspots of species distribution provide important habitats for species conservation. Protecting these areas can help prevent the extinction of evolutionarily unique lineages (*83*). However, the strong association between climate variables and primate diversity is concerning. Even under moderate emission scenarios, projected changes in temperature and precipitation are poised to exceed the climatic suitability thresholds for many primate species across many hotspot regions (*36*, *51*). Under the SSP1-2.6 scenario, more than 70% of primate species in hotspots face climate risks (fig. S9), with this proportion trending higher under the high-emission SSP5-8.5 scenario. When contextualized within broader vertebrate macroecology, our climate risk predictions mirror some global commonalities while exposing critical primate-specific risks. Global gap analyses for ectothermic vertebrates, such as amphibians and reptiles, consistently indicate that accelerating climate velocity under high-emission scenarios will drive widespread range mismatches within current reserve boundaries (*36*). Similarly, large-scale avian assessments demonstrate substantial spatial uncoupling between taxonomic species richness and phylogenetic diversity across global biomes (*18*). However, our primate-centric model reveals a highly specialized vulnerability fingerprint. While avian and reptilian climate risks frequently peak at latitudinal extremes or within volatile arid zones, global primate vulnerability is tightly constrained within an equatorial band governed primarily by isothermality (*47*). The paramount novelty of our findings lies in the phylogenetic asymmetry of this risk. In contrast to global patterns in seed plants or terrestrial birds where paleo-refugia are often flagged as highly threatened due to old lineage endemism, primates show the inverse paradox: Under both the SSP1-2.6 and SSP5-8.5 trajectories, the proportion of species confronting severe climatic niche boundary breaches is substantially amplified within young neo-hotspots compared to paleo-hotspots (*62*). This reveals that the products of recent adaptive radiations inhabit hyperspecialized, narrow thermal baselines, making them evolutionarily brittle and disproportionately sensitive to even minor shifts in seasonal temperature envelopes compared to their ancient counterparts (*73*). Our static climate risk assessment likely underestimates real-world vulnerability by omitting key dynamic ecological processes. Most primates exhibit limited dispersal ability, slow life histories, and strong habitat specialization, which will restrict their capacity to track shifting climate niches, particularly in human-fragmented landscapes (*52*, *53*). Range-shift lags and habitat fragmentation will further trap populations in increasingly unsuitable areas, subjecting them to an elevated extinction risk. In addition, climate change can disrupt critical species interactions, including pollination, seed dispersal, predator-prey interactions, and trophic relationships, while increasing exposure to novel pathogens and competitors (*55*). These processes collectively mean that the climate risks we identify for neo-hotspots are likely to be conservative estimates, with actual impacts potentially more severe. Future work integrating species-specific dispersal models and biotic interaction data will help refine these projections.

We found that over the coming decades, neo-hotspots will represent areas of significant primate species richness (Fig. 4B). However, under future climate change models, the number of species exposed to multiple climate risks in neo-hotspots will increase compared to those inhabiting paleo-hotspots, adding to their vulnerability. High human population pressure, high climate risk, and lack of protected area coverage further exacerbate the challenges faced by primates across all hotspots, especially neo-hotspots. Therefore, targeted expansion and protection of natural vegetation and the creation of migration corridors that form protected area networks in areas adjacent to all biodiversity hotspots are needed to mitigate current and future climate risk (*84*).

Understanding the effectiveness of existing protected areas and the location of future protected areas, as part of a foundational strategy for species conservation in response to climate change, is widely recognized as one of the primary challenges faced by conservation biologists and government leaders in safeguarding biodiversity (*79*, *85*–*87*). In the current study, we identified several critical regions of high primate diversity that currently lack a sufficient number of protected areas containing suitable native habitat to protect their threatened primate populations. These areas include northern Brazil, Peru, Madagascar, southern India, Indonesia, eastern China, and southern China (Fig. 4F). Many of these countries face severe resource constraints in promoting environmental protection. In addition, it is often mistakenly assumed that biodiversity conservation and economic development are not compatible (*88*, *89*). Healthy ecosystems provide significant benefits to local human communities including water retention, carbon sequestration, reduced soil erosion, sustainable access to forest products such as food, wood, and medical plants, barriers to zoonotic disease transmission, and economic opportunities associated with ecotourism (*70*). Thus, effective conservation strategies for threatened primates and other taxa can make significant contributions to enhancing human well-being (*90*, *91*). Furthermore, to go beyond a purely conceptual framework, we explicitly linked our research results to the core global biodiversity goals of the Kunming-Montreal Global Biodiversity Framework (*92*). Specifically, we identified “protected blank areas” in such as the Amazon region, Madagascar, and southern China. Our spatial data provide decision-makers with the exact coordinates in which to create an expanded protected area network to achieve Target 3 (30 × 30). At the same time, our risk assessment model identified areas of high climate risk, which include the American tropics, southern Africa, and Southeast Asia. This can facilitate the design of “climate refugia” and migration corridors, which are the prerequisites for enhancing the climate resilience of biodiversity as outlined in Target 8.

However, although our results provide crucial decision support for the Kunming-Montreal Global Biodiversity Framework, it must be acknowledged that there are some inherent limitations that need to be taken into account to guide subsequent conservation research. First, our phylogenetic tree was constructed on the basis of five core mitochondrial gene loci. Although this method is highly efficient in constraining the large-scale diversification of 424 species and calibrating the genus-level topology, mitochondrial markers only reflect the strict maternal evolutionary history. Future research should shift toward whole-genome phylogenetics and pan-genomic architectures and directly monitor the genetic load related to climate adaptability. Second, our model mainly focuses on abiotic macroclimatic inputs, topographic features, and original primary productivity factors. However, in nature, climate change does not occur in isolation; it fundamentally disrupts biological interactions, including host-pathogen dynamics, predator-prey tracking, and the stability of the pan-genomic microbiome of nonhuman primates. Therefore, integrating these multilevel social-ecological and multiomics interaction networks will become the next key frontier area for global primate conservation prediction.

In conclusion, using a multi-indicator approach, we have advanced current understanding of the climate change impacts on global primate biodiversity and identified priority conservation areas to negate the harmful effects of future climate change on threatened primate populations. Moreover, the approach outlined here can be successfully applied to protecting other vertebrate taxa. By integrating phylogenetic information with expected species distributions under alternative future climate models, we identified primate hotspots such as Central Africa, Namibia, Madagascar, southern India, Indonesia, the American tropics, and southern China, which represent key regions that contain a large number of threatened and evolutionarily unique species. Furthermore, we also identified hotspots, including northern Brazil, Peru, Madagascar, southern India, Indonesia, and eastern and southern China, which should be prioritized for conservation under future climate change. We argue that existing protected areas and the creation of corridors to connect isolated protected areas will need to be expanded to protect primates and other species experiencing current and future climate risks. Overall, these results should raise widespread concern among evolutionary biologists, conservation biologists, and government officials and provide decision-making support for the Kunming-Montreal Global Biodiversity Framework.

## MATERIALS AND METHODS

### Phylogenetic reconstruction and divergence time estimation

We reconstructed the phylogenetic relationships of primates based on five mitochondrial genes (*Cytb, CO1, 12S rRNA, 16S rRNA*, and *ND1*). The choice of these specific mitochondrial markers was justified by their ubiquitous availability and high representation across all primate genera in public repositories (GenBank), which strikes an optimal balance between maximizing taxonomic coverage and minimizing the missing data matrix artifacts. Furthermore, these markers exhibit appropriate nucleotide substitution rates capable of resolving both deep-time divergence events and genus-level splits within mammalian lineages. To retrieve the sequences of these five mitochondrial genes from GenBank, we used the following steps: First, we downloaded the existing mitochondrial reference genomes and extracted the corresponding coding sequences for these genes. Second, for the remaining species, we obtained coding sequences from GenBank using the species’ Latin name and gene name. If multiple sequences exist for the same gene locus within a species, then we selected the sequence closest in length to the target gene. Last, we excluded short gene sequences with coding lengths less than 300 base pairs (*78*). This threshold was chosen because sequences shorter than 300 base pairs contain an insufficient number of phylogenetically informative sites. Incorporating such fragmented data introduces substantial phylogenetic noise, increases the risk of long-branch attraction artifacts, and leads to unstable nodal topologies or unresolved polytomies. Following these steps, data from 424 primate species were integrated, representing ∼80.5% of all primate species.

Next, we used MAFFT software (*93*) with default parameters to align and assemble the gene coding sequences, trimming sites with poor alignment quality at the ends. Then, the aligned sequences of these five gene sets were imported into the SequenceMatrix software (*94*) to construct a supermatrix, treating missing sites as missing data. Phylogenetic analysis of this supermatrix was performed using the maximum likelihood method in RAxML 8.2.12 (*95*), using the GTR + GAMMA model with 1000 bootstrap replicates. Each gene was treated as an independent partition, with the Sunda flying lemur (*Galeopterus variegatus*) and the Chinese tree shrew (*Tupaia belangeri*) serving as outgroups (*61*). On the basis of this phylogenetic tree, we estimated the divergence times of primate species using the penalized likelihood method implemented in TreePL software (*96*). Forty known divergence dates from the TimeTree database and literature (*97*) were selected as calibration points for the dating analysis (table S2). Calculations were performed under optimal parameter conditions using the “prime” option and the “through” analysis method.

### Speciation rate estimation

To study speciation and net diversification through time, we used the program Bayesian analyses of macroevolutionary mixtures v2.3.0 (BAMM) (*98*). BAMM models the evolutionary dynamics of lineages through time by defining distinct macroevolutionary cohorts that share common rates of speciation and extinction and are separated by other such cohorts because of diversification rate shifts. In addition, we considered the incomplete sampling of primate diversity, which may bias the estimation of diversification rates (*99*, *100*). Therefore, we set the proportion of stem completeness and the sampling proportion for each terminal node using the sampleProbsFilename parameter in BAMM (table S4). This parameter defines the path to an external input text file specifying nonrandom, clade-specific sampling probabilities. Unlike a global uniform sampling fraction, this parameter explicitly accounts for taxonomic heterogeneity in sampling completeness across different primate families and genera. The referenced input file contained a tabulated matrix matching specific terminal nodes and family-level stem clades to their respective empirical sampling fractions (calculated as the number of sampled species in this study divided by the total number of recognized species within that targeted clade), thereby preventing the underestimation of speciation rates in less-completely sampled lineages. Before BAMM analysis, outgroup taxonomic units were pruned using the setBAMMpriors function from the BAMMtools package. The chainSwapPercent. R function was used to select appropriate prior distributions, and Markov chain Monte Carlo chain parameters were used until satisfactory chain mixing and effective sampling size values for log-likelihood were achieved (*101*). To be conservative, we then discarded the first 20% of samples as burn-in.

### Species range reconstruction and environmental data collection

We collected 98,103 distribution site records for 424 species through field surveys, a review of the published literature, and the Global Biodiversity Information Facility database. We filtered the original observation records, removing duplicate coordinates, geographically unreasonable outliers, and records of captive individuals, to eliminate spatial deviations and low-quality data points. At the same time, to reduce the spatial autocorrelation of the sampling points, spatial sparsification processing was carried out before fitting the model, thereby reducing the clustering bias in the densely surveyed areas. For species with at least 10 geographic distribution site records, we used the “sdm” package in R to implement an ensemble SDM to predict species distributions with a high-performance cluster (*102*). We then used a 75% random sample of the initial data (presence-absence) as training data and evaluated this against the remaining 25% of the samples for each of the species (*8*). We repeated the split sampling 10 times to account for the uncertainty associated with data partitioning (*103*). In sum, we used 16 commonly used high model performance SDM algorithms in the ensemble models: maximum entropy (MaxEnt), random forests (Rf), generalized additive model (Gam), generalized linear model (Glm), Glmpoly, Glmnet, support vector machine (Svm), Maxlike, multivariate adaptive regression spline, classification and regression trees, multilayer perceptron, radial basis function, mixture discriminant analysis, recursive partitioning and regression trees, flexible discriminant analysis, and boosted regression trees (*104*). Then, the true skill statistic and the area under the receiver operating characteristic curve were used to evaluate model predictive accuracy and validate the robust performance of the 16 models. The values ranged from 0.5 to 1, with high values implying high levels of model prediction accuracy. For species with only one to two geographic distribution records, the International Union for Conservation of Nature (IUCN) spatial database range was used as their distributional range. Concurrently, we obtained distribution range data for all primates from the IUCN spatial database (www.iucnredlist.org/resources/spatial-data-download) to correct SDM results, ensuring the accuracy of species geographic ranges. The range of each species was originally available as a vectorized shapefile and was rasterized into a grid system with a 1° × 1° resolution (∼111 km by 111 km) using the Behrmann equal-area projection (*105*). Rasterized maps were double checked to confirm correspondence with the original vectorized distributional range maps. The resultant rasterized map of each species was consistently conservative relative to the original vectorized map, as many parts of fragmented distributions were not recorded as species presences in 1° × 1° grid cells because the area of these marginal fragments within a given cell was too small. The Latin name of each species was checked and standardized to avoid potential synonyms. In total, the gridded distribution database included range records for 424 primate species.

Climatic data with a spatial resolution of 2.5 arc min were obtained from the WorldClim database (https://worldclim.org/). The rate of climate change since the Last Glacial Maximum was estimated from the CHELSA dataset and expressed as the annual spatial displacement of climatic conditions (*106*). Land-use and land cover were collected from the study by Zhang *et al.* (*107*). In addition, NPP (*108*) and TRI data were retrieved from the Google Earth Engine Data Catalog. All environmental layers were resampled to a uniform 2.5–arc min resolution. Because phylogenetic diversity was mapped using 1° × 1° grid cells, the value of each variable within a given cell was calculated as the mean of all corresponding pixel values.

### Estimation of diversity indices and identification of hotspots

We calculated primate species richness, Faith’s phylogenetic diversity, and MDT using the “picante” package in R based on supermetric phylogenetic trees. Faith’s phylogenetic diversity is the sum of all phylogenetic branch lengths connecting species within a community (*14*). We estimated phylogenetic diversity by calculating the length of the subtree connecting each species in a grid cell to the root of the phylogenetic tree. For MDT, we defined the age of terminal species on the temporal tree as the age of their closest internal node and estimated MDT by dividing the sum of divergence times for all species in a grid cell by the total number of species in that cell.

We used three methods to identify neo-hotspots and paleo-hotspots for primate species: the *SES-MDT*, the *SES-PD*, and residual phylogenetic diversity. *SES-MDT* follows the method of Lu *et al.* (*64*), sorting divergence times from youngest to oldest across all species and generating two datasets: one comprising the 25% youngest and another the 25% oldest divergence times. For neo-hotspots, we used the dataset of species with the youngest divergence times to estimate the average divergence time per grid cell and compared it with the average divergence time from the null model. Grid cells with *SES-MDT* values below −1.96 were defined as neo-hotspots (*64*). Similarly, paleo-hotspots were calculated using the oldest divergence time dataset. Grid cells with *SES-MDT* values above 1.96 were defined as ancient hotspots and calculated using the following formula

$$\text{SES}-\text{MDT}=\frac{{\text{MDT}}_{\text{observed}}–{\text{MDT}}_{\text{random}}}{\text{SD}\left({\text{MDT}}_{\text{random}}\right)}$$

The *MDT*_*observed*_ represents the observed MDT of each grid cell based on the empirical dataset, whereas ${\text{MDT}}_{\text{random}}$ and $\text{SD}\left({\text{MDT}}_{\text{random}}\right)$ correspond to the mean and SD of the expected MDT derived from 999 randomizations under the null model. Similarly, to identify hotspot regions while controlling for variation in species richness across grid cells, the *SES-PD* was calculated by assessing whether the observed PD of a grid cell was substantially higher or lower than that expected under the null model (*109*, *110*), as follows

$$\text{SES}-\text{PD}=\frac{\left[{\text{PD}}_{\text{observed}–}\text{mean}\left({\text{PD}}_{\text{null}}\right)\right]}{\text{SD}\left({\text{PD}}_{\text{null}}\right)}$$

${\text{PD}}_{\text{observed}}$ denotes the observed phylogenetic diversity of each grid cell, while $\text{mean}\left({\text{PD}}_{\text{null}}\right)$ and $\text{SD}\left({\text{PD}}_{\text{null}}\right)$ represent the mean and SD of the expected PD values derived from 999 randomizations, respectively. This standardization effectively accounted for the influence of species richness on phylogenetic diversity. Grid cells with *SES-PD* values below −1.96 were identified as new hotspots, whereas those with *SES-PD* values above 1.96 were classified as ancient hotspots (*111*).

We also constructed a locally weighted scatter plot smoothing model to identify hotspots. In this model, phylogenetic diversity served as the response variable and species richness as the predictor variable. After model fitting, we extracted the model residuals (*112*). We evaluated the residuals for each grid cell to determine phylogenetic diversity variations after controlling for species richness (*113*, *114*). Furthermore, we identified areas with higher phylogenetic diversity than expected by the model by selecting grid cells with residual values in the top 2.5% (*83*). Grid cells with higher or lower residual values were defined as paleo-hotspots and neo-hotspots, respectively.

### Statistical analyses

To investigate the influence of environmental variables on primate species richness patterns, we examined and excluded collinearity among environmental predictors. We quantified the pairwise collinearity among the variables using the variance inflation factor (VIF). Before building the model, any predictor variable with a VIF value greater than 5 was removed to eliminate severe multicollinearity. We then constructed a negative binomial generalized linear mixed model using the following environmental variables as fixed effects: BIO3 (isothermality), BIO7 (temperature annual range), BIO12 (annual precipitation), BIO15 (precipitation seasonality), LULC, velocity of climate change, NPP, and the topographic position index. To account for potential sampling bias, we included sampling effort as a random effect in the model (*115*, *116*). A target-group approach was applied to further correct for sampling bias, treating all species collectively rather than individually, as commonly done in species distribution modeling (*99*). This approach prevents regions with limited sampling from being erroneously classified as species absent. In addition, a spatial autocovariate was included as a random effect using the spdep package (*117*) to control for spatial autocorrelation.

Model selection was performed using the dredge function in the MuMIn package to evaluate all possible subsets of the full model. Subset models with ΔAIC < 4 were retained, and the relative importance of each predictor across these models was calculated (*118*). Fixed-effect variables with relative importance > 30% were incorporated into the final model. Marginal coefficient of determination (*R*^2^) and conditional *R*^2^ were computed to represent the variance explained by fixed effects alone and by both fixed and random effects (table S5). Hierarchical partitioning using the glmm.hp package was then used to quantify the contribution of each fixed-effect variable to marginal *R*^2^ (*119*). In macroecological analyses, sampling bias is often strongly correlated with species richness and may contribute more to observed richness patterns than environmental predictors (*115*, *116*). These biases typically result from uneven sampling due to local conservation policies, the establishment of protected areas, or geographic barriers limiting accessibility (*116*, *120*). To account for this, the contributions of environmental variables were normalized relative to the sum of all fixed-effect contributions after excluding sampling bias, providing the relative influence of each variable on species richness patterns. Higher percentages indicate stronger effects of the corresponding environmental factors.

### Future climate risk assessment

To assess the potential climate risk to primate species under future climate change, the five environmental variables with the strongest influence on species richness patterns were selected: BIO3 (isothermality), BIO7 (temperature annual range), BIO12 (annual precipitation), BIO15 (precipitation seasonality), and LULC. Climate risk was quantified using the climate-change impact index proposed by Esperson-Rodriguez *et al.* (*65*). The lower boundary of climate change values within each species’ distribution range was calculated to determine whether the environmental fluctuations within a grid cell exceeded the lower limit of the species’ climatic niche. This approach quantified the extent to which each grid cell is projected to experience unsuitable climate conditions for primate persistence under future climate scenarios. The index was calculated as follows

$$\text{Risk}={\text{Grid}}_{\text{FutureClimate}\left[i\right]}–{\text{Species}}_{\text{CurrentClimate}\left[i\right]}$$

where “${\text{Species}}_{\text{CurrentClimate}\left[i\right]}$” denotes the lower limit of a species’ climate niche, defined as the 5th percentile of its climate niche values, and “${\text{Grid}}_{\text{FutureClimate}\left[i\right]}$” denotes the 5th percentile of all projected future climate values within each grid cell. A species was considered at an elevated climate risk in a grid cell when the lower boundary of future climate values exceeded its climate niche lower limit (*121*).

### Human pressures on protected areas and hotspot regions

We evaluated each species using two data types: (i) the proportion of each species’ geographic range falling within protected areas; (ii) the human activity pressure value within each species’ geographic range. Following the methodology of Rodrigues *et al.* (*122*), we overlaid each species’ distribution layer onto the protected area layer in ArcGIS 10.2 for analysis. We integrated the World Database on Protected Areas (WDPA) database (www.protectedplanet.net) with China’s most comprehensive protected area dataset (*123*) to create a relatively comprehensive global protected area dataset. This dataset is a vector layer with a minimum representable distance of ∼125 m and a resolution level meeting standard requirements for large-scale geographic analysis (*123*). To quantify the anthropogenic threat to biodiversity, we used the human footprint index (*124*), which is a raster layer with a 1-km^2^ resolution. This index is derived from an integrated calculation that combines remote sensing data and field surveys, accounting for multiple indicators such as extent of built-up area, population density, and the coverage of railways and roads (*124*). The human footprint index values range from 0, indicating no human pressure, to 1 to 2 (low pressure), 3 to 5 (moderate pressure), 6 to 11 (high pressure), and 12 to 50 (extremely high pressure) (*124*). The distribution ranges of each species were then overlaid with the human pressure layer for further analysis.

## Supplementary Materials

### This PDF file includes:

Figs. S1 to S13 Tables S1 to S5 References
