# Supplementary material for: Modeling the effects of climate risk on primates globally: New perspectives on conservation priorities
Source: Sci Adv. 2026 Aug 21;12(34):eaeg0031. doi: 10.1126/sciadv.aeg0031 (PMC13496195; doi:10.1126/sciadv.aeg0031)
Supplement: Supplementary file 1 [file sciadv.aeg0031_sm.pdf]

Science Advances
AAAS

Supplementary Materials for

Modeling the effects of climate risk on primates globally: New perspectives on conservation priorities

Yang Teng *et al.*

Corresponding author: Zuofu Xiang, xiangzf@csuft.edu.cn; Xumao Zhao, zhaoxm@lzu.edu.cn; Ming Li, lim@ioz.ac.cn

Sci. Adv. **12**, eaeg0031 (2026)
DOI: 10.1126/sciadv.aeg0031

This PDF file includes:

Figs. S1 to S13
Tables S1 to S5
References

Supplementary Figures

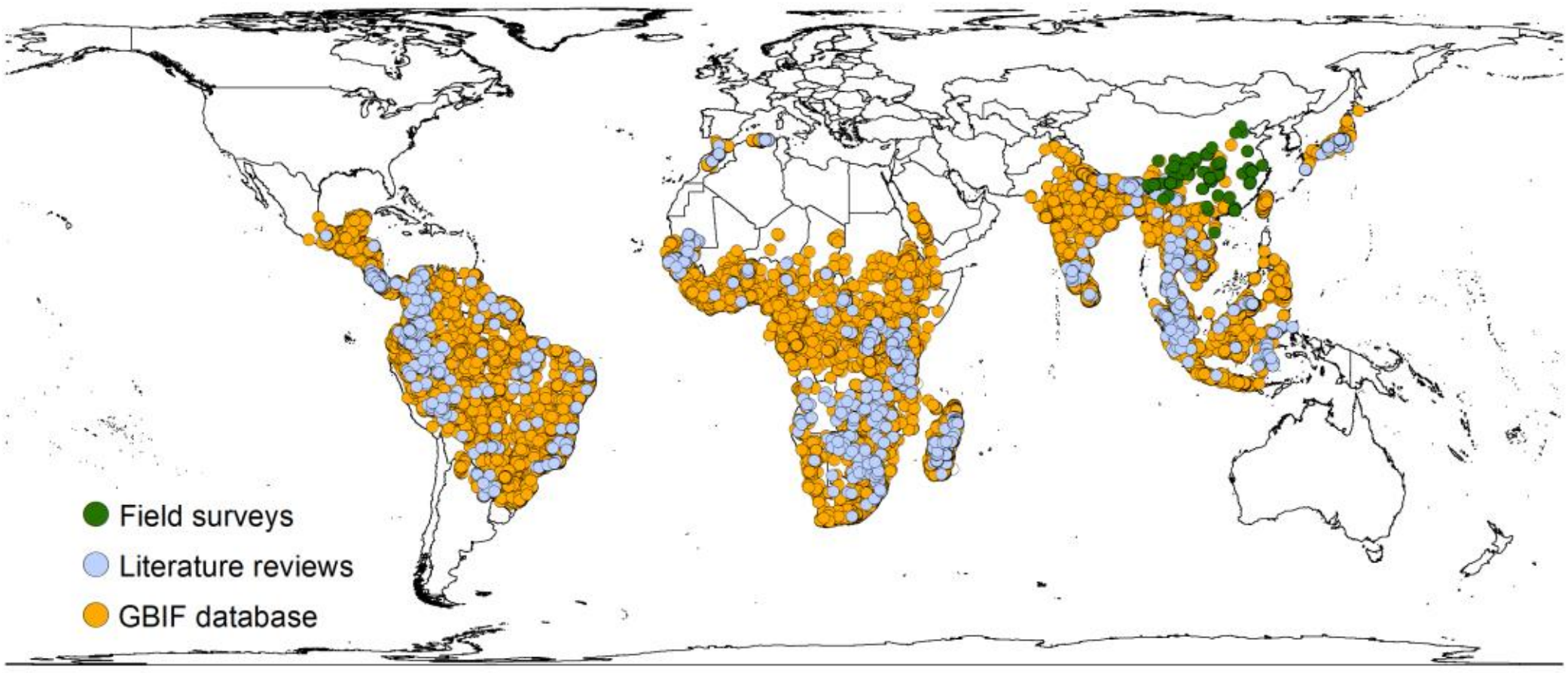

Fig. S1 Records of primate occurrence locations. Information sourced from field surveys, literature reviews, and the Global Biodiversity Information Facility (GBIF) database.

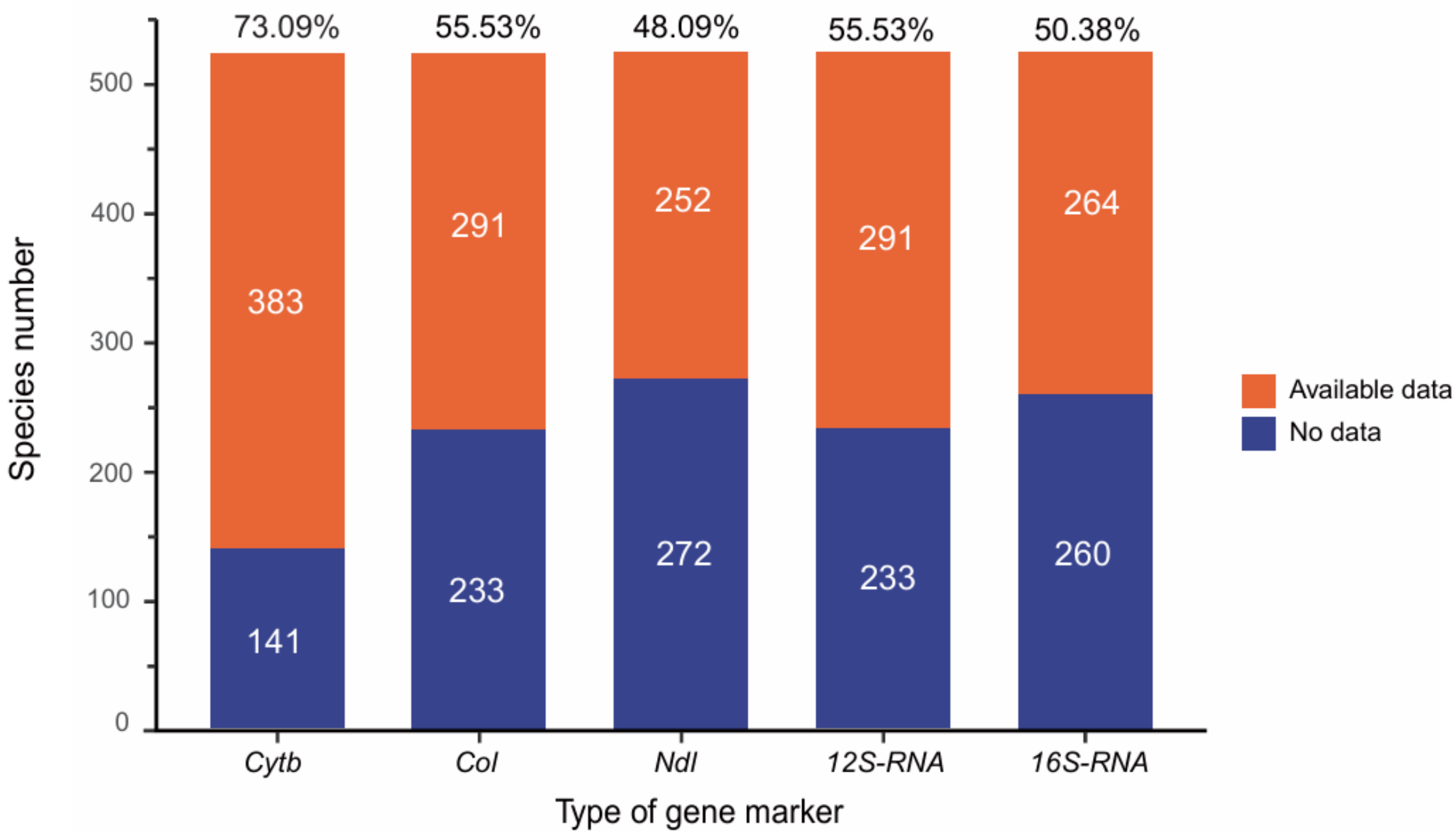

Fig. S2 Assessment status of phylogenetic diversity based on different mitochondrial genes for primates. The percent on the histogram represents the proportion of species with available phylogenetic diversity data relative to the total number of primate species (527). No data, number of species without phylogenetic diversity data; Available data, number of species with phylogenetic diversity data.

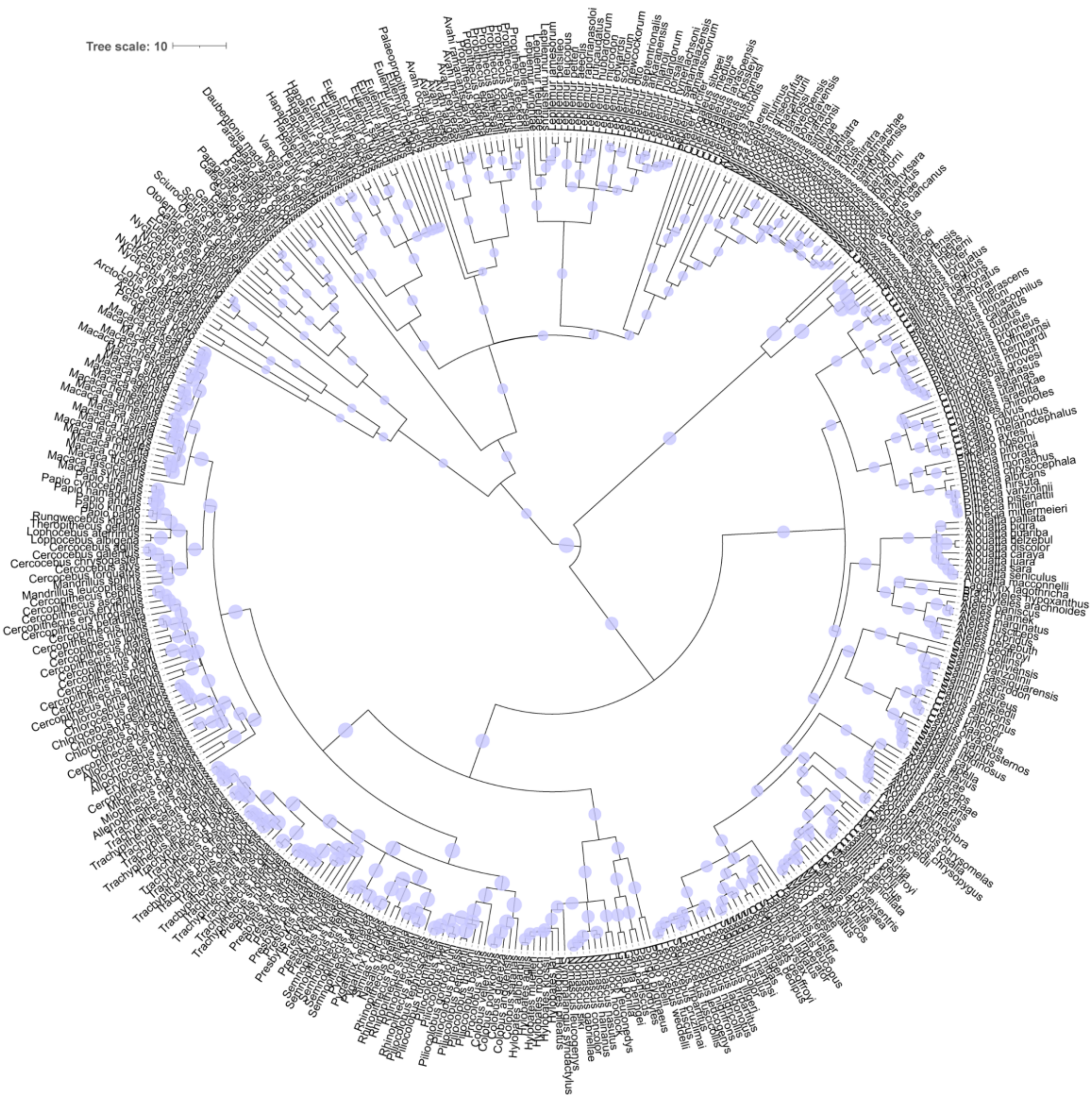

Fig. S3 Phylogenetic tree of primates. The circle indicates the size of the bootstrap value used to assess the reliability of the branch.

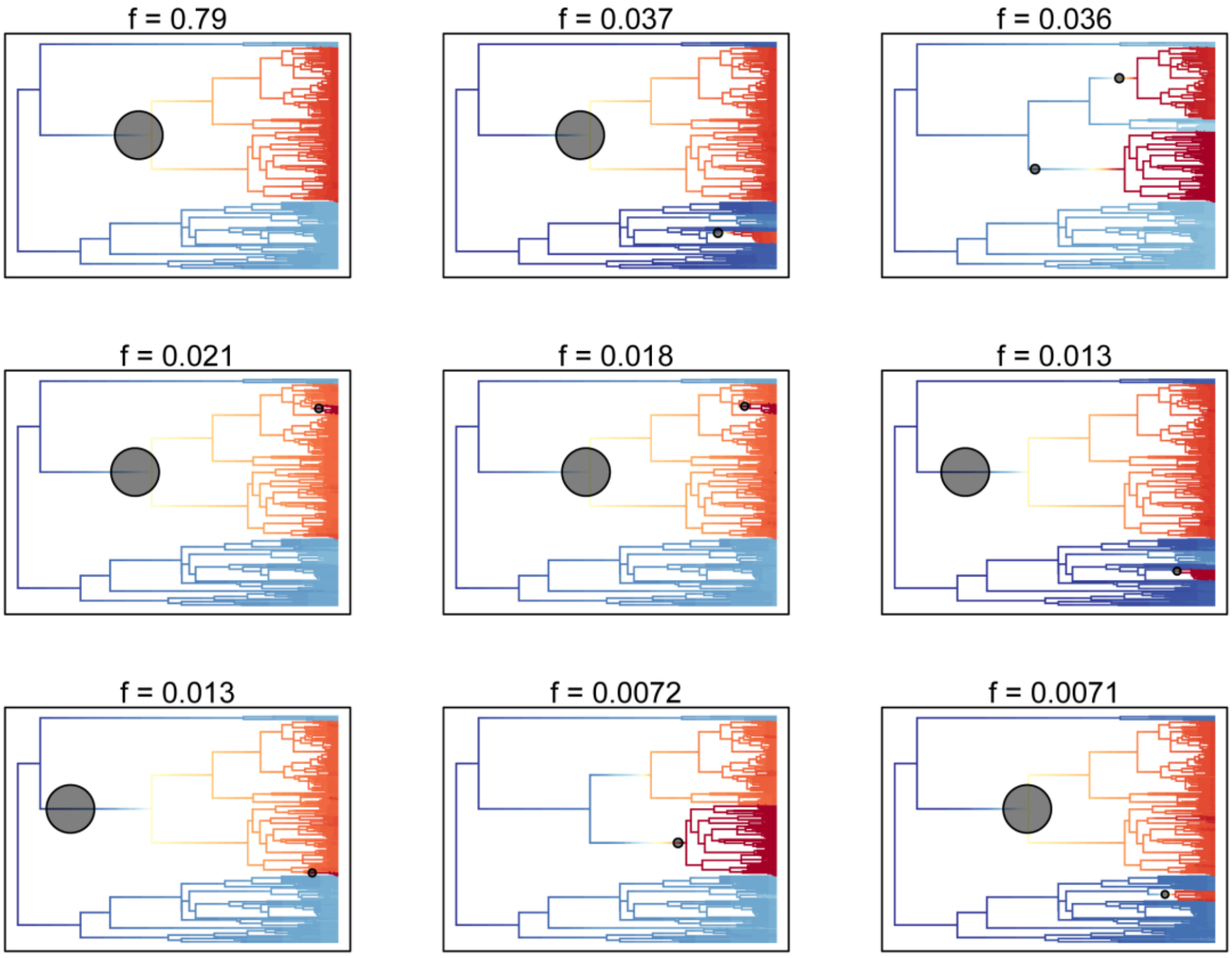

Fig. S4 Primate Phylogenetic Rate Plot. Displays the nine most likely unique transition configurations. Colors along branches indicate instantaneous speciation rates at each time point, while circle sizes are proportional to the marginal probability of the transition.

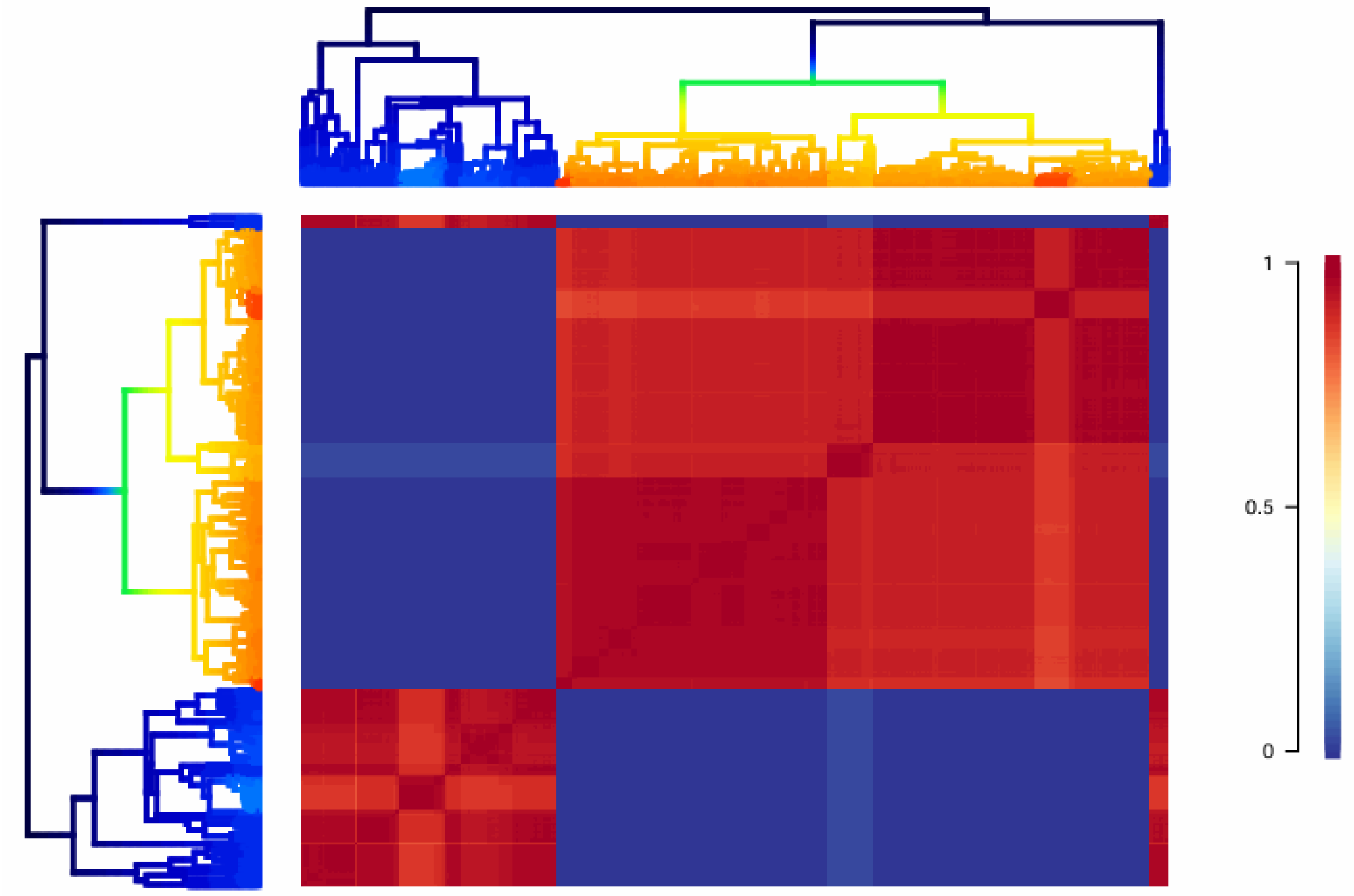

Fig. S5 Macroscopic Evolutionary Cohort Matrix for Primates. Each cell in the matrix is color-coded to represent the pairwise probability that two species share a common macroscopic evolutionary rate pattern. The primate phylogenetic tree is annotated along the left and top edges of the matrix.

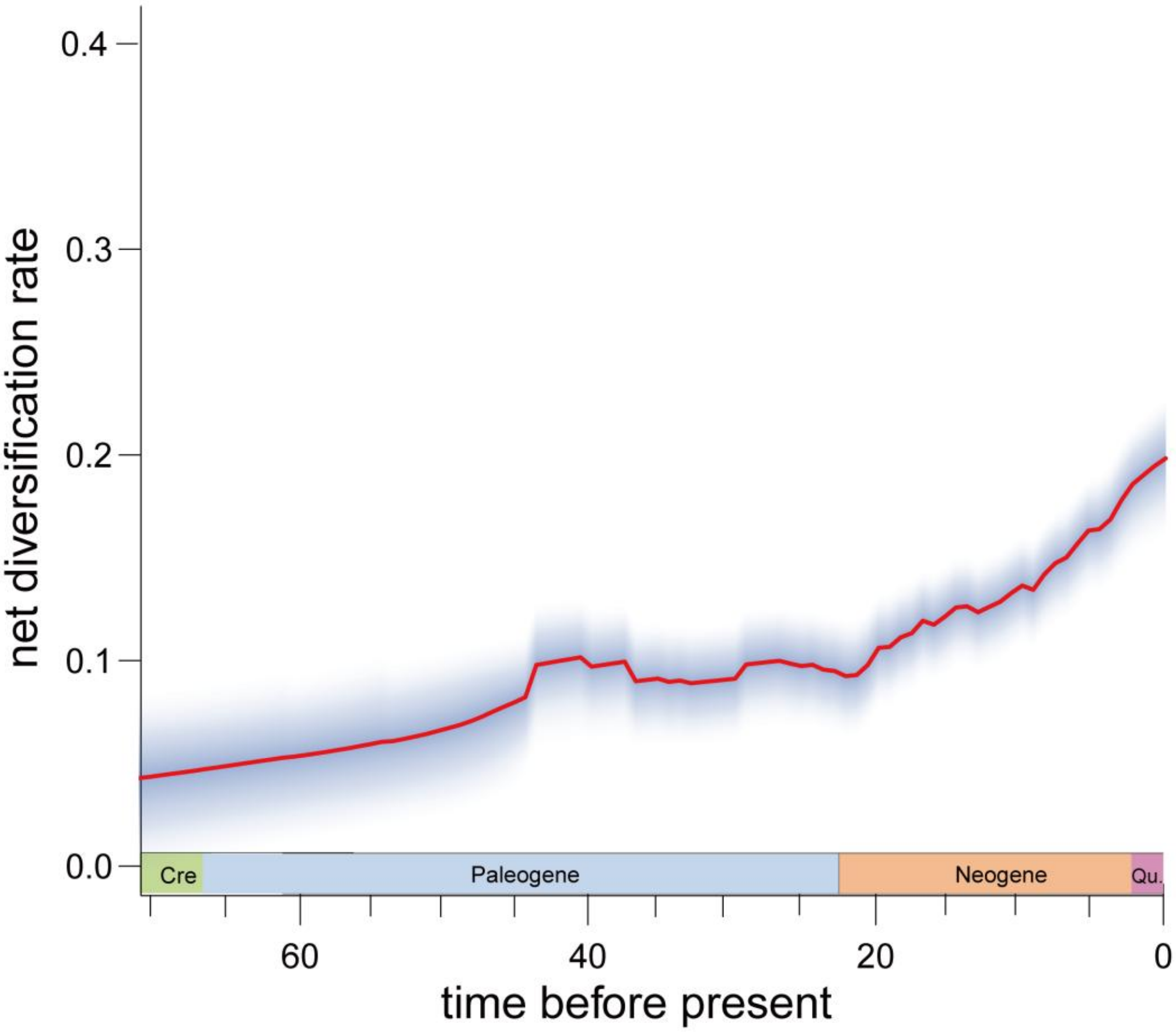

Fig. S6 Distribution of primate speciation rates over time. The purple shaded areas represent the 95% confidence intervals for the estimated speciation rates. Cre denotes the Cretaceous period, Paleogene denotes the Paleogene period, Neogene denotes the Neogene period, and Qu denotes the Quaternary period.

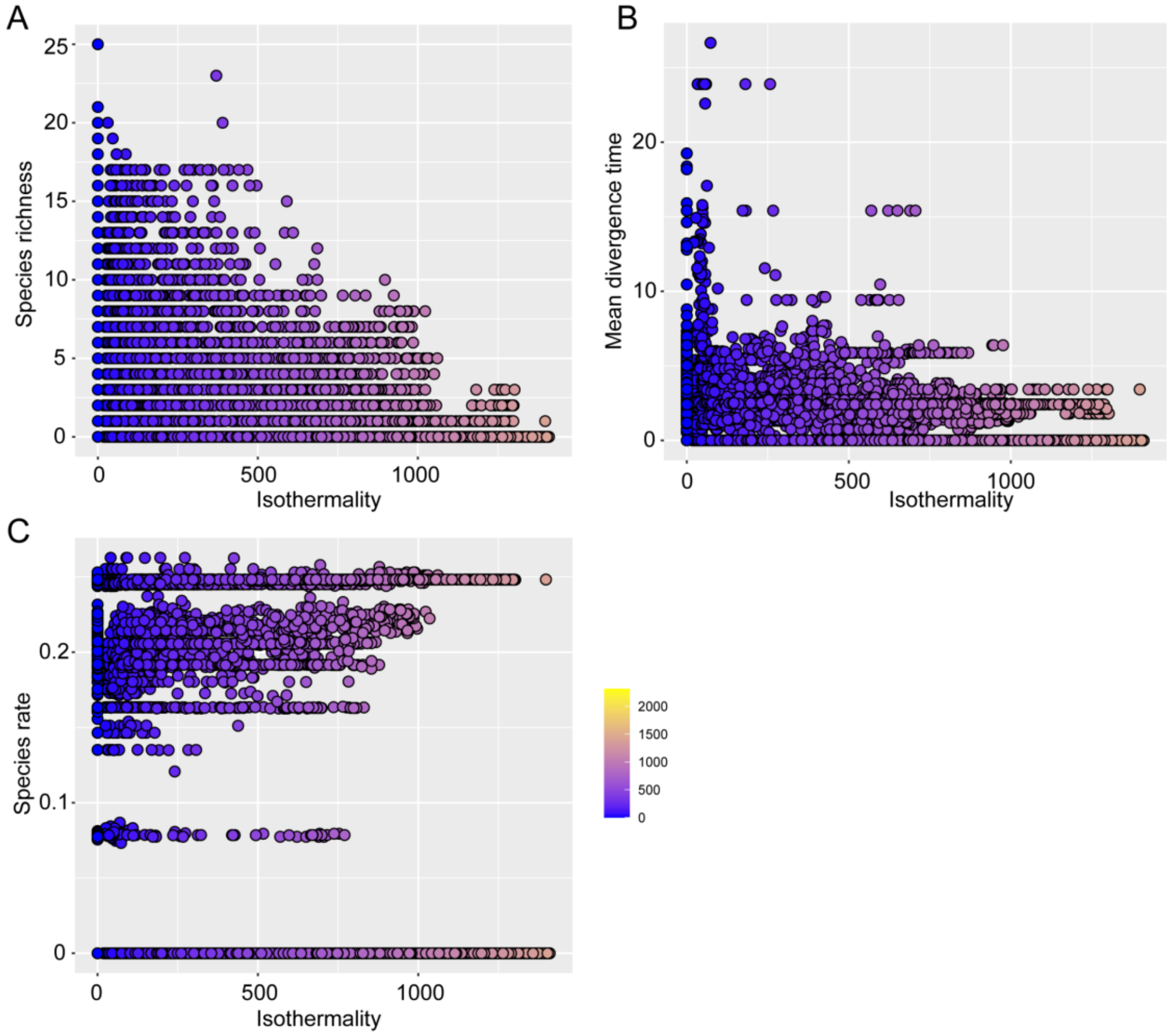

Fig. S7 Distribution relationships between isothermality and species richness (A); mean divergence time (B); and speciation rate (C). Each point represents one grid cell.

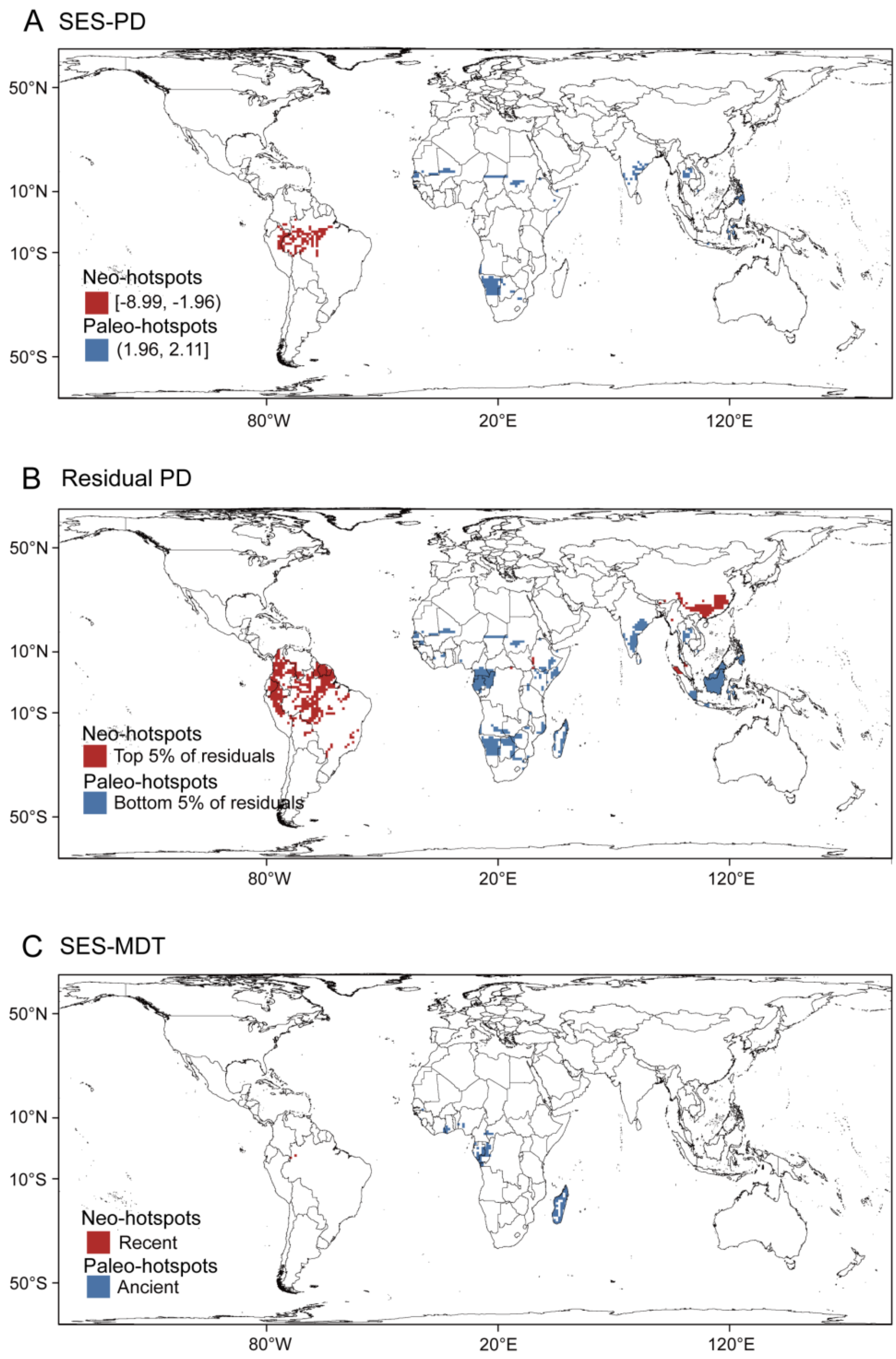

Fig. S8 Hotspots identified using three algorithms. (A) Hotspots identified by calculating standardized PD values. (B) Hotspots identified by calculating residuals from the LOESS regression model. (C) Hotspots identified by calculating the average differentiation time. Blue grid cells are identified as neo-hotspots, while red grid cells are identified as ancient or paleo-hotspots.

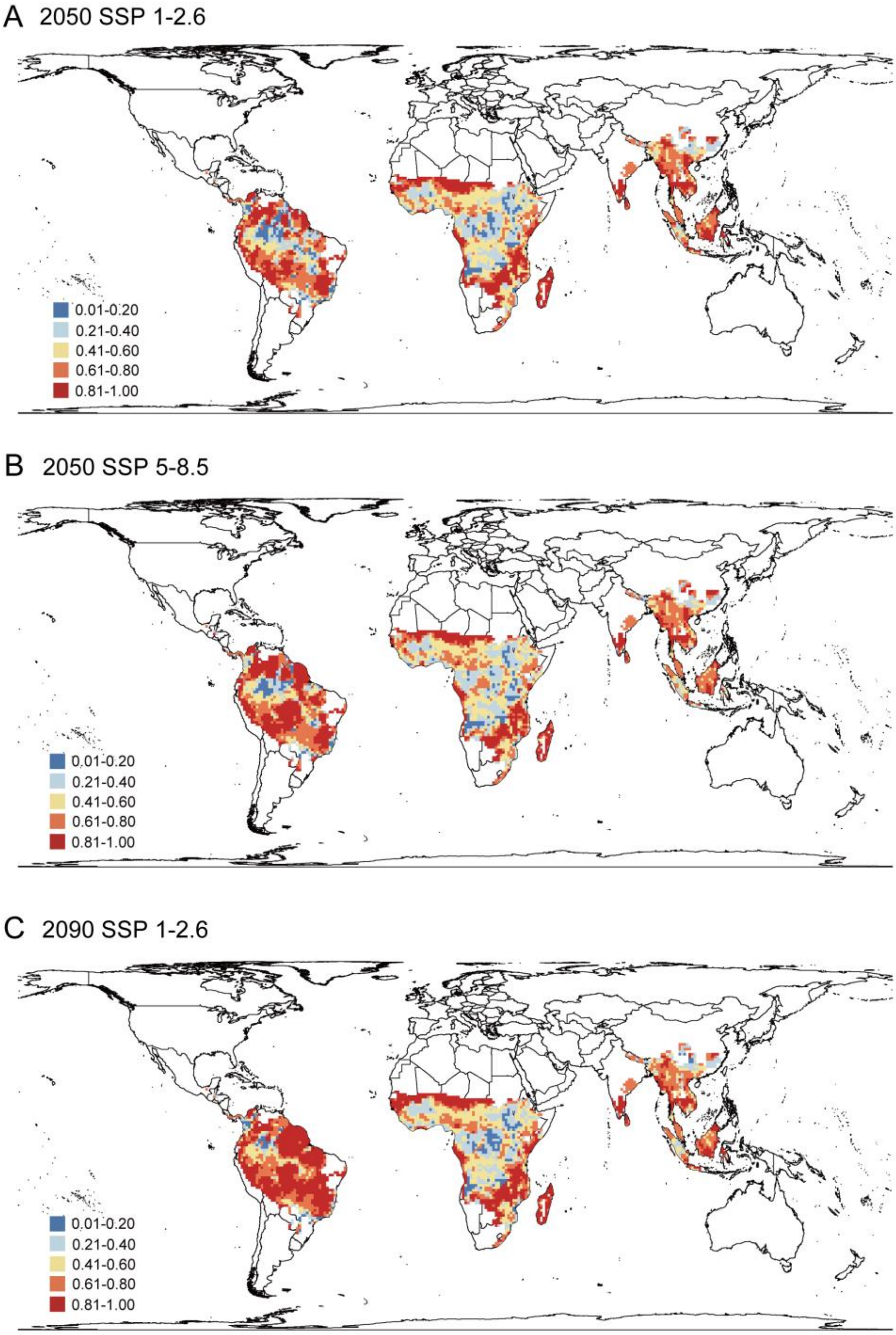

Fig. S9 Risks from future climate change and land-use changes in primates. (A) shows the risks associated with climate and land-use changes under the SSP1-2.6 scenario in 2050. (B) shows the risks associated with climate and land-use changes under the SSP5-8.5 scenario in 2050. (C) shows the risks associated with climate and land-use changes under the SSP1-2.6 scenario in 2090. The progression from blue to red indicates increasing risk severity.

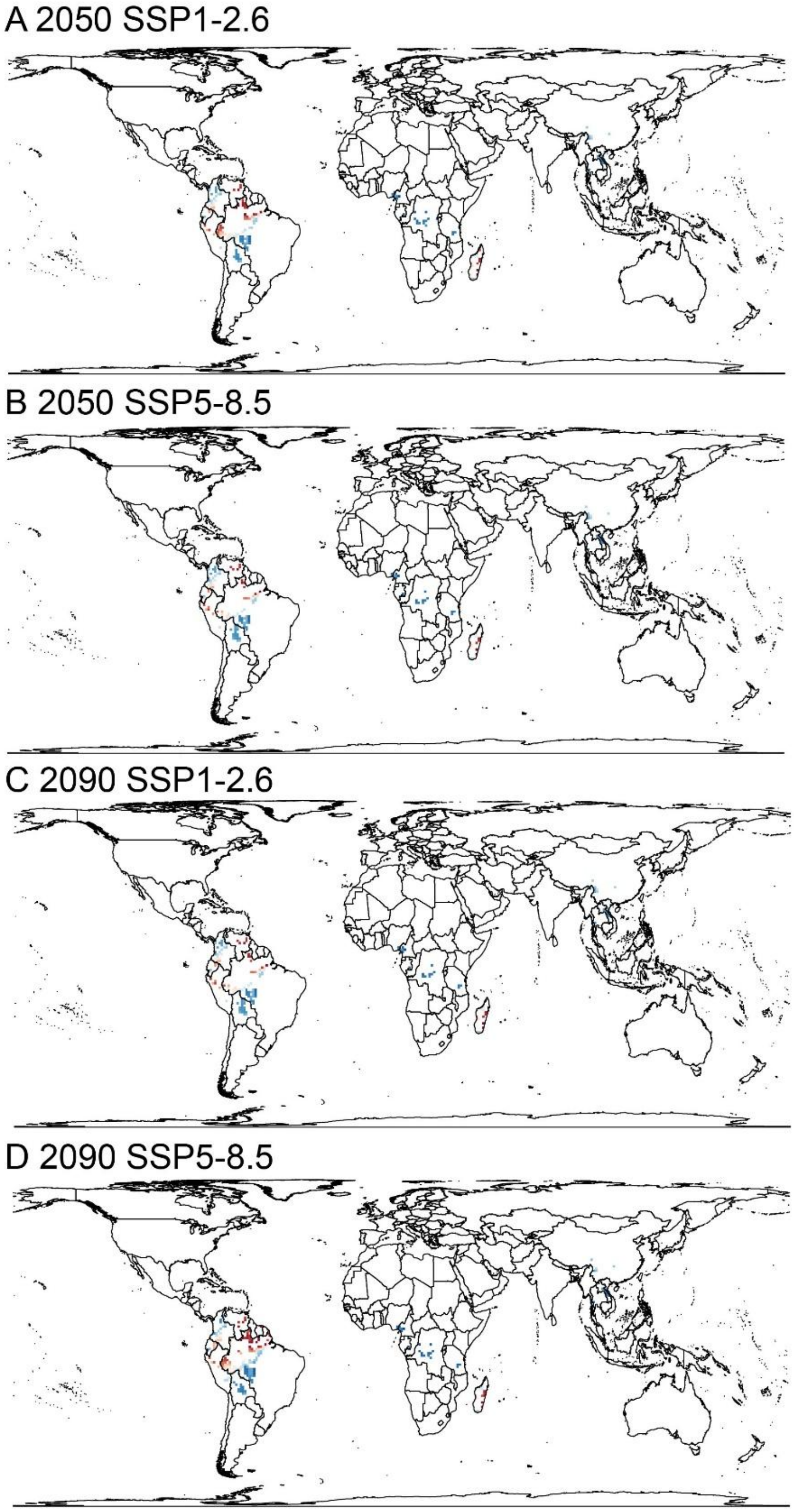

Fig. S10 Risks to primates from land-use change. (A) shows the risks associated with land-use change in 2050 under the SSP1-2.6 scenario. (B) shows the risks associated with land-use change in 2050 under the SSP5-8.5 scenario. (C) shows the risks associated with land-use changes in 2090 under the SSP1-2.6 scenario. (D) shows the risks associated with land-use changes in 2090 under the SSP5-8.5 scenario. The color gradient from blue to red indicates an increasing severity of risk.

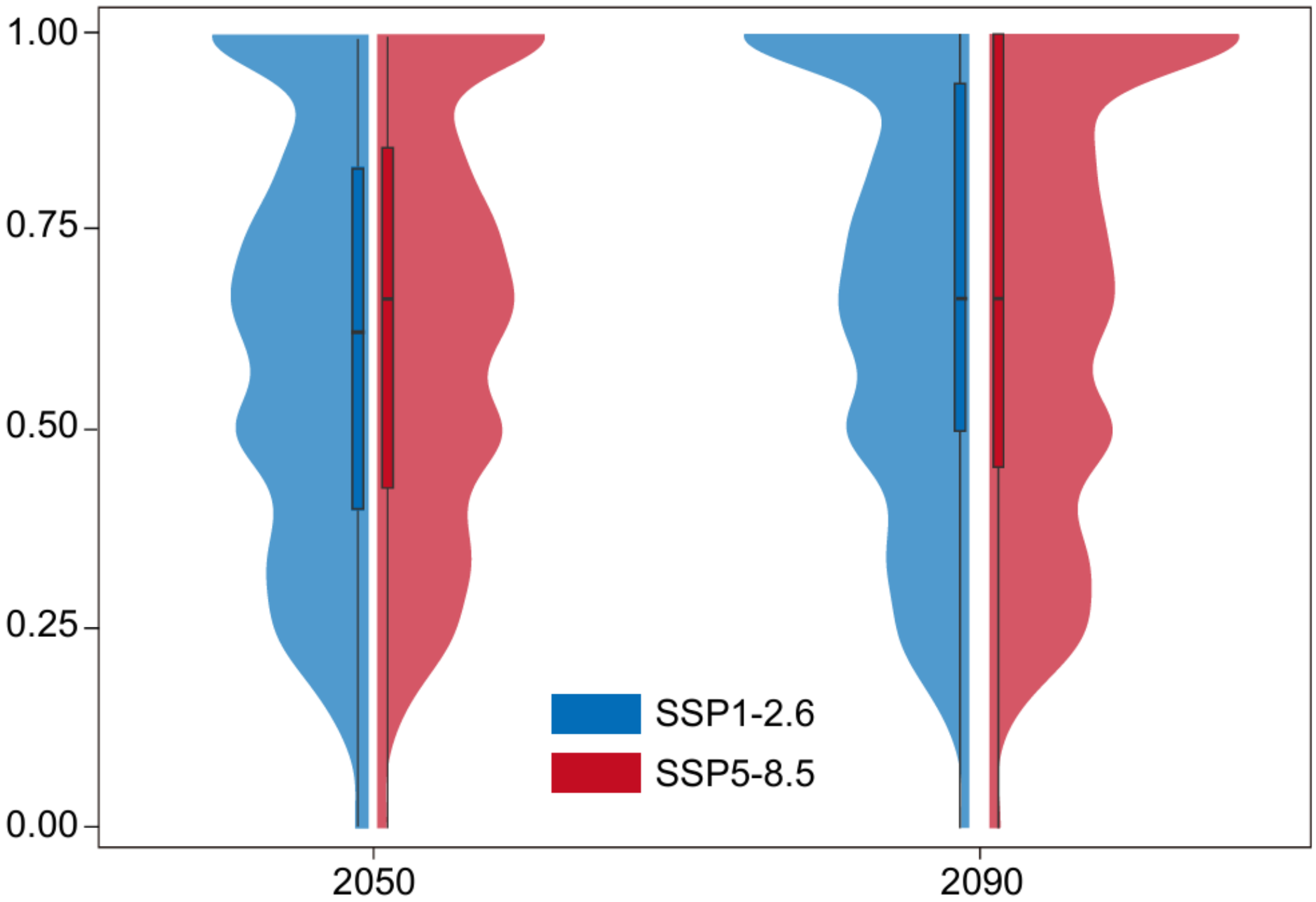

Fig. S11 Comparison of climate risk values for primates under SSP1-2.6 and SSP5-8.5 scenarios for 2050 and 2090.

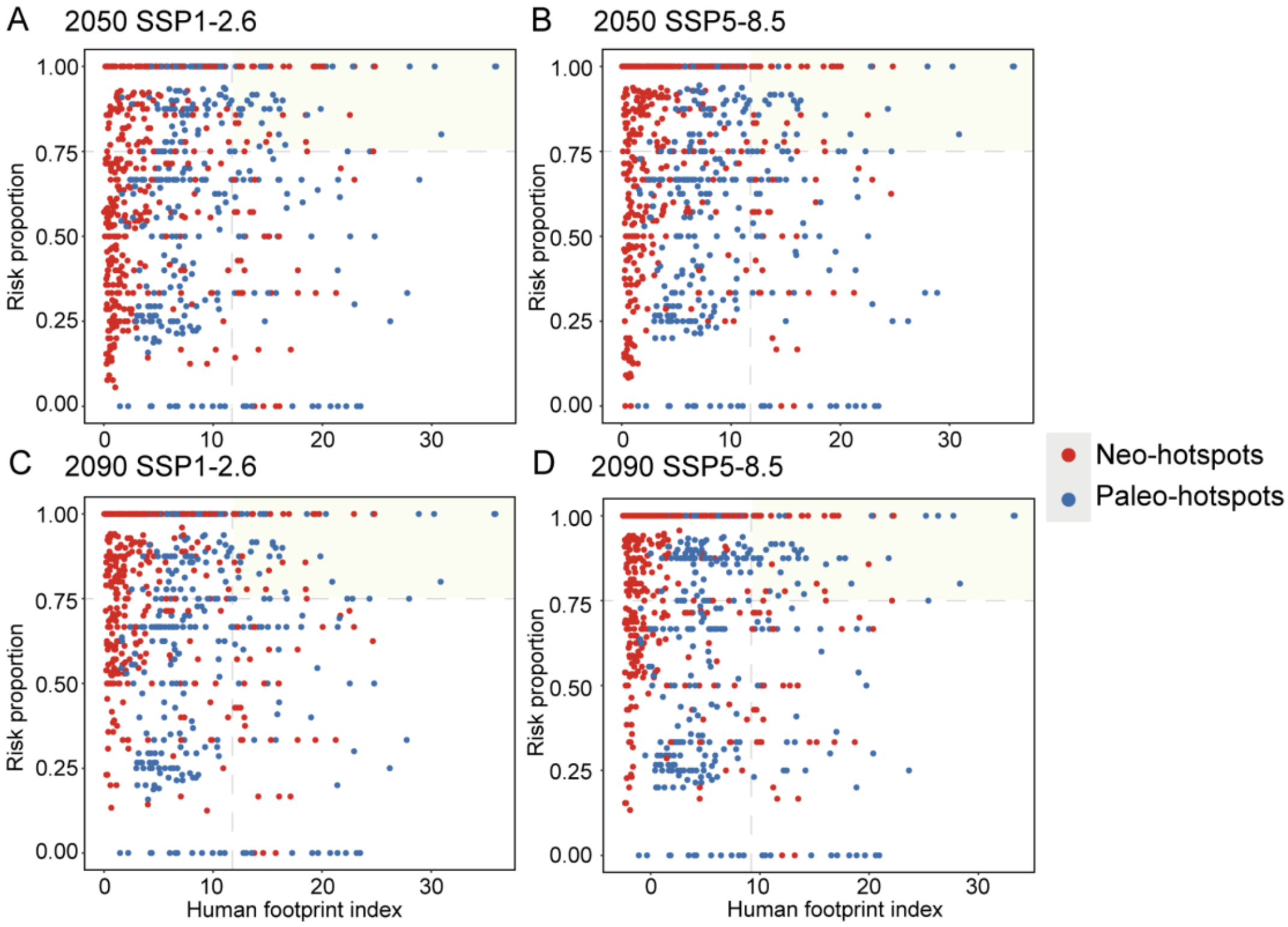

Fig.S12 Human pressure in hotspot regions. (A) Spatial overlap of the human footprint index in hotspot grid cells with the proportion of species' climate-risk under the 2050 SSP1-2.6 scenario. (B) Spatial overlap of the human footprint index in hotspot grid cells with the proportion of species' climate-risk under the 2050 SSP5-8.5 scenario. (C) Spatial overlap between the human footprint index and hotspot grid cells with the proportion of species' climate-risk under the 2090 SSP1-2.6 scenario. (D) Spatial overlap between human footprint index and hotspot grid cells with the proportion of species' climate-risk under the 2090 SSP5-8.5 scenario.

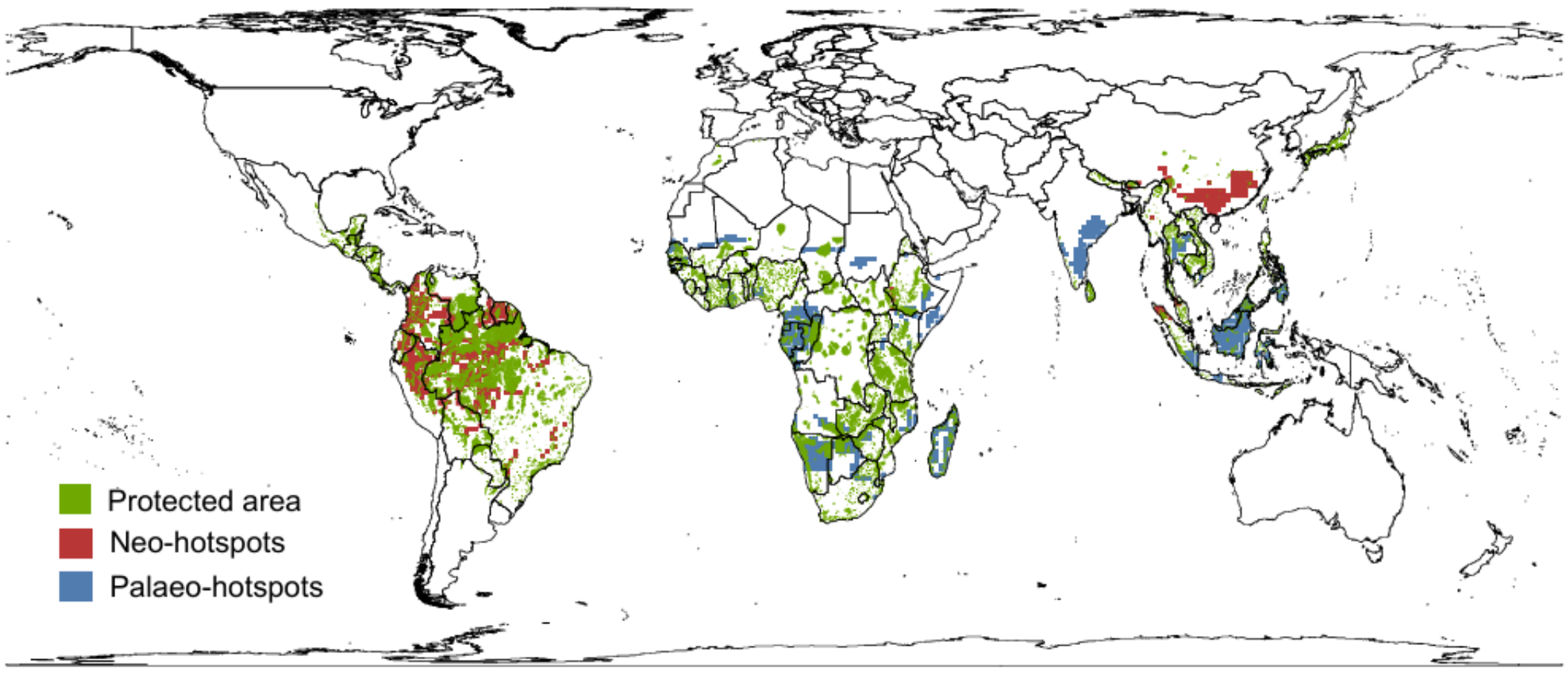

Fig.S13 Overlap of primate hotspots and protected areas. Red indicates neo-hotspots, blue denotes paleo-hotspots, and green shows protected area distribution (only protected areas within primate ranges are displayed).

Supplementary Tables

Table S1 Information of five mitochondrial genes used for phylogenetic tree construction

Species	Cytb		Co1		Nd1		12S_rRNA		16S_rRNA	
	Source	GI Number	Source	GI Number	Source	GI Number	Source	GI Number	Source	GI Number
Cheirogaleus crossleyi	mt_genome	NC_035604.1	mt_genome	NC_035604.1	mt_genome	NC_035604.1	mt_genome	NC_035604.1	mt_genome	NC_035604.1
Cheirogaleus major	mt_genome	NC_035655.1	mt_genome	NC_035655.1	mt_genome	NC_035655.1	mt_genome	NC_035655.1	mt_genome	NC_035655.1
Cheirogaleus medius	mt_genome	NC_065434.1	mt_genome	NC_065434.1	mt_genome	NC_065434.1	mt_genome	NC_065434.1	mt_genome	NC_065434.1
Cheirogaleus lavasoensis	NCBI	KM872238.1	-	-	-	-	-	-	-	-
Cheirogaleus thomasi	NCBI	KM872285.1	-	-	-	-	-	-	-	-
Cheirogaleus sibreei	mt_genome	NC_035605.1	mt_genome	NC_035605.1	mt_genome	NC_035605.1	mt_genome	NC_035605.1	mt_genome	NC_035605.1
Microcebus arnholdi	mt_genome	NC_035554.1	mt_genome	NC_035554.1	mt_genome	NC_035554.1	mt_genome	NC_035554.1	mt_genome	NC_035554.1
Microcebus berthae	mt_genome	NC_035629.1	mt_genome	NC_035629.1	mt_genome	NC_035629.1	mt_genome	NC_035629.1	mt_genome	NC_035629.1
Microcebus bongolavensis	mt_genome	NC_035559.1	mt_genome	NC_035559.1	mt_genome	NC_035559.1	mt_genome	NC_035559.1	mt_genome	NC_035559.1
Microcebus danfossi	mt_genome	NC_035600.1	mt_genome	NC_035600.1	mt_genome	NC_035600.1	mt_genome	NC_035600.1	mt_genome	NC_035600.1
Microcebus gerpi	NCBI	JQ083618.1	NCBI	OP078649.1	-	-	-	-	-	-
Microcebus griseorufus	NCBI	DQ979958.1	-	-	-	-	-	-	-	-
Microcebus jollyae	mt_genome	NC_035564.1	mt_genome	NC_035564.1	mt_genome	NC_035564.1	mt_genome	NC_035564.1	mt_genome	NC_035564.1
Microcebus lehilahytsara	NCBI	MN939645.1	-	-	-	-	-	-	-	-
Microcebus macarthurii	NCBI	EU810362.1	-	-	-	-	-	-	-	-
Microcebus mamiratra	mt_genome	NC_035601.1	mt_genome	NC_035601.1	mt_genome	NC_035601.1	mt_genome	NC_035601.1	mt_genome	NC_035601.1
Microcebus margotmarshae	mt_genome	NC_035603.1	mt_genome	NC_035603.1	mt_genome	NC_035603.1	mt_genome	NC_035603.1	mt_genome	NC_035603.1

Microcebus marohita	NCBI	GU327297.1	-	-	-	-	-	-	-	-
Microcebus tanosi	mt_genome	NC_035555.1	mt_genome	NC_035555.1	mt_genome	NC_035555.1	mt_genome	NC_035555.1	mt_genome	NC_035555.1
Microcebus murinus	mt_genome	NC_028718.1	mt_genome	NC_028718.1	mt_genome	NC_028718.1	mt_genome	NC_028718.1	mt_genome	NC_028718.1
Microcebus myoxinus	mt_genome	NC_035557.1	mt_genome	NC_035557.1	mt_genome	NC_035557.1	mt_genome	NC_035557.1	mt_genome	NC_035557.1
Microcebus ravelobensis	mt_genome	NC_035556.1	mt_genome	NC_035556.1	mt_genome	NC_035556.1	mt_genome	NC_035556.1	mt_genome	NC_035556.1
Microcebus rufus	mt_genome	NC_035562.1	mt_genome	NC_035562.1	mt_genome	NC_035562.1	mt_genome	NC_035562.1	mt_genome	NC_035562.1
Microcebus sambiranensis	mt_genome	NC_035561.1	mt_genome	NC_035561.1	mt_genome	NC_035561.1	mt_genome	NC_035561.1	mt_genome	NC_035561.1
Microcebus simmonsi	mt_genome	NC_035630.1	mt_genome	NC_035630.1	mt_genome	NC_035630.1	mt_genome	NC_035630.1	mt_genome	NC_035630.1
Microcebus tavaratra	NCBI	MF141242.1	-	-	-	-	-	-	-	-
Mirza coquereli	mt_genome	NC_035656.1	mt_genome	NC_035656.1	mt_genome	NC_035656.1	mt_genome	NC_035656.1	mt_genome	NC_035656.1
Mirza zaza	mt_genome	NC_035606.1	mt_genome	NC_035606.1	mt_genome	NC_035606.1	mt_genome	NC_035606.1	mt_genome	NC_035606.1
Allocebus trichotis	mt_genome	NC_035631.1	mt_genome	NC_035631.1	mt_genome	NC_035631.1	mt_genome	NC_035631.1	mt_genome	NC_035631.1
Phaner electromontis	mt_genome	NC_035607.1	mt_genome	NC_035607.1	mt_genome	NC_035607.1	mt_genome	NC_035607.1	mt_genome	NC_035607.1
Phaner furcifer	NCBI	AY441456.1	-	-	-	-	-	-	-	-
Phaner pallescens	mt_genome	NC_035608.1	mt_genome	NC_035608.1	mt_genome	NC_035608.1	mt_genome	NC_035608.1	mt_genome	NC_035608.1
Phaner parienti	mt_genome	NC_035609.1	mt_genome	NC_035609.1	mt_genome	NC_035609.1	mt_genome	NC_035609.1	mt_genome	NC_035609.1
Eulemur albifrons	NCBI	KF708209.1	-	-	-	-	-	-	-	-
Eulemur cinereiceps	NCBI	KF708221.1	-	-	-	-	-	-	-	-
Eulemur rufifrons	NCBI	KF708283.1	-	-	-	-	-	-	-	-
Eulemur collaris	NCBI	KF708233.1	-	-	-	-	-	-	-	-
Eulemur coronatus	NCBI	AY441448.1	-	-	-	-	-	-	-	-
Eulemur flavifrons	NCBI	KF708241.1	-	-	-	-	-	-	-	-

Eulemur fulvus	NCBI	U53576.1	-	-	-	-	-	-	-	-
Eulemur macaco	NCBI	KF708264.1	-	-	-	-	-	-	-	-
Eulemur mongoz	mt_genome	NC_010300.1	mt_genome	NC_010300.1	mt_genome	NC_010300.1	mt_genome	NC_010300.1	mt_genome	NC_010300.1
Eulemur rubriventer	NCBI	AF081052.1	-	-	-	-	-	-	-	-
Eulemur rufus	mt_genome	NC_021948.1	mt_genome	NC_021948.1	mt_genome	NC_021948.1	mt_genome	NC_021948.1	mt_genome	NC_021948.1
Eulemur sanfordi	NCBI	KF708317.1	-	-	-	-	-	-	-	-
Hapalemur aureus	NCBI	AY441446.1	-	-	-	-	NCBI	AJ430036.1	-	-
Hapalemur griseus	mt_genome	NC_021950.1	mt_genome	NC_021950.1	mt_genome	NC_021950.1	mt_genome	NC_021950.1	mt_genome	NC_021950.1
Hapalemur meridionalis	NCBI	AY441447.1	-	-	-	-	NCBI	AJ429206.1	-	-
Hapalemur occidentalis	NCBI	AJ428987.1	-	-	-	-	NCBI	AJ429215.1	-	-
Lemur catta	mt_genome	NC_059325.1	mt_genome	NC_059325.1	mt_genome	NC_059325.1	mt_genome	NC_059325.1	mt_genome	NC_059325.1
Prolemur simus	mt_genome	KX932101.1	mt_genome	KX932101.1	mt_genome	KX932101.1	mt_genome	KX932101.1	mt_genome	KX932101.1
Varecia rubra	mt_genome	NC_084385.1	mt_genome	NC_084385.1	mt_genome	NC_084385.1	mt_genome	NC_084385.1	mt_genome	NC_084385.1
Varecia variegata	mt_genome	NC_026085.1	mt_genome	NC_026085.1	mt_genome	NC_026085.1	mt_genome	NC_026085.1	mt_genome	NC_026085.1
Lepilemur aeeclis	mt_genome	NC_034730.1	mt_genome	NC_034730.1	mt_genome	NC_034730.1	mt_genome	NC_034730.1	mt_genome	NC_034730.1
Lepilemur ahmansonorum	mt_genome	NC_034733.1	mt_genome	NC_034733.1	mt_genome	NC_034733.1	mt_genome	NC_034733.1	mt_genome	NC_034733.1
Lepilemur ankaranensis	mt_genome	NC_034717.1	mt_genome	NC_034717.1	mt_genome	NC_034717.1	mt_genome	NC_034717.1	mt_genome	NC_034717.1
Lepilemur betsileo	mt_genome	NC_034726.1	mt_genome	NC_034726.1	mt_genome	NC_034726.1	mt_genome	NC_034726.1	mt_genome	NC_034726.1
Lepilemur dorsalis	mt_genome	NC_034721.1	mt_genome	NC_034721.1	mt_genome	NC_034721.1	mt_genome	NC_034721.1	mt_genome	NC_034721.1
Lepilemur edwardsi	mt_genome	NC_034720.1	mt_genome	NC_034720.1	mt_genome	NC_034720.1	mt_genome	NC_034720.1	mt_genome	NC_034720.1
Lepilemur fleuretae	mt_genome	NC_034718.1	mt_genome	NC_034718.1	mt_genome	NC_034718.1	mt_genome	NC_034718.1	mt_genome	NC_034718.1
Lepilemur grewcockorum	NCBI	MZ129031.1	-	-	-	-	-	-	-	-

Lepilemur hollandorum	mt_genome	NC_034738.1	mt_genome	NC_034738.1	mt_genome	NC_034738.1	mt_genome	NC_034738.1	mt_genome	NC_034738.1
Lepilemur hubbardorum	mt_genome	NC_014453.1	mt_genome	NC_014453.1	mt_genome	NC_014453.1	mt_genome	NC_014453.1	mt_genome	NC_014453.1
Lepilemur jamesorum	mt_genome	NC_034736.1	mt_genome	NC_034736.1	mt_genome	NC_034736.1	mt_genome	NC_034736.1	mt_genome	NC_034736.1
Lepilemur leucopus	mt_genome	NC_034719.1	mt_genome	NC_034719.1	mt_genome	NC_034719.1	mt_genome	NC_034719.1	mt_genome	NC_034719.1
Lepilemur microdon	mt_genome	NC_034739.1	mt_genome	NC_034739.1	mt_genome	NC_034739.1	mt_genome	NC_034739.1	mt_genome	NC_034739.1
Lepilemur milanoii	mt_genome	NC_034725.1	mt_genome	NC_034725.1	mt_genome	NC_034725.1	mt_genome	NC_034725.1	mt_genome	NC_034725.1
Lepilemur mustelinus	NCBI	EF686687.1	-	-	-	-	NCBI	EF588906.1	-	-
Lepilemur otto	mt_genome	NC_034724.1	mt_genome	NC_034724.1	mt_genome	NC_034724.1	mt_genome	NC_034724.1	mt_genome	NC_034724.1
Lepilemur petteri	mt_genome	NC_034723.1	mt_genome	NC_034723.1	mt_genome	NC_034723.1	mt_genome	NC_034723.1	mt_genome	NC_034723.1
Lepilemur randrianasoloi	mt_genome	NC_034722.1	mt_genome	NC_034722.1	mt_genome	NC_034722.1	mt_genome	NC_034722.1	mt_genome	NC_034722.1
Lepilemur ruficaudatus	mt_genome	NC_021953.1	mt_genome	NC_021953.1	mt_genome	NC_021953.1	mt_genome	NC_021953.1	mt_genome	NC_021953.1
Lepilemur sahamalazensis	mt_genome	NC_034734.1	mt_genome	NC_034734.1	mt_genome	NC_034734.1	mt_genome	NC_034734.1	mt_genome	NC_034734.1
Lepilemur scottorum	mt_genome	NC_034737.1	mt_genome	NC_034737.1	mt_genome	NC_034737.1	mt_genome	NC_034737.1	mt_genome	NC_034737.1
Lepilemur seali	mt_genome	NC_034731.1	mt_genome	NC_034731.1	mt_genome	NC_034731.1	mt_genome	NC_034731.1	mt_genome	NC_034731.1
Lepilemur septentrionalis	NCBI	AJ304778.1	-	-	-	-	NCBI	AJ429625.1	-	-
Lepilemur tymerlachsoni	mt_genome	NC_034735.1	mt_genome	NC_034735.1	mt_genome	NC_034735.1	mt_genome	NC_034735.1	mt_genome	NC_034735.1
Lepilemur wrightae	mt_genome	NC_034732.1	mt_genome	NC_034732.1	mt_genome	NC_034732.1	mt_genome	NC_034732.1	mt_genome	NC_034732.1
Indri indri	NCBI	AY441455.1	-	-	-	-	NCBI	AJ429618.1	-	-
Avahi cleesei	NCBI	EF103295.1	-	-	-	-	-	-	-	-
Avahi laniger	NCBI	EF103330.1	-	-	-	-	-	-	-	-
Avahi occidentalis	NCBI	AY441454.1	-	-	-	-	-	-	-	-
Propithecus candidus	mt_genome	NC_065304.1	mt_genome	NC_065304.1	mt_genome	NC_065304.1	mt_genome	NC_065304.1	mt_genome	NC_065304.1

Propithecus coquereli	mt_genome	NC_011053.1	mt_genome	NC_011053.1	mt_genome	NC_011053.1	mt_genome	NC_011053.1	mt_genome	NC_011053.1
Propithecus coronatus	mt_genome	OM650081.1	mt_genome	OM650081.1	mt_genome	OM650081.1	mt_genome	OM650081.1	mt_genome	OM650081.1
Propithecus deckenii	mt_genome	NC_065305.1	mt_genome	NC_065305.1	mt_genome	NC_065305.1	mt_genome	NC_065305.1	mt_genome	NC_065305.1
Propithecus diadema	mt_genome	NC_026084.1	mt_genome	NC_026084.1	mt_genome	NC_026084.1	mt_genome	NC_026084.1	mt_genome	NC_026084.1
Propithecus edwardsi	mt_genome	NC_026086.1	mt_genome	NC_026086.1	mt_genome	NC_026086.1	mt_genome	NC_026086.1	mt_genome	NC_026086.1
Propithecus perrieri	mt_genome	OM649962.1	mt_genome	OM649962.1	mt_genome	OM649962.1	mt_genome	OM649962.1	mt_genome	OM649962.1
Propithecus tattersalli	mt_genome	NC_027740.1	mt_genome	NC_027740.1	mt_genome	NC_027740.1	mt_genome	NC_027740.1	mt_genome	NC_027740.1
Propithecus verreauxi	mt_genome	NC_028210.1	mt_genome	NC_028210.1	mt_genome	NC_028210.1	mt_genome	NC_028210.1	mt_genome	NC_028210.1
Palaeopropithecus ingens	mt_genome	NC_026090.1	mt_genome	NC_026090.1	mt_genome	NC_026090.1	mt_genome	NC_026090.1	mt_genome	NC_026090.1
Daubentonia madagascariensis	mt_genome	AB371085.1	mt_genome	AB371085.1	mt_genome	AB371085.1	mt_genome	AB371085.1	mt_genome	AB371085.1
Arctocebus calabarensis	NCBI	AY441474.1	-	-	-	-	-	-	-	-
Arctocebus aureus	-	-	NCBI	OK184259.1	-	-	NCBI	OK184035.1	NCBI	OK183792.1
Perodicticus potto	mt_genome	AB371095.1	mt_genome	AB371095.1	mt_genome	AB371095.1	mt_genome	AB371095.1	mt_genome	AB371095.1
Perodicticus edwardsi	mt_genome	KC757407.1	mt_genome	KC757407.1	mt_genome	KC757407.1	mt_genome	KC757407.1	mt_genome	KC757407.1
Perodicticus ibeanus	NCBI	KP410651.1	-	-	-	-	-	-	-	-
Loris tardigradus	mt_genome	AB371094.1	mt_genome	AB371094.1	mt_genome	AB371094.1	mt_genome	AB371094.1	mt_genome	AB371094.1
Loris lydekkerianus	mt_genome	NC_021955.1	mt_genome	NC_021955.1	mt_genome	NC_021955.1	mt_genome	NC_021955.1	mt_genome	NC_021955.1
Nycticebus bengalensis	NCBI	OP902170.1	NCBI	OQ466052.1	-	-	-	-	-	-
Nycticebus borneanus	-	-	NCBI	OQ518093.1	-	-	-	-	-	-
Nycticebus coucang	mt_genome	CM050178.1	mt_genome	CM050178.1	mt_genome	CM050178.1	mt_genome	CM050178.1	mt_genome	CM050178.1
Nycticebus javanicus	NCBI	KP410658.1	NCBI	OQ518084.1	-	-	-	-	NCBI	GQ253663.1

Nycticebus hilleri	-	-	NCBI	OQ518054.1	-	-	-	-	-	-
Nycticebus kayan	-	-	NCBI	OQ518100.1	-	-	-	-	-	-
Nycticebus menagensis	NCBI	KP410632.1	NCBI	OQ518102.1	-	-	NCBI	GQ253661.1	-	-
Nycticebus pygmaeus	mt_genome	NC_033381.1	mt_genome	NC_033381.1	mt_genome	NC_033381.1	mt_genome	NC_033381.1	mt_genome	NC_033381.1
Euoticus elegantulus	NCBI	AY441469.1	-	-	-	-	NCBI	DQ073480.1	-	-
Galago gallarum	NCBI	AF212970.1	-	-	-	-	NCBI	AF212950.1	-	-
Galago matschiei	NCBI	AF271409.1	-	-	-	-	-	-	-	-
Galago moholi	mt_genome	NC_021949.1	mt_genome	NC_021949.1	mt_genome	NC_021949.1	mt_genome	NC_021949.1	mt_genome	NC_021949.1
Galago senegalensis	mt_genome	AB371092.1	mt_genome	AB371092.1	mt_genome	AB371092.1	mt_genome	AB371092.1	mt_genome	AB371092.1
Galagoides demidoff	NCBI	KP410668.1	NCBI	KJ192819.1	-	-	-	-	-	-
Galagoides thomasi	NCBI	KP410671.1	-	-	-	-	-	-	-	-
Paragalago zanzibaricus	NCBI	MK034951.1	-	-	-	-	NCBI	DQ073497.1	NCBI	AF212954.1
Paragalago cocos	NCBI	MK034932.1	-	-	-	-	-	-	-	-
Paragalago granti	NCBI	KP410657.1	-	-	-	-	-	-	-	-
Sciurocheirus alleni	NCBI	KP410660.1	-	-	-	-	NCBI	DQ073523.1	-	-
Sciurocheirus gabonensis	NCBI	KP410603.1	-	-	-	-	-	-	-	-
Otolemur crassicaudatus	mt_genome	KJ434961.1	mt_genome	KJ434961.1	mt_genome	KJ434961.1	mt_genome	KJ434961.1	mt_genome	KJ434961.1
Otolemur garnettii	NCBI	OP329367.1	NCBI	AY671787.1	-	-	NCBI	DQ073507.1	-	-
Carlito syrichta	mt_genome	NC_012774.1	mt_genome	NC_012774.1	mt_genome	NC_012774.1	mt_genome	NC_012774.1	mt_genome	NC_012774.1
Cephalopachus bancanus	mt_genome	AF348159.1	mt_genome	AF348159.1	mt_genome	AF348159.1	mt_genome	AF348159.1	mt_genome	AF348159.1
Tarsius dentatus	mt_genome	NC_024052.1	mt_genome	NC_024052.1	mt_genome	NC_024052.1	mt_genome	NC_024052.1	mt_genome	NC_024052.1
Tarsius fuscus	NCBI	KR337090.1	-	-	-	-	-	-	-	-

Tarsius lariang	NCBI	FJ214337.1	-	-	-	-	-	-	-	-
Tarsius sangirensis	-	-	-	-	-	-	NCBI	HM470206.1	-	-
Tarsius tarsier	-	-	-	-	-	-	NCBI	HM470226.1	-	-
Tarsius wallacei	mt_genome	KC977311.1	mt_genome	KC977311.1	mt_genome	KC977311.1	mt_genome	KC977311.1	mt_genome	KC977311.1
Callimico goeldii	mt_genome	NC_024628.1	mt_genome	NC_024628.1	mt_genome	NC_024628.1	mt_genome	NC_024628.1	mt_genome	NC_024628.1
Callibella humilis	mt_genome	NC_064164.1	mt_genome	NC_064164.1	mt_genome	NC_064164.1	mt_genome	NC_064164.1	mt_genome	NC_064164.1
Callithrix aurita	mt_genome	NC_050682.1	mt_genome	NC_050682.1	mt_genome	NC_050682.1	mt_genome	NC_050682.1	mt_genome	NC_050682.1
Callithrix geoffroyi	mt_genome	NC_021941.1	mt_genome	NC_021941.1	mt_genome	NC_021941.1	mt_genome	NC_021941.1	mt_genome	NC_021941.1
Callithrix jacchus	mt_genome	NC_025586.1	mt_genome	NC_025586.1	mt_genome	NC_025586.1	mt_genome	NC_025586.1	mt_genome	NC_025586.1
Callithrix kuhlii	mt_genome	NC_027658.1	mt_genome	NC_027658.1	mt_genome	NC_027658.1	mt_genome	NC_027658.1	mt_genome	NC_027658.1
Callithrix penicillata	mt_genome	NC_030788.1	mt_genome	NC_030788.1	mt_genome	NC_030788.1	mt_genome	NC_030788.1	mt_genome	NC_030788.1
Cebuella pygmaea	mt_genome	MZ747454.1	mt_genome	MZ747454.1	mt_genome	MZ747454.1	mt_genome	MZ747454.1	mt_genome	MZ747454.1
Cebuella niveiventris	NCBI	OQ993333.1	-	-	-	-	-	-	-	-
Mico chrysoleucos	NCBI	KR528411.1	-	-	-	-	-	-	-	-
Mico argentatus	mt_genome	NC_064175.1	mt_genome	NC_064175.1	mt_genome	NC_064175.1	mt_genome	NC_064175.1	mt_genome	NC_064175.1
Mico marcai	NCBI	MW736156.1	-	-	-	-	-	-	-	-
Mico mauesi	NCBI	AF245051.1	-	-	-	-	-	-	-	-
Mico humeralifer	mt_genome	NC_064174.1	mt_genome	NC_064174.1	mt_genome	NC_064174.1	mt_genome	NC_064174.1	mt_genome	NC_064174.1
Mico emiliae	NCBI	OP573226.1	-	-	-	-	-	-	-	-
Mico melanurus	NCBI	AF245050.1	-	-	-	-	-	-	-	-
Leontopithecus chrysomelas	NCBI	KR528398.1	-	-	-	-	-	-	-	-
Leontopithecus chrysopygus	mt_genome	NC_037878.1	mt_genome	NC_037878.1	mt_genome	NC_037878.1	mt_genome	NC_037878.1	mt_genome	NC_037878.1

Leontopithecus rosalia	mt_genome	MZ262294.1	mt_genome	MZ262294.1	mt_genome	MZ262294.1	mt_genome	MZ262294.1	mt_genome	MZ262294.1
Saguinus bicolor	mt_genome	NC_064176.1	mt_genome	NC_064176.1	mt_genome	NC_064176.1	mt_genome	NC_064176.1	mt_genome	NC_064176.1
Saguinus martinsi	NCBI	MW736159.1	-	-	-	-	-	-	NCBI	EU497277.1
Saguinus midas	mt_genome	NC_064179.1	mt_genome	NC_064179.1	mt_genome	NC_064179.1	mt_genome	NC_064179.1	mt_genome	NC_064179.1
Saguinus mystax	NCBI	OP573227.1	NCBI	MZ364429.1	-	-	-	-	NCBI	MZ364473.1
Saguinus niger	-	-	NCBI	OM728633.1	-	-	-	-	NCBI	EU497255.1
Leontocebus tripartitus	NCBI	HM368077.1	-	-	-	-	-	-	-	-
Leontocebus leucogenys	NCBI	HM368074.1	-	-	-	-	-	-	-	-
Leontocebus cruzlimai	NCBI	KP182176.1	-	-	-	-	-	-	-	-
Leontocebus nigricollis	mt_genome	NC_064171.1	mt_genome	NC_064171.1	mt_genome	NC_064171.1	mt_genome	NC_064171.1	mt_genome	NC_064171.1
Leontocebus fuscus	-	-	NCBI	OM728612.1	-	-	-	-	-	-
Leontocebus lagonotus	NCBI	HM368059.1	-	-	-	-	-	-	-	-
Leontocebus illigeri	NCBI	HM368069.1	-	-	-	-	-	-	-	-
Leontocebus nigrifrons	NCBI	HM368056.1	-	-	-	-	-	-	-	-
Leontocebus fuscicollis	mt_genome	NC_064181.1	mt_genome	NC_064181.1	mt_genome	NC_064181.1	mt_genome	NC_064181.1	mt_genome	NC_064181.1
Leontocebus weddelli	-	-	NCBI	OM728637.1	-	-	-	-	-	-
Cebus albifrons	mt_genome	OR689234.1	mt_genome	OR689234.1	mt_genome	OR689234.1	mt_genome	OR689234.1	mt_genome	OR689234.1
Cebus capucinus	NCBI	FJ529110.1	-	-	-	-	NCBI	JN400573.1	-	-
Cebus castaneus	mt_genome	NC_087896.1	mt_genome	NC_087896.1	mt_genome	NC_087896.1	mt_genome	NC_087896.1	mt_genome	NC_087896.1
Cebus kaapori	mt_genome	NC_087895.1	mt_genome	NC_087895.1	mt_genome	NC_087895.1	mt_genome	NC_087895.1	mt_genome	NC_087895.1
Cebus imitator	mt_genome	NC_087897.1	mt_genome	NC_087897.1	mt_genome	NC_087897.1	mt_genome	NC_087897.1	mt_genome	NC_087897.1
Cebus unicolor	mt_genome	PP454508.1	mt_genome	PP454508.1	mt_genome	PP454508.1	mt_genome	PP454508.1	mt_genome	PP454508.1

Cebus olivaceus	mt_genome	NC_064165.1	mt_genome	NC_064165.1	mt_genome	NC_064165.1	mt_genome	NC_064165.1	mt_genome	NC_064165.1
Cebus versicolor	NCBI	KY173250.1	-	-	-	-	-	-	-	-
Sapajus apella	mt_genome	PP454529.1	mt_genome	PP454529.1	mt_genome	PP454529.1	mt_genome	PP454529.1	mt_genome	PP454529.1
Sapajus cay	mt_genome	NC_087898.1	mt_genome	NC_087898.1	mt_genome	NC_087898.1	mt_genome	NC_087898.1	mt_genome	NC_087898.1
Sapajus flavius	mt_genome	NC_050883.1	mt_genome	NC_050883.1	mt_genome	NC_050883.1	mt_genome	NC_050883.1	mt_genome	NC_050883.1
Sapajus libidinosus	mt_genome	NC_087899.1	mt_genome	NC_087899.1	mt_genome	NC_087899.1	mt_genome	NC_087899.1	mt_genome	NC_087899.1
Sapajus nigritus	NCBI	OR606449.1	-	-	-	-	-	-	-	-
Sapajus robustus	mt_genome	PP454556.1	mt_genome	PP454556.1	mt_genome	PP454556.1	mt_genome	PP454556.1	mt_genome	PP454556.1
Sapajus xanthosternos	mt_genome	PP454561.1	mt_genome	PP454561.1	mt_genome	PP454561.1	mt_genome	PP454561.1	mt_genome	PP454561.1
Saimiri ustus	mt_genome	NC_064168.1	mt_genome	NC_064168.1	mt_genome	NC_064168.1	mt_genome	NC_064168.1	mt_genome	NC_064168.1
Saimiri oerstedii	mt_genome	NC_064173.1	mt_genome	NC_064173.1	mt_genome	NC_064173.1	mt_genome	NC_064173.1	mt_genome	NC_064173.1
Saimiri cassiquiarensis	mt_genome	NC_064182.1	mt_genome	NC_064182.1	mt_genome	NC_064182.1	mt_genome	NC_064182.1	mt_genome	NC_064182.1
Saimiri boliviensis	mt_genome	NC_021966.1	mt_genome	NC_021966.1	mt_genome	NC_021966.1	mt_genome	NC_021966.1	mt_genome	NC_021966.1
Saimiri collinsi	NCBI	KJ128122.1	-	-	-	-	-	-	-	-
Saimiri sciureus	mt_genome	AB371091.1	mt_genome	AB371091.1	mt_genome	AB371091.1	mt_genome	AB371091.1	mt_genome	AB371091.1
Aotus azarae	NCBI	DQ098866.1	-	-	-	-	-	-	-	-
Aotus brumbacki	-	-	NCBI	DQ321669.1	-	-	-	-	-	-
Aotus griseimembra	NCBI	MT488672.1	NCBI	MT492170.1	-	-	-	-	-	-
Aotus lemurinus	mt_genome	NC_019799.1	mt_genome	NC_019799.1	mt_genome	NC_019799.1	mt_genome	NC_019799.1	mt_genome	NC_019799.1
Aotus nancymaae	mt_genome	NC_018116.1	mt_genome	NC_018116.1	mt_genome	NC_018116.1	mt_genome	NC_018116.1	mt_genome	NC_018116.1
Aotus nigriceps	NCBI	MW736164.1	-	-	-	-	-	-	-	-
Aotus trivirgatus	mt_genome	NC_064150.1	mt_genome	NC_064150.1	mt_genome	NC_064150.1	mt_genome	NC_064150.1	mt_genome	NC_064150.1

Aotus vociferans	mt_genome	NC_064163.1	mt_genome	NC_064163.1	mt_genome	NC_064163.1	mt_genome	NC_064163.1	mt_genome	NC_064163.1
Pithecia irrorata	NCBI	OL800284.1	-	-	-	-	-	-	-	-
Pithecia mittermeieri	NCBI	NC_064160.1	-	-	-	-	-	-	-	-
Pithecia pissinattii	mt_genome	OM328981.1	mt_genome	OM328981.1	mt_genome	OM328981.1	mt_genome	OM328981.1	mt_genome	OM328981.1
Pithecia hirsuta	NCBI	NC_064159.1	-	-	-	-	-	-	-	-
Pithecia monachus	NCBI	FJ531668.1	-	-	-	-	-	-	-	-
Pithecia albicans	mt_genome	NC_064161.1	mt_genome	NC_064161.1	mt_genome	NC_064161.1	mt_genome	NC_064161.1	mt_genome	NC_064161.1
Pithecia pithecia	NCBI	KR902426.1	NCBI	AY549424.1	-	-	-	-	-	-
Pithecia vanzolinii	mt_genome	NC_064151.1	mt_genome	NC_064151.1	mt_genome	NC_064151.1	mt_genome	NC_064151.1	mt_genome	NC_064151.1
Chiropotes albinasus	mt_genome	NC_021946.1	mt_genome	NC_021946.1	mt_genome	NC_021946.1	mt_genome	NC_021946.1	mt_genome	NC_021946.1
Chiropotes chiropotes	mt_genome	NC_064157.1	mt_genome	NC_064157.1	mt_genome	NC_064157.1	mt_genome	NC_064157.1	mt_genome	NC_064157.1
Chiropotes sagulatus	NCBI	KM370857.1	-	-	-	-	-	-	-	-
Chiropotes satanas	-	-	-	-	-	-	NCBI	AF069966.1	-	-
Chiropotes utahickae	NCBI	AY226185.1	-	-	-	-	-	-	-	-
Cacajao calvus	mt_genome	OR689233.1	mt_genome	OR689233.1	mt_genome	OR689233.1	mt_genome	OR689233.1	mt_genome	OR689233.1
Cacajao melanocephalus	mt_genome	OM328908.1	mt_genome	OM328908.1	mt_genome	OM328908.1	mt_genome	OM328908.1	mt_genome	OM328908.1
Cacajao ucayalii	NCBI	ON337702.1	-	-	-	-	-	-	-	-
Cacajao novaesi	mt_genome	OM328885.1	mt_genome	OM328885.1	mt_genome	OM328885.1	mt_genome	OM328885.1	mt_genome	OM328885.1
Cacajao rubicundus	NCBI	ON337694.1	-	-	-	-	-	-	-	-
Cacajao ayresi	mt_genome	NC_064153.1	mt_genome	NC_064153.1	mt_genome	NC_064153.1	mt_genome	NC_064153.1	mt_genome	NC_064153.1
Cacajao hosomi	mt_genome	NC_064149.1	mt_genome	NC_064149.1	mt_genome	NC_064149.1	mt_genome	NC_064149.1	mt_genome	NC_064149.1

Callicebus nigrifrons	NCBI	MN998492.1	NCBI	OR041501.1	-	-	-	-	-	-
Callicebus personatus	NCBI	MN998491.1	NCBI	MN998566.1	-	-	-	-	-	-
Cheracebus lugens	mt_genome	OM328973.1	mt_genome	OM328973.1	mt_genome	OM328973.1	mt_genome	OM328973.1	mt_genome	OM328973.1
Cheracebus lucifer	mt_genome	NC_064199.1	mt_genome	NC_064199.1	mt_genome	NC_064199.1	mt_genome	NC_064199.1	mt_genome	NC_064199.1
Cheracebus regulus	mt_genome	NC_064197.1	mt_genome	NC_064197.1	mt_genome	NC_064197.1	mt_genome	NC_064197.1	mt_genome	NC_064197.1
Cheracebus torquatus	mt_genome	NC_064198.1	mt_genome	NC_064198.1	mt_genome	NC_064198.1	mt_genome	NC_064198.1	mt_genome	NC_064198.1
Xenothrix mcgregori	mt_genome	MK073942.1	mt_genome	MK073942.1	mt_genome	MK073942.1	mt_genome	MK073942.1	mt_genome	MK073942.1
Plecturocebus dubius	mt_genome	NC_064204.1	mt_genome	NC_064204.1	mt_genome	NC_064204.1	mt_genome	NC_064204.1	mt_genome	NC_064204.1
Plecturocebus donacophilus	mt_genome	NC_019801.1	mt_genome	NC_019801.1	mt_genome	NC_019801.1	mt_genome	NC_019801.1	mt_genome	NC_019801.1
Plecturocebus caligatus	mt_genome	NC_064202.1	mt_genome	NC_064202.1	mt_genome	NC_064202.1	mt_genome	NC_064202.1	mt_genome	NC_064202.1
Plecturocebus discolor	NCBI	KX353788.1	-	-	-	-	-	-	-	-
Plecutrocebus moloch	NCBI	AF524887.1	-	-	-	-	NCBI	AF069980.1	NCBI	AB107212.1
Plecturocebus cinerascens	mt_genome	NC_064203.1	mt_genome	NC_064203.1	mt_genome	NC_064203.1	mt_genome	NC_064203.1	mt_genome	NC_064203.1
Plecturocebus brunneus	mt_genome	NC_064201.1	mt_genome	NC_064201.1	mt_genome	NC_064201.1	mt_genome	NC_064201.1	mt_genome	NC_064201.1
Plecturocebus hoffmannsi	mt_genome	NC_064206.1	mt_genome	NC_064206.1	mt_genome	NC_064206.1	mt_genome	NC_064206.1	mt_genome	NC_064206.1
Plecturocebus bernhardi	mt_genome	NC_064200.1	mt_genome	NC_064200.1	mt_genome	NC_064200.1	mt_genome	NC_064200.1	mt_genome	NC_064200.1
Plecturocebus caquetensis	NCBI	KX353781.1	-	-	-	-	-	-	-	-
Plecturocebus miltoni	mt_genome	NC_064207.1	mt_genome	NC_064207.1	mt_genome	NC_064207.1	mt_genome	NC_064207.1	mt_genome	NC_064207.1
Plecturocebus cupreus	mt_genome	OM328991.1	mt_genome	OM328991.1	mt_genome	OM328991.1	mt_genome	OM328991.1	mt_genome	OM328991.1
Plecturocebus toppini	NCBI	PV391750.1	NCBI	PV403249.1						
Plecturocebus grovesi	mt_genome	NC_064205.1	mt_genome	NC_064205.1	mt_genome	NC_064205.1	mt_genome	NC_064205.1	mt_genome	NC_064205.1
Alouatta belzebul	NCBI	MW958322.1	-	-	-	-	-	-	-	-

Alouatta caraya	NCBI	DQ350637.1	-	-	-	-	-	-	-	-
Alouatta discolor	mt_genome	PV032482.1	mt_genome	PV032482.1	mt_genome	PV032482.1	mt_genome	PV032482.1	mt_genome	PV032482.1
Alouatta juara	mt_genome	NC_064196.1	mt_genome	NC_064196.1	mt_genome	NC_064196.1	mt_genome	NC_064196.1	mt_genome	NC_064196.1
Alouatta guariba	NCBI	PQ278423.1	-	-	-	-	-	-	-	-
Alouatta macconnelli	mt_genome	PV032513.1	mt_genome	PV032513.1	mt_genome	PV032513.1	mt_genome	PV032513.1	mt_genome	PV032513.1
Alouatta puruennsis	mt_genome	OM329058.1	mt_genome	OM329058.1	mt_genome	OM329058.1	mt_genome	OM329058.1	mt_genome	OM329058.1
Alouatta nigerrima	mt_genome	OM329043.1	mt_genome	OM329043.1	mt_genome	OM329043.1	mt_genome	OM329043.1	mt_genome	OM329043.1
Alouatta palliata	-	-	NCBI	MZ636589.1	-	-	-	-	-	-
Alouatta pigra	NCBI	PQ815841.1	-	-	-	-	-	-	-	-
Alouatta sara	NCBI	OR544373.1	NCBI	PV403554.1	-	-	-	-	-	-
Alouatta seniculus	mt_genome	NC_027825.1	mt_genome	NC_027825.1	mt_genome	NC_027825.1	mt_genome	NC_027825.1	mt_genome	NC_027825.1
Alouatta ululata	mt_genome	PV032492.1	mt_genome	PV032492.1	mt_genome	PV032492.1	mt_genome	PV032492.1	mt_genome	PV032492.1
Ateles belzebuth	mt_genome	NC_019800.1	mt_genome	NC_019800.1	mt_genome	NC_019800.1	mt_genome	NC_019800.1	mt_genome	NC_019800.1
Ateles chamek	mt_genome	NC_064190.1	mt_genome	NC_064190.1	mt_genome	NC_064190.1	mt_genome	NC_064190.1	mt_genome	NC_064190.1
Ateles fusciceps	-	-	NCBI	LC799535.1	-	-	NCBI	AY012132.1	NCBI	AY011164.1
Ateles geoffroyi	NCBI	KM347892.1	NCBI	AB016730.1	-	-	-	-	NCBI	AB116026.1
Ateles hybridus	-	-	NCBI	LC799542.1	-	-	-	-	-	-
Ateles marginatus	mt_genome	NC_064192.1	mt_genome	NC_064192.1	mt_genome	NC_064192.1	mt_genome	NC_064192.1	mt_genome	NC_064192.1
Ateles paniscus	mt_genome	NC_064191.1	mt_genome	NC_064191.1	mt_genome	NC_064191.1	mt_genome	NC_064191.1	mt_genome	NC_064191.1
Brachyteles arachnoides	NCBI	AY065906.1	-	-	-	-	-	-	-	-
Lagothrix lagothricha	NCBI	OM328878.1	-	-	-	-	-	-	-	-
Allenopithecus nigroviridis	mt_genome	NC_023965.1	mt_genome	NC_023965.1	mt_genome	NC_023965.1	mt_genome	NC_023965.1	mt_genome	NC_023965.1

Miopithecus ogouensis	-	-	-	-	-	-	NCBI	AF164877.1	-	-
Miopithecus talapoin	-	-	NCBI	EU185729.1	-	-	NCBI	L35205.1	-	-
Allochrocebus lhoesti	mt_genome	NC_023962.1	mt_genome	NC_023962.1	mt_genome	NC_023962.1	mt_genome	NC_023962.1	mt_genome	NC_023962.1
Allochrocebus preussi	-	-	NCBI	OK184330.1	-	-	NCBI	OK184109.1	NCBI	OK183843.1
Allochrocebus solatus	-	-	-	-	-	-	NCBI	AF394270.1	NCBI	AF435506.1
Erythrocebus patas	NCBI	OP089546.1	NCBI	MT109110.1	-	-	NCBI	KJ192497.1	-	-
Chlorocebus aethiops	mt_genome	NC_007009.1	mt_genome	NC_007009.1	mt_genome	NC_007009.1	mt_genome	NC_007009.1	mt_genome	NC_007009.1
Chlorocebus cynosuros	mt_genome	NC_024933.1	mt_genome	NC_024933.1	mt_genome	NC_024933.1	mt_genome	NC_024933.1	mt_genome	NC_024933.1
Chlorocebus dryas	NCBI	MN450181.1	-	-	-	-	-	-	-	-
Chlorocebus djamdjamensis	mt_genome	NC_034277.1	mt_genome	NC_034277.1	mt_genome	NC_034277.1	mt_genome	NC_034277.1	mt_genome	NC_034277.1
Chlorocebus pygerythrus	mt_genome	NC_009747.1	mt_genome	NC_009747.1	mt_genome	NC_009747.1	mt_genome	NC_009747.1	mt_genome	NC_009747.1
Chlorocebus sabaeus	NCBI	PV329797.1	-	-	-	-	-	-	-	-
Chlorocebus tantalus	mt_genome	NC_009748.1	mt_genome	NC_009748.1	mt_genome	NC_009748.1	mt_genome	NC_009748.1	mt_genome	NC_009748.1
Cercopithecus ascanius	NCBI	MT024315.1	NCBI	MT020851.1	-	-	NCBI	L35202.1	-	-
Cercopithecus campbelli	NCBI	PV329725.1	-	-	-	-	-	-	-	-
Cercopithecus cephus	NCBI	OR362291.1	NCBI	OK184333.1	-	-	NCBI	L35191.1	-	-
Cercopithecus denti	mt_genome	JQ256988.1	mt_genome	JQ256988.1	mt_genome	JQ256988.1	mt_genome	JQ256988.1	mt_genome	JQ256988.1
Cercopithecus diana	mt_genome	NC_023963.1	mt_genome	NC_023963.1	mt_genome	NC_023963.1	mt_genome	NC_023963.1	mt_genome	NC_023963.1
Cercopithecus lowei	-	-	-	-	-	-	NCBI	AY665619.1	-	-
Cercopithecus erythrogaster	NCBI	HQ450787.1	NCBI	KJ192905.1	-	-	NCBI	KJ192638.1	NCBI	KJ193200.1
Cercopithecus erythrotis	-	-	NCBI	OK184347.1	-	-	NCBI	OK184124.1	-	-
Cercopithecus hamlyni	-	-	NCBI	EU179512.1	-	-	NCBI	AF164881.1	NCBI	AY665624.1

Cercopithecus mitis	mt_genome	NC_023961.1	mt_genome	NC_023961.1	mt_genome	NC_023961.1	mt_genome	NC_023961.1	mt_genome	NC_023961.1
Cercopithecus mona	NCBI	OR167542.1	-	-	-	-	NCBI	L35198.1	NCBI	AF435504.1
Cercopithecus neglectus	mt_genome	NC_056341.1	mt_genome	NC_056341.1	mt_genome	NC_056341.1	mt_genome	NC_056341.1	mt_genome	NC_056341.1
Cercopithecus nictitans	-	-	NCBI	OK184309.1	-	-	NCBI	L35199.1	NCBI	OK183814.1
Cercopithecus petaurista	NCBI	OP089537.1	NCBI	KJ192960.1	-	-	NCBI	KJ192694.1	-	-
Cercopithecus pogonias	-	-	NCBI	GQ144570.1	-	-	-	-	-	-
Cercopithecus roloway	mt_genome	JQ256923.1	mt_genome	JQ256923.1	mt_genome	JQ256923.1	mt_genome	JQ256923.1	mt_genome	JQ256923.1
Cercopithecus wolfi	-	-	NCBI	AY972679.1	-	-	-	-	-	-
Macaca arctoides	mt_genome	NC_025201.1	mt_genome	NC_025201.1	mt_genome	NC_025201.1	mt_genome	NC_025201.1	mt_genome	NC_025201.1
Macaca assamensis	mt_genome	NC_023795.1	mt_genome	NC_023795.1	mt_genome	NC_023795.1	mt_genome	NC_023795.1	mt_genome	NC_023795.1
Macaca cyclopis	mt_genome	NC_027449.1	mt_genome	NC_027449.1	mt_genome	NC_027449.1	mt_genome	NC_027449.1	mt_genome	NC_027449.1
Macaca fascicularis	mt_genome	MF893979.1	mt_genome	MF893979.1	mt_genome	MF893979.1	mt_genome	MF893979.1	mt_genome	MF893979.1
Macaca fuscata	mt_genome	NC_025513.1	mt_genome	NC_025513.1	mt_genome	NC_025513.1	mt_genome	NC_025513.1	mt_genome	NC_025513.1
Macaca hecki	NCBI	HM071118.1	-	-	-	-	NCBI	AY633524.1	-	-
Macaca leucogenys	mt_genome	NC_031156.1	mt_genome	NC_031156.1	mt_genome	NC_031156.1	mt_genome	NC_031156.1	mt_genome	NC_031156.1
Macaca leonina	mt_genome	NC_027604.1	mt_genome	NC_027604.1	mt_genome	NC_027604.1	mt_genome	NC_027604.1	mt_genome	NC_027604.1
Macaca maura	NCBI	HM071115.1	-	-	-	-	NCBI	AY633535.1	-	-
Macaca mulatta	mt_genome	OQ199664.1	mt_genome	OQ199664.1	mt_genome	OQ199664.1	mt_genome	OQ199664.1	mt_genome	OQ199664.1
Macaca munzala	mt_genome	PQ740951.1	mt_genome	PQ740951.1	mt_genome	PQ740951.1	mt_genome	PQ740951.1	mt_genome	PQ740951.1
Macaca nemestrina	mt_genome	NC_026976.1	mt_genome	NC_026976.1	mt_genome	NC_026976.1	mt_genome	NC_026976.1	mt_genome	NC_026976.1
Macaca nigra	mt_genome	NC_026120.1	mt_genome	NC_026120.1	mt_genome	NC_026120.1	mt_genome	NC_026120.1	mt_genome	NC_026120.1
Macaca nigrescens	NCBI	AF350385.1	-	-	-	-	NCBI	AY633526.1	-	-

Macaca ochreata	NCBI	HM071124.1	-	-	-	-	NCBI	AY206545.1	-	-
Macaca pagensis	NCBI	AY151129.1	-	-	-	-	-	-	-	-
Macaca radiata	NCBI	KM583996.1	-	-	-	-	-	-	-	-
Macaca siberu	NCBI	AY151113.1	-	-	-	-	NCBI	DQ355497.1	NCBI	DQ355497.1
Macaca silenus	mt_genome	KM679363.1	mt_genome	KM679363.1	mt_genome	KM679363.1	mt_genome	KM679363.1	mt_genome	KM679363.1
Macaca sinica	mt_genome	NC_037437.1	mt_genome	NC_037437.1	mt_genome	NC_037437.1	mt_genome	NC_037437.1	mt_genome	NC_037437.1
Macaca sylvanus	mt_genome	NC_002764.1	mt_genome	NC_002764.1	mt_genome	NC_002764.1	mt_genome	NC_002764.1	mt_genome	NC_002764.1
Macaca thibetana	mt_genome	NC_011519.1	mt_genome	NC_011519.1	mt_genome	NC_011519.1	mt_genome	NC_011519.1	mt_genome	NC_011519.1
Macaca tonkeana	mt_genome	NC_025222.1	mt_genome	NC_025222.1	mt_genome	NC_025222.1	mt_genome	NC_025222.1	mt_genome	NC_025222.1
Lophocebus albigena	NCBI	KJ193380.1	NCBI	KJ192835.1	-	-	NCBI	KJ192553.1	NCBI	KJ193115.1
Lophocebus aterrimus	mt_genome	NC_021954.1	mt_genome	NC_021954.1	mt_genome	NC_021954.1	mt_genome	NC_021954.1	mt_genome	NC_021954.1
Rungwecebus kipunji	-	-	NCBI	GU068069.1	-	-	NCBI	GU068062.1	-	-
Papio anubis	mt_genome	MG787545.1	mt_genome	MG787545.1	mt_genome	MG787545.1	mt_genome	MG787545.1	mt_genome	MG787545.1
Papio cynocephalus	mt_genome	NC_020007.2	mt_genome	NC_020007.2	mt_genome	NC_020007.2	mt_genome	NC_020007.2	mt_genome	NC_020007.2
Papio hamadryas	mt_genome	JX946201.2	mt_genome	JX946201.2	mt_genome	JX946201.2	mt_genome	JX946201.2	mt_genome	JX946201.2
Papio kindae	mt_genome	NC_020008.2	mt_genome	NC_020008.2	mt_genome	NC_020008.2	mt_genome	NC_020008.2	mt_genome	NC_020008.2
Papio papio	mt_genome	JX946203.2	mt_genome	JX946203.2	mt_genome	JX946203.2	mt_genome	JX946203.2	mt_genome	JX946203.2
Papio ursinus	mt_genome	NC_020010.2	mt_genome	NC_020010.2	mt_genome	NC_020010.2	mt_genome	NC_020010.2	mt_genome	NC_020010.2
Theropithecus gelada	mt_genome	NC_019802.1	mt_genome	NC_019802.1	mt_genome	NC_019802.1	mt_genome	NC_019802.1	mt_genome	NC_019802.1
Cercocebus agilis	-	-	NCBI	EU179513.1	-	-	NCBI	FJ750706.1	NCBI	AY665622.1
Cercocebus atys	mt_genome	NC_028592.1	mt_genome	NC_028592.1	mt_genome	NC_028592.1	mt_genome	NC_028592.1	mt_genome	NC_028592.1
Cercocebus chrysogaster	mt_genome	NC_021943.1	mt_genome	NC_021943.1	mt_genome	NC_021943.1	mt_genome	NC_021943.1	mt_genome	NC_021943.1

Cercocebus torquatus	mt_genome	NC_023964.1	mt_genome	NC_023964.1	mt_genome	NC_023964.1	mt_genome	NC_023964.1	mt_genome	NC_023964.1
Mandrillus sphinx	NCBI	AF301616.1	-	-	-	-	NCBI	L35196.1	-	-
Mandrillus leucophaeus	mt_genome	NC_028442.1	mt_genome	NC_028442.1	mt_genome	NC_028442.1	mt_genome	NC_028442.1	mt_genome	NC_028442.1
Colobus angolensis	NCBI	ON009457.1	-	-	-	-	-	-	-	-
Colobus guereza	NCBI	MK415379.1	-	-	-	-	NCBI	L35195.1	-	-
Colobus polykomos	NCBI	AF020411.1	NCBI	AB016731.1	-	-	-	-	-	-
Colobus caudatus	-	-	-	-	NCBI	DQ399693.1	-	-	-	-
Colobus satanas	NCBI	KJ193336.1	NCBI	KJ192796.1	-	-	NCBI	KJ192499.1	-	-
Colobus vellerosus	NCBI	PV963065.1	-	-	-	-	-	-	-	-
Piliocolobus badius	NCBI	NC_008219.1	NCBI	NC_008219.1	NCBI	NC_008219.1	NCBI	NC_008219.1	NCBI	NC_008219.1
Piliocolobus gordonorum	-	-	NCBI	MW049055.1	-	-	-	-	-	-
Piliocolobus parmentieri	-	-	-	-	NCBI	EU580068.1	-	-	-	-
Piliocolobus oustaleti	-	-	-	-	NCBI	EU580067.1	-	-	-	-
Piliocolobus pennantii	-	-	-	-	NCBI	EU580071.1	-	-	-	-
Piliocolobus preussi	-	-	-	-	NCBI	EU580074.1	-	-	-	-
Piliocolobus rufomitratus	-	-	-	-	NCBI	FJ882004.1	-	-	-	-
Piliocolobus tephrosceles	-	-	-	-	NCBI	FJ881924.1	-	-	-	-
Piliocolobus tholloni	-	-	-	-	NCBI	EU580081.1	-	-	-	-
Procolobus verus	mt_genome	NC_020666.1	mt_genome	NC_020666.1	mt_genome	NC_020666.1	mt_genome	NC_020666.1	mt_genome	NC_020666.1
Semnopithecus ajax	NCBI	PP554603.1	NCBI	PP406394.1	-	-	-	-	-	-
Semnopithecus entellus	mt_genome	NC_008215.1	mt_genome	NC_008215.1	mt_genome	NC_008215.1	mt_genome	NC_008215.1	mt_genome	NC_008215.1
Semnopithecus hector	NCBI	AY519451.1	-	-	-	-	-	-	-	-

Semnopithecus johnii	mt_genome	HQ149050.1	mt_genome	HQ149050.1	mt_genome	HQ149050.1	mt_genome	HQ149050.1	mt_genome	HQ149050.1
Semnopithecus vetulus	mt_genome	NC_019582.1	mt_genome	NC_019582.1	mt_genome	NC_019582.1	mt_genome	NC_019582.1	mt_genome	NC_019582.1
Semnopithecus hypoleucos	NCBI	MT758700.1	-	-	-	-	-	-	-	-
Semnopithecus priam	NCBI	JQ734757.1	NCBI	PQ898018.1	-	-	-	-	-	-
Semnopithecus schistaceus	NCBI	LC801087.1	-	-	-	-	-	-	-	-
Trachypithecus auratus	NCBI	AY519455.1	-	-	-	-	NCBI	L35200.1	-	-
Trachypithecus barbei	mt_genome	MF872235.1	mt_genome	MF872235.1	mt_genome	MF872235.1	mt_genome	MF872235.1	mt_genome	MF872235.1
Trachypithecus cristatus	mt_genome	NC_023971.1	mt_genome	NC_023971.1	mt_genome	NC_023971.1	mt_genome	NC_023971.1	mt_genome	NC_023971.1
Trachypithecus crepusculus	mt_genome	MF872229.1	mt_genome	MF872229.1	mt_genome	MF872229.1	mt_genome	MF872229.1	mt_genome	MF872229.1
Trachypithecus delacouri	mt_genome	MF872232.1	mt_genome	MF872232.1	mt_genome	MF872232.1	mt_genome	MF872232.1	mt_genome	MF872232.1
Trachypithecus francoisi	mt_genome	NC_023970.1	mt_genome	NC_023970.1	mt_genome	NC_023970.1	mt_genome	NC_023970.1	mt_genome	NC_023970.1
Trachypithecus geei	mt_genome	NC_056324.1	mt_genome	NC_056324.1	mt_genome	NC_056324.1	mt_genome	NC_056324.1	mt_genome	NC_056324.1
Trachypithecus germaini	mt_genome	NC_019580.1	mt_genome	NC_019580.1	mt_genome	NC_019580.1	mt_genome	NC_019580.1	mt_genome	NC_019580.1
Trachypithecus hatinhensis	mt_genome	NC_019579.1	mt_genome	NC_019579.1	mt_genome	NC_019579.1	mt_genome	NC_019579.1	mt_genome	NC_019579.1
Trachypithecus mauritius	mt_genome	NC_056325.1	mt_genome	NC_056325.1	mt_genome	NC_056325.1	mt_genome	NC_056325.1	mt_genome	NC_056325.1
Trachypithecus popa	NCBI	KC833500.1	-	-	-	-	-	-	-	-
Trachypithecus leucocephalus	NCBI	HQ613912.1	-	-	-	-	-	-	-	-
Trachypithecus laotum	mt_genome	NC_056330.1	mt_genome	NC_056330.1	mt_genome	NC_056330.1	mt_genome	NC_056330.1	mt_genome	NC_056330.1
Trachypithecus obscurus	mt_genome	MF872234.1	mt_genome	MF872234.1	mt_genome	MF872234.1	mt_genome	MF872234.1	mt_genome	MF872234.1
Trachypithecus phayrei	mt_genome	NC_056326.1	mt_genome	NC_056326.1	mt_genome	NC_056326.1	mt_genome	NC_056326.1	mt_genome	NC_056326.1
Trachypithecus pileatus	mt_genome	NC_024529.1	mt_genome	NC_024529.1	mt_genome	NC_024529.1	mt_genome	NC_024529.1	mt_genome	NC_024529.1
Trachypithecus poliocephalus	mt_genome	NC_034795.1	mt_genome	NC_034795.1	mt_genome	NC_034795.1	mt_genome	NC_034795.1	mt_genome	NC_034795.1

Trachypithecus shortridgei	mt_genome	NC_019581.1	mt_genome	NC_019581.1	mt_genome	NC_019581.1	mt_genome	NC_019581.1	mt_genome	NC_019581.1
Presbytis chrysomelas	NCBI	JF295112.1	-	-	-	-	-	-	-	-
Presbytis bicolor	NCBI	JF295109.1	-	-	-	-	-	-	-	-
Presbytis comata	mt_genome	KY117600.1	mt_genome	KY117600.1	mt_genome	KY117600.1	mt_genome	KY117600.1	mt_genome	KY117600.1
Presbytis femoralis	mt_genome	KU899140.1	mt_genome	KU899140.1	mt_genome	KU899140.1	mt_genome	KU899140.1	mt_genome	KU899140.1
Presbytis frontata	NCBI	JF295113.1	-	-	-	-	-	-	-	-
Presbytis fredericae	NCBI	OK465361.1	-	-	-	-	-	-	-	-
Presbytis hosei	NCBI	JF295114.1	-	-	-	-	-	-	-	-
Presbytis mitrata	mt_genome	KY117601.1	mt_genome	KY117601.1	mt_genome	KY117601.1	mt_genome	KY117601.1	mt_genome	KY117601.1
Presbytis melalophos	mt_genome	NC_008217.1	mt_genome	NC_008217.1	mt_genome	NC_008217.1	mt_genome	NC_008217.1	mt_genome	NC_008217.1
Presbytis potenziani	NCBI	JF295123.1	-	-	-	-	-	-	-	-
Presbytis siberu	NCBI	JF295121.1	-	-	-	-	-	-	-	-
Presbytis sumatrana	NCBI	JF295110.1	-	-	-	-	-	-	-	-
Presbytis rubicunda	NCBI	JF295111.1	NCBI	MT876703.1	-	-	-	-	-	-
Presbytis thomasi	NCBI	JF295125.1	-	-	-	-	-	-	-	-
Pygathrix nemaeus	mt_genome	NC_008220.1	mt_genome	NC_008220.1	mt_genome	NC_008220.1	mt_genome	NC_008220.1	mt_genome	NC_008220.1
Pygathrix cinerea	mt_genome	NC_088556.1	mt_genome	NC_088556.1	mt_genome	NC_088556.1	mt_genome	NC_088556.1	mt_genome	NC_088556.1
Pygathrix nigripes	mt_genome	NC_018061.1	mt_genome	NC_018061.1	mt_genome	NC_018061.1	mt_genome	NC_018061.1	mt_genome	NC_018061.1
Rhinopithecus avunculus	mt_genome	NC_015485.1	mt_genome	NC_015485.1	mt_genome	NC_015485.1	mt_genome	NC_015485.1	mt_genome	NC_015485.1
Rhinopithecus bieti	mt_genome	NC_015486.1	mt_genome	NC_015486.1	mt_genome	NC_015486.1	mt_genome	NC_015486.1	mt_genome	NC_015486.1
Rhinopithecus brelichi	mt_genome	JN540032.1	mt_genome	JN540032.1	mt_genome	JN540032.1	mt_genome	JN540032.1	mt_genome	JN540032.1
Rhinopithecus roxellana	mt_genome	KM504390.1	mt_genome	KM504390.1	mt_genome	KM504390.1	mt_genome	KM504390.1	mt_genome	KM504390.1

Rhinopithecus strykeri	mt_genome	NC_018059.1	mt_genome	NC_018059.1	mt_genome	NC_018059.1	mt_genome	NC_018059.1	mt_genome	NC_018059.1
Nasalis larvatus	mt_genome	KM889667.1	mt_genome	KM889667.1	mt_genome	KM889667.1	mt_genome	KM889667.1	mt_genome	KM889667.1
Simias concolor	mt_genome	NC_020667.1	mt_genome	NC_020667.1	mt_genome	NC_020667.1	mt_genome	NC_020667.1	mt_genome	NC_020667.1
Hylobates agilis	mt_genome	NC_014042.1	mt_genome	NC_014042.1	mt_genome	NC_014042.1	mt_genome	NC_014042.1	mt_genome	NC_014042.1
Hylobates funereus	NCBI	GU321312.1	-	-	-	-	-	-	-	-
Hylobates albibarbis	mt_genome	LC548013.1	mt_genome	LC548013.1	mt_genome	LC548013.1	mt_genome	LC548013.1	mt_genome	LC548013.1
Hylobates abbotti	mt_genome	PQ867516.1	mt_genome	PQ867516.1	mt_genome	PQ867516.1	mt_genome	PQ867516.1	mt_genome	PQ867516.1
Hylobates klossii	NCBI	GU321318.1	-	-	-	-	NCBI	AB050182.1	NCBI	AB050182.1
Hylobates lar	mt_genome	NC_002082.1	mt_genome	NC_002082.1	mt_genome	NC_002082.1	mt_genome	NC_002082.1	mt_genome	NC_002082.1
Hylobates moloch	mt_genome	LC548014.1	mt_genome	LC548014.1	mt_genome	LC548014.1	mt_genome	LC548014.1	mt_genome	LC548014.1
Hylobates muelleri	mt_genome	LC548016.1	mt_genome	LC548016.1	mt_genome	LC548016.1	mt_genome	LC548016.1	mt_genome	LC548016.1
Hylobates pileatus	mt_genome	NC_014045.1	mt_genome	NC_014045.1	mt_genome	NC_014045.1	mt_genome	NC_014045.1	mt_genome	NC_014045.1
Hoolock hoolock	mt_genome	NC_033885.1	mt_genome	NC_033885.1	mt_genome	NC_033885.1	mt_genome	NC_033885.1	mt_genome	NC_033885.1
Hoolock tianxing	mt_genome	NC_033884.1	mt_genome	NC_033884.1	mt_genome	NC_033884.1	mt_genome	NC_033884.1	mt_genome	NC_033884.1
Hoolock leuconedys	mt_genome	MT712145.1	mt_genome	MT712145.1	mt_genome	MT712145.1	mt_genome	MT712145.1	mt_genome	MT712145.1
Nomascus concolor	mt_genome	PQ740954.1	mt_genome	PQ740954.1	mt_genome	PQ740954.1	mt_genome	PQ740954.1	mt_genome	PQ740954.1
Nomascus gabriellae	mt_genome	NC_018753.1	mt_genome	NC_018753.1	mt_genome	NC_018753.1	mt_genome	NC_018753.1	mt_genome	NC_018753.1
Nomascus hainanus	NCBI	GU321248.1	-	-	-	-	-	-	-	-
Nomascus annamensis	NCBI	MN615774.1	-	-	-	-	-	-	-	-
Nomascus leucogenys	mt_genome	NC_021957.1	mt_genome	NC_021957.1	mt_genome	NC_021957.1	mt_genome	NC_021957.1	mt_genome	NC_021957.1
Nomascus nasutus	NCBI	GU594996.1	-	-	-	-	-	-	-	-
Nomascus siki	mt_genome	OM287160.1	mt_genome	OM287160.1	mt_genome	OM287160.1	mt_genome	OM287160.1	mt_genome	OM287160.1

Symphalangus syndactylus	mt_genome	NC_014047.1	mt_genome	NC_014047.1	mt_genome	NC_014047.1	mt_genome	NC_014047.1	mt_genome	NC_014047.1
Pongo abelii	mt_genome	NC_002083.1	mt_genome	NC_002083.1	mt_genome	NC_002083.1	mt_genome	NC_002083.1	mt_genome	NC_002083.1
Pongo pygmaeus	mt_genome	D38115.1	mt_genome	D38115.1	mt_genome	D38115.1	mt_genome	D38115.1	mt_genome	D38115.1
Gorilla gorilla	mt_genome	NC_001645.1	mt_genome	NC_001645.1	mt_genome	NC_001645.1	mt_genome	NC_001645.1	mt_genome	NC_001645.1
Gorilla beringei	mt_genome	NC_037853.1	mt_genome	NC_037853.1	mt_genome	NC_037853.1	mt_genome	NC_037853.1	mt_genome	NC_037853.1
Pan troglodytes	NCBI	EF660852.1	NCBI	EF554224.1	-	-	NCBI	AB062538.1	NCBI	AB062547.1
Pan paniscus	mt_genome	D38116.1	mt_genome	D38116.1	mt_genome	D38116.1	mt_genome	D38116.1	mt_genome	D38116.1

Table S2 Calibrations used for divergence time estimates of primates

Speices	Min/Max	Age (Mya)	*References*
Tarsius sangirensis loris tardigradus	Min	71.4	*125, 126, 127*
Tarsius sangirensis loris tardigradus	Max	77.5	*125, 126, 127*
Tarsius sangirensis Pithecia albicans	Min	61.6	*128, 129, 130*
Tarsius sangirensis Pithecia albicans	Max	71.1	*128, 129, 130*
Pithecia albicans Macaca thibetana	Min	40	*128, 129*
Pithecia albicans Macaca thibetana	Max	44.2	*128, 129*
Pithecia albicans Lagothrix lagotricha	Min	20.21	*128, 131, 132*
Pithecia albicans Lagothrix lagotricha	Max	21.47	*129, 131, 132*
Pithecia albicans Plecturocebus grovesi	Min	16.18	*130, 132*
Pithecia albicans Plecturocebus grovesi	Max	19.34	*130, 132*

Pithecia albicans Cacajao calvus	Min	9.34	*133, 134*
Pithecia albicans Cacajao calvus	Max	13.17	*133, 134*
Cacajao calvus Chiropotes albinasus	Min	5.6	*133, 135*
Cacajao calvus Chiropotes albinasus	Max	7.06	*133, 135*
Cheracebus regulus Plecturocebus miltoni	Min	6	*132, 136*
Cheracebus regulus Plecturocebus miltoni	Max	10.98	*132, 136*
Aotus nigriceps Lagothrix lagotricha	Min	18.1	*128, 132, 137*
Aotus nigriceps Lagothrix lagotricha	Max	21.8	*128, 132, 137*
Aotus nigriceps Sapajus xanthosternos	Min	17.1	*131, 137, 138*
Aotus nigriceps Sapajus xanthosternos	Max	19.1	*131, 137, 138*
Aotus nigriceps Saguinus tripartitus	Min	14.3	*138, 139*
Aotus nigriceps Saguinus tripartitus	Max	17.65	*138, 139*
Callithrix aurita Saguinus tripartitus	Min	13.59	*132, 134, 140*
Callithrix aurita Saguinus tripartitus	Max	15.66	*132, 134, 140*
Callithrix aurita Leontopithecus chrysopygus	Min	10.9	*132, 135*
Callithrix aurita Leontopithecus chrysopygus	Max	13.85	*132, 135*
Callithrix aurita Callimico goeldii	Min	10.88	*132, 141*

Callithrix aurita<br>*Callimico goeldii*	Max	11.83	*132, 141*
Saimiri collinsi<br>*Cebus olivaceus*	Min	14.45	*132, 140*
Saimiri collinsi<br>*Cebus olivaceus*	Max	16.32	*132, 140*
Cebus olivaceus<br>*Sapajus xanthosternos*	Min	5.39	*134, 143*
Cebus olivaceus<br>*Sapajus xanthosternos*	Max	7.5	*135, 142*
Alouatta macconnelli<br>*Lagothrix lagotricha*	Min	13.09	*132, 133, 135*
Alouatta macconnelli<br>*Lagothrix lagotricha*	Max	16.75	*132, 133, 135*
Lagothrix lagotricha<br>*Ateles marginatus*	Min	10.5	*132, 133, 134*
Lagothrix lagotricha<br>*Ateles marginatus*	Max	13.3	*132, 133, 134*
Brachyteles arachnoides<br>*Lagothrix lagotricha*	Min	8.58	*132, 133*
Brachyteles arachnoides<br>*Lagothrix lagotricha*	Max	11.57	*132, 133*
Nomascus leucogenys<br>*Hoolock hoolock*	Min	7.25	*143, 144*
Nomascus leucogenys<br>*Hoolock hoolock*	Max	14.04	*143, 144*
Symphalangus syndactylus<br>*Hylobates pileatus*	Min	6.39	*143, 144*
Symphalangus syndactylus<br>*Hylobates pileatus*	Max	8	*143, 144*
Pongo pygmaeus<br>*Hoolock hoolock*	Min	18.8	*131, 145*
Pongo pygmaeus<br>*Hoolock hoolock*	Max	20.3	*131, 145*

Pan paniscus Pongo pygmaeus	Min	14.5	*145, 146*
Pan paniscus Pongo pygmaeus	Max	16.3	*145, 146*
Pan paniscus Gorilla gorilla	Min	8.25	*127, 147*
Pan paniscus Gorilla gorilla	Max	9.3	*127, 147*
Hoolock hoolock Macaca thibetana	Min	26.8	*129, 148*
Hoolock hoolock Macaca thibetana	Max	30.6	*129, 148*
Allenopithecus nigroviridis Chlorocebus cynosuros	Min	7.21	*132, 144*
Allenopithecus nigroviridis Chlorocebus cynosuros	Max	14.1	*132, 144*
Miopithecus talapoin Chlorocebus cynosuros	Min	7.55	*132, 141, 144*
Miopithecus talapoin Chlorocebus cynosuros	Max	12.95	*132, 141, 144*
Allenopithecus nigroviridis Macaca thibetana	Min	11.5	*144, 149*
Allenopithecus nigroviridis Macaca thibetana	Max	13.09	*144, 149*
Macaca thibetana Papio papio	Min	8.79	*144, 149*
Macaca thibetana Papio papio	Max	11.05	*144, 149*
Cercocebus agilis Papio papio	Min	6.1	*144, 150*
Cercocebus agilis Papio papio	Max	11.74	*144, 150*
Rungwecebus kipunji Papio papio	Min	1.76	*132, 141, 144*

Rungwecebus kipunji *Papio papio*	Max	3.70	*132, 141, 144*
Cercocebus agilis *Mandrillus sphinx*	Min	3.9	*151*
Cercocebus agilis *Mandrillus sphinx*	Max	5.46	*151*
Trachypithecus leucocephalus *Macaca thibetana*	Min	15.5	*152*
Trachypithecus leucocephalus *Macaca thibetana*	Max	19.8	*151*
Piliocolobus tephrosceles *Trachypithecus leucocephalus*	Min	10.32	*132, 153*
Piliocolobus tephrosceles *Trachypithecus leucocephalus*	Max	14.12	*132, 153*
Colobus angolensis *Piliocolobus tephrosceles*	Min	8.3	*132, 153, 154*
Colobus angolensis *Piliocolobus tephrosceles*	Max	11.32	*132, 153, 154*
Hylobates albibarbis *Hylobates agilis*	Min	0.95	*155, 156*
Hylobates albibarbis *Hylobates agilis*	Max	1.56	*155, 156*
Macaca ochreata *Macaca brunnescens*	Min	1.67	*144, 156*
Macaca ochreata *Macaca brunnescens*	Max	4.7	*144, 156*
Macaca mulatta *Macaca cyclopis*	Min	1.67	*132, 144*
Macaca mulatta *Macaca cyclopis*	Max	4.7	*132, 144*
Rhinopithecus brelichi *Rhinopithecus roxellana*	Min	1.74	*157, 158*

Rhinopithecus brelichi<br>*Rhinopithecus roxellana*	Max	2.19	*157, 158*
Galagoides thomasi<br>*Galagoides demidoff*	Min	4.93	*159, 160*
Galagoides thomasi<br>*Galagoides demidoff*	Max	12.1	*159, 160*

Table S3 Environmental variables used in this study

Climatic variables	Definition
BIO1	Annual mean temperature
BIO2	Mean diurnal range
BIO3	Isothermality (BIO2/BIO7)
BIO4	Temperature Seasonality
BIO5	Maximum Temperature of Warmest Month
BIO6	Minimum Temperature of Coldest Month
BIO7	Temperature Annual Range
BIO8	Mean Temperature of Wettest Quarter
BIO9	Mean Temperature of Driest Quarter
BIO10	Mean Temperature of Warmest Quarter
BIO11	Mean Temperature of Coldest Quarter
BIO12	Annual Precipitation
BIO13	Precipitation of Wettest Month
BIO14	Precipitation of Driest Month
BIO15	Precipitation Seasonality
BIO16	Precipitation of Wettest Quarter
BIO17	Precipitation of Driest Quarter
BIO18	Precipitation of Warmest Quarter
BIO19	Precipitation of Coldest Quarter
LULC	Land use and land cover
Velocity	Climate Velocity
NPP	Net Primary Productivity
TRI	Terrain Ruggedness Index

Table S4 BAMM sampling fractions at the genus level

Species Name	Clade Name	Sampling Fraction

Perodicticus potto	Perodicticus	0.33
Arctocebus calabarensis	Arctocebus	1.00
Arctocebus aureus	Arctocebus	1.00
Nycticebus pygmaeus	Nycticebus	0.56
Nycticebus bengalensis	Nycticebus	0.56
Nycticebus coucang	Nycticebus	0.56
Nycticebus javanicus	Nycticebus	0.56
Nycticebus menagensis	Nycticebus	0.56
Loris tardigradus	Loris	1.00
Loris lydekkerianus	Loris	1.00
Euoticus elegantulus	Euoticus	0.50
Galagoides demidoff	Galagoides	0.50
Galagoides thomasi	Galagoides	0.50
Paragalago zanzibaricus	Paragalago	1.00
Paragalago cocos	Paragalago	1.00
Paragalago granti	Paragalago	1.00
Paragalago orinus	Paragalago	1.00
Paragalago rondoensis	Paragalago	1.00
Galago senegalensis	Galago	1.00
Galago gallarum	Galago	1.00
Galago moholi	Galago	1.00
Galago matschiei	Galago	1.00
Sciurocheirus alleni	Sciurocheirus	0.67
Sciurocheirus gabonensis	Sciurocheirus	0.67
Otolemur garnettii	Otolemur	1.00
Otolemur crassicaudatus	Otolemur	1.00
Daubentonia madagascariensis	Daubentonia	1.00
Avahi ramanantsoavani	Avahi	0.78
Avahi meridionalis	Avahi	0.78
Avahi peyrierasi	Avahi	0.78
Avahi laniger	Avahi	0.78
Avahi cleesei	Avahi	0.78
Avahi unicolor	Avahi	0.78
Avahi occidentalis	Avahi	0.78
Propithecus deckenii	Propithecus	0.89
Propithecus verreauxi	Propithecus	0.89
Propithecus candidus	Propithecus	0.89
Propithecus coquereli	Propithecus	0.89
Propithecus tattersalli	Propithecus	0.89
Propithecus edwardsi	Propithecus	0.89
Propithecus diadema	Propithecus	0.89
Propithecus perrieri	Propithecus	0.89
Indri indri	Indri	1.00

Palaeopropithecus ingens	Palaeopropithecus	1.00
Phaner furcifer	Phaner	0.25
Mirza coquereli	Mirza	1.00
Mirza zaza	Mirza	1.00
Microcebus berthae	Microcebus	0.92
Microcebus myoxinus	Microcebus	0.92
Microcebus lehilahytsara	Microcebus	0.92
Microcebus jonahi	Microcebus	0.92
Microcebus ganzhorni	Microcebus	0.92
Microcebus arnholdi	Microcebus	0.92
Microcebus sambiranensis	Microcebus	0.92
Microcebus margotmarshae	Microcebus	0.92
Microcebus mamiratra	Microcebus	0.92
Microcebus rufus	Microcebus	0.92
Microcebus tanosi	Microcebus	0.92
Microcebus marohita	Microcebus	0.92
Microcebus gerpi	Microcebus	0.92
Microcebus jollyae	Microcebus	0.92
Microcebus simmonsi	Microcebus	0.92
Microcebus tavaratra	Microcebus	0.92
Microcebus bongolavensis	Microcebus	0.92
Microcebus ravelobensis	Microcebus	0.92
Microcebus danfossi	Microcebus	0.92
Microcebus macarthurii	Microcebus	0.92
Microcebus griseorufus	Microcebus	0.92
Microcebus murinus	Microcebus	0.92
Allocebus trichotis	Allocebus	1.00
Cheirogaleus thomasi	Cheirogaleus	0.67
Cheirogaleus crossleyi	Cheirogaleus	0.67
Cheirogaleus lavasoensis	Cheirogaleus	0.67
Cheirogaleus major	Cheirogaleus	0.67
Cheirogaleus medius	Cheirogaleus	0.67
Cheirogaleus sibreei	Cheirogaleus	0.67
Lepilemur otto	Lepilemur	1.00
Lepilemur grewcockorum	Lepilemur	1.00
Lepilemur scottorum	Lepilemur	1.00
Lepilemur edwardsi	Lepilemur	1.00
Lepilemur microdon	Lepilemur	1.00
Lepilemur hollandorum	Lepilemur	1.00
Lepilemur milanoii	Lepilemur	1.00
Lepilemur ankaranensis	Lepilemur	1.00
Lepilemur ahmansonorum	Lepilemur	1.00
Lepilemur sahamalazensis	Lepilemur	1.00
Lepilemur tymerlachsoni	Lepilemur	1.00

Lepilemur dorsalis	Lepilemur	1.00
Lepilemur septentrionalis	Lepilemur	1.00
Lepilemur hubbardorum	Lepilemur	1.00
Lepilemur ruficaudatus	Lepilemur	1.00
Lepilemur randrianasoloi	Lepilemur	1.00
Lepilemur aeeclis	Lepilemur	1.00
Lepilemur petteri	Lepilemur	1.00
Lepilemur leucopus	Lepilemur	1.00
Lepilemur betsileo	Lepilemur	1.00
Lepilemur jamesorum	Lepilemur	1.00
Lepilemur mustelinus	Lepilemur	1.00
Lepilemur fleuretae	Lepilemur	1.00
Lepilemur wrightae	Lepilemur	1.00
Lepilemur seali	Lepilemur	1.00
Varecia variegata	Varecia	1.00
Varecia rubra	Varecia	1.00
Prolemur simus	Prolemur	q.00
Hapalemur occidentalis	Hapalemur	1.00
Hapalemur alaotrensis	Hapalemur	1.00
Hapalemur griseus	Hapalemur	1.00
Hapalemur meridionalis	Hapalemur	1.00
Hapalemur aureus	Hapalemur	1.00
Lemur catta	Lemur	1.00
Eulemur mongoz	Eulemur	1.00
Eulemur rubriventer	Eulemur	1.00
Eulemur rufus	Eulemur	1.00
Eulemur rufifrons	Eulemur	1.00
Eulemur albifrons	Eulemur	1.00
Eulemur sanfordi	Eulemur	1.00
Eulemur cinereiceps	Eulemur	1.00
Eulemur fulvus	Eulemur	1.00
Eulemur collaris	Eulemur	1.00
Eulemur flavifrons	Eulemur	1.00
Eulemur macaco	Eulemur	1.00
Eulemur coronatus	Eulemur	1.00
Pithecia vanzolinii	Pithecia	0.63
Pithecia hirsuta	Pithecia	0.63
Pithecia mittermeieri	Pithecia	0.63
Pithecia milleri	Pithecia	0.63
Pithecia pissinatti	Pithecia	0.63
Pithecia albicans	Pithecia	0.63
Pithecia chrysocephala	Pithecia	0.63
Pithecia monachus	Pithecia	0.63
Pithecia irrorata	Pithecia	0.63

Pithecia pithecia	Pithecia	0.63
Cacajao rubicundus	Cacajao	0.72
Cacajao calvus	Cacajao	0.72
Cacajao hosomi	Cacajao	0.72
Cacajao ayresi	Cacajao	0.72
Cacajao melanocephalus	Cacajao	0.72
Chiropotes albinasus	Chiropotes	1.00
Chiropotes chiropotes	Chiropotes	1.00
Chiropotes israelita	Chiropotes	1.00
Chiropotes utahickae	Chiropotes	1.00
Chiropotes satanas	Chiropotes	1.00
Cheracebus regulus	Cheracebus	1.00
Cheracebus torquatus	Cheracebus	1.00
Cheracebus lucifer	Cheracebus	1.00
Cheracebus medemi	Cheracebus	1.00
Cheracebus lugens	Cheracebus	1.00
Plecturocebus miltoni	Plecturocebus	0.44
Plecturocebus cinerascens	Plecturocebus	0.44
Plecturocebus grovesi	Plecturocebus	0.44
Plecturocebus moloch	Plecturocebus	0.44
Plecturocebus bernhardi	Plecturocebus	0.44
Plecturocebus hoffmannsi	Plecturocebus	0.44
Plecturocebus brunneus	Plecturocebus	0.44
Plecturocebus cupreus	Plecturocebus	0.44
Plecturocebus dubius	Plecturocebus	0.44
Plecturocebus caligatus	Plecturocebus	0.44
Plecturocebus donacophilus	Plecturocebus	0.44
Callicebus coimbrai	Callicebus	0.60
Callicebus personatus	Callicebus	0.60
Callicebus nigrifrons	Callicebus	0.60
Aotus nigriceps	Aotus	0.73
Aotus azarai	Aotus	0.73
Aotus lemurinus	Aotus	0.73
Aotus brumbacki	Aotus	0.73
Aotus griseimembra	Aotus	0.73
Aotus trivirgatus	Aotus	0.73
Aotus vociferans	Aotus	0.73
Aotus nancymaae	Aotus	0.73
Mico humeralifer	Mico	0.60
Mico mauesi	Mico	0.60
Mico emiliae	Mico	0.60
Mico argentatus	Mico	0.60
Mico chrysoleucos	Mico	0.60
Calibella humilis	Calibella	1.00

Mico melanurus	Mico	0.60
Cebuella pygmaea	Cebuella	1.00
Cebuella niveiventris	Cebuella	1.00
Mico marcai	Mico	0.60
Callithrix aurita	Callithrix	0.83
Callithrix penicillata	Callithrix	0.83
Callithrix jacchus	Callithrix	0.83
Callithrix kuhlii	Callithrix	0.83
Callithrix geoffroyi	Callithrix	0.83
Mico saterei	Mico	0.60
Mico rondoni	Mico	0.60
Callimico goeldii	Callimico	1.00
Leontopithecus chrysomelas	Leontopithecus	0.75
Leontopithecus chrysopygus	Leontopithecus	0.75
Leontopithecus rosalia	Leontopithecus	0.75
Leontocebus fuscicollis	Leontocebus	1.00
Leontocebus leucogenys	Leontocebus	1.00
Leontocebus nigrifrons	Leontocebus	1.00
Saguinus tripartitus	Saguinus	0.016949
Leontocebus weddelli	Leontocebus	1.00
Leontocebus fuscus	Leontocebus	1.00
Leontocebus cruzlimai	Leontocebus	1.00
Leontocebus nigricollis	Leontocebus	1.00
Leontocebus lagonotus	Leontocebus	1.00
Leontocebus tripartitus	Leontocebus	1.00
Leontocebus illigeri	Leontocebus	1.00
Saguinus mystax	Saguinus	1.00
Tamarinus labiatus	Tamarinus	0.75
Tamarinus imperator	Tamarinus	0.75
Oedipomidus leucopus	Oedipomidus	1.00
Saguinus midas	Saguinus	1.00
Saguinus niger	Saguinus	1.00
Saguinus bicolor	Saguinus	1.00
Saguinus ursulus	Saguinus	1.00
Saguinus martinsi	Saguinus	1.00
Oedipomidus oedipus	Oedipomidus	1.00
Oedipomidus geoffroyi	Oedipomidus	1.00
Tamarinus inustus	Tamarinus	0.75
Cebus olivaceus	Cebus	0.31
Cebus kaapori	Cebus	0.31
Cebus unicolor	Cebus	0.31
Cebus capucinus	Cebus	0.31
Cebus albifrons	Cebus	0.31
Sapajus xanthosternos	Sapajus	0.86

Sapajus flavius	Sapajus	0.86
Sapajus apella	Sapajus	0.86
Sapajus cay	Sapajus	0.86
Sapajus libidinosus	Sapajus	0.86
Sapajus nigritus	Sapajus	0.86
Saimiri collinsi	Saimiri	1.00
Saimiri ustus	Saimiri	1.00
Saimiri oerstedii	Saimiri	1.00
Saimiri sciureus	Saimiri	1.00
Saimiri macrodon	Saimiri	1.00
Saimiri cassiquiarensis	Saimiri	1.00
Saimiri vanzolinii	Saimiri	1.00
Saimiri boliviensis	Saimiri	1.00
Ateles marginatus	Ateles	1.00
Ateles chamek	Ateles	1.00
Ateles paniscus	Ateles	1.00
Ateles geoffroyi	Ateles	1.00
Ateles belzebuth	Ateles	1.00
Ateles hybridus	Ateles	1.00
Ateles fusciceps	Ateles	1.00
Brachyteles arachnoides	Brachyteles	1.00
Brachyteles hypoxanthus	Brachyteles	1.00
Lagothrix lagotricha	Lagothrix	1.00
Alouatta macconnelli	Alouatta	0.71
Alouatta seniculus	Alouatta	0.71
Alouatta sara	Alouatta	0.71
Alouatta juara	Alouatta	0.71
Alouatta caraya	Alouatta	0.71
Alouatta discolor	Alouatta	0.71
Alouatta belzebul	Alouatta	0.71
Alouatta guariba	Alouatta	0.71
Alouatta pigra	Alouatta	0.71
Alouatta palliata	Alouatta	0.71
Hoolock hoolock	Hoolock	0.67
Hoolock leuconedys	Hoolock	0.67
Symphalangus syndactylus	Symphalangus	1.00
Hylobates pileatus	Hylobates	0.78
Hylobates albibarbis	Hylobates	0.78
Hylobates agilis	Hylobates	0.78
Hylobates muelleri	Hylobates	0.78
Hylobates moloch	Hylobates	0.78
Hylobates klossii	Hylobates	0.78
Hylobates lar	Hylobates	0.78
Nomascus leucogenys	Nomascus	0.86

Nomascus siki	Nomascus	0.86
Nomascus gabriellae	Nomascus	0.86
Nomascus concolor	Nomascus	0.86
Nomascus hainanus	Nomascus	0.86
Nomascus nasutus	Nomascus	0.86
Gorilla gorilla	Gorilla	1.00
Gorilla beringei	Gorilla	1.00
Pan paniscus	Pan	1.00
Pan troglodytes	Pan	1.00
Pongo pygmaeus	Pongo	0.67
Pongo abelii	Pongo	0.67
Chlorocebus cynosuros	Chlorocebus	0.86
Chlorocebus tantalus	Chlorocebus	0.86
Chlorocebus aethiops	Chlorocebus	0.86
Chlorocebus pygerythrus	Chlorocebus	0.86
Chlorocebus sabaeus	Chlorocebus	0.86
Chlorocebus dryas	Chlorocebus	0.86
Cercopithecus campbelli	Cercopithecus	0.95
Erythrocebus patas	Erythrocebus	0.33
Allochrocebus solatus	Allochrocebus	1.00
Allochrocebus preussi	Allochrocebus	1.00
Allochrocebus lhoesti	Allochrocebus	1.00
Cercopithecus erythrogaster	Cercopithecus	0.95
Cercopithecus erythrotis	Cercopithecus	0.95
Cercopithecus cephus	Cercopithecus	0.95
Cercopithecus ascanius	Cercopithecus	0.95
Cercopithecus petaurista	Cercopithecus	0.95
Cercopithecus mitis	Cercopithecus	0.95
Cercopithecus nictitans	Cercopithecus	0.95
Cercopithecus lowe	Cercopithecus	0.95
Cercopithecus diana	Cercopithecus	0.95
Cercopithecus roloway	Cercopithecus	0.95
Cercopithecus wolfi	Cercopithecus	0.95
Cercopithecus denti	Cercopithecus	0.95
Cercopithecus mona	Cercopithecus	0.95
Cercopithecus neglectus	Cercopithecus	0.95
Cercopithecus hamlyni	Cercopithecus	0.95
Cercopithecus lomamiensis	Cercopithecus	0.95
Cercopithecus pogonias	Cercopithecus	0.95
Miopithecus talapoin	Miopithecus	1.00
Miopithecus ogouensis	Miopithecus	1.00
Allenopithecus nigroviridis	Allenopithecus	1.00
Rungwecebus kipunji	Rungwecebus	1.00
Papio kindae	Papio	1.00

Papio papio	Papio	1.00
Papio ursinus	Papio	1.00
Papio cynocephalus	Papio	1.00
Papio hamadryas	Papio	1.00
Papio anubis	Papio	1.00
Theropithecus gelada	Theropithecus	1.00
Lophocebus aterrimus	Lophocebus	1.00
Lophocebus albigena	Lophocebus	1.00
Mandrillus sphinx	Mandrillus	1.00
Mandrillus leucophaeus	Mandrillus	1.00
Cercocebus agilis	Cercocebus	0.71
Cercocebus galeritus	Cercocebus	0.71
Cercocebus chrysogaster	Cercocebus	0.71
Cercocebus atys	Cercocebus	0.71
Cercocebus torquatus	Cercocebus	0.71
Macaca sylvanus	Macaca	1.00
Macaca thibetana	Macaca	1.00
Macaca assamensis	Macaca	1.00
Macaca sinica	Macaca	1.00
Macaca munzala	Macaca	1.00
Macaca radiata	Macaca	1.00
Macaca leucogenys	Macaca	1.00
Macaca arctoides	Macaca	1.00
Macaca mulatta	Macaca	1.00
Macaca cyclopis	Macaca	1.00
Macaca fuscata	Macaca	1.00
Macaca fascicularis	Macaca	1.00
Macaca silenus	Macaca	1.00
Macaca nemestrina	Macaca	1.00
Macaca siberu	Macaca	1.00
Macaca leonina	Macaca	1.00
Macaca pagensis	Macaca	1.00
Macaca nigra	Macaca	1.00
Macaca tonkeana	Macaca	1.00
Macaca nigrescens	Macaca	1.00
Macaca hecki	Macaca	1.00
Macaca ochreata	Macaca	1.00
Macaca brunnescens	Macaca	1.00
Macaca maura	Macaca	1.00
Semnopithecus hector	Semnopithecus	0.63
Semnopithecus entellus	Semnopithecus	0.63
Semnopithecus johnii	Semnopithecus	0.63
Semnopithecus priam	Semnopithecus	0.63
Semnopithecus vetulus	Semnopithecus	0.63

Trachypithecus leucocephalus	Trachypithecus	0.95
Trachypithecus phayrei	Trachypithecus	0.95
Trachypithecus barbei	Trachypithecus	0.95
Trachypithecus obscurus	Trachypithecus	0.95
Trachypithecus auratus	Trachypithecus	0.95
Trachypithecus mauritius	Trachypithecus	0.95
Trachypithecus selangorensis	Trachypithecus	0.95
Trachypithecus cristatus	Trachypithecus	0.95
Trachypithecus germaini	Trachypithecus	0.95
Trachypithecus francoisi	Trachypithecus	0.95
Trachypithecus poliocephalus	Trachypithecus	0.95
Trachypithecus delacouri	Trachypithecus	0.95
Trachypithecus ebenus	Trachypithecus	0.95
Trachypithecus hatinhensis	Trachypithecus	0.95
Trachypithecus laotum	Trachypithecus	0.95
Trachypithecus crepuscula	Trachypithecus	0.95
Trachypithecus geei	Trachypithecus	0.95
Trachypithecus pileatus	Trachypithecus	0.95
Trachypithecus shortridgei	Trachypithecus	0.95
Presbytis thomasi	Presbytis	0.55
Presbytis frontata	Presbytis	0.55
Presbytis chrysomelas	Presbytis	0.55
Presbytis hosei	Presbytis	0.55
Presbytis rubicunda	Presbytis	0.55
Presbytis bicolor	Presbytis	0.55
Presbytis melalophos	Presbytis	0.55
Presbytis comata	Presbytis	0.55
Presbytis melalophos	Presbytis	0.55
Presbytis potenziani	Presbytis	0.55
Presbytis femoralis	Presbytis	0.55
Simias concolor	Simias	1.00
Nasalis larvatus	Nasalis	1.00
Pygathrix nigripes	Pygathrix	1.00
Pygathrix cinerea	Pygathrix	1.00
Pygathrix nemaeus	Pygathrix	1.00
Rhinopithecus avunculus	Rhinopithecus	1.00
Rhinopithecus brelichi	Rhinopithecus	1.00
Rhinopithecus roxellana	Rhinopithecus	1.00
Rhinopithecus strykeri	Rhinopithecus	1.00
Rhinopithecus bieti	Rhinopithecus	1.00
Procolobus verus	Procolobus	1.00
Piliocolobus badius	Piliocolobus	0.53
Piliocolobus preussi	Piliocolobus	0.53
Piliocolobus pennantii	Piliocolobus	0.53

Piliocolobus tholloni	Piliocolobus	0.53
Piliocolobus tephrosceles	Piliocolobus	0.53
Piliocolobus rufomitratus	Piliocolobus	0.53
Piliocolobus foai	Piliocolobus	0.53
Piliocolobus kirkii	Piliocolobus	0.53
Piliocolobus gordonorum	Piliocolobus	0.53
Colobus satanas	Colobus	0.83
Colobus vellerosus	Colobus	0.83
Colobus polykomos	Colobus	0.83
Colobus guereza	Colobus	0.83
Colobus angolensis	Colobus	0.83
Tarsius fuscus	Tarsius	0.50
Tarsius dentatus	Tarsius	0.50
Tarsius sangirensis	Tarsius	0.50
Tarsius lariang	Tarsius	0.50
Tarsius wallacei	Tarsius	0.50
Tarsius tarsier	Tarsius	0.50
Carlito syrichta	Carlito	1.00
Cephalopachus bancanus	Cephalopachus	1.00

Table S5 Generalized linear mixed model measuring the relationships between species richness and key environmental variables in the final model

Model	Fixed effects	Estimate	Std.error	Marginal R2	Conditional R2
The top model	Bio3***	7.099e-01	3.645e-02	0.114	0.364
	Bio7***	-3.962e-01	2.355e-02		
	Bio12***	6.945e-01	2.781e-02		
	Bio15***	-4.037e-01	2.322e-02		
	NPP***	4.636e-01	3.623e-02		
	TRI***	-1.126e-01	2.303e-02		
	Velocity*	-5.847e-02	2.482e-02		

The significant fix terms in the models: *** $p < 0.001$; ** $p < 0.01$; * $p < 0.05$

REFERENCES

1. S. L. Pimm, C. N. Jenkins, R. Abell, T. M. Brooks, J. L. Gittleman, L. N. Joppa, P. H. Raven, C. M. Roberts, J. O. Sexton, The biodiversity of species and their rates of extinction, distribution, and protection. *Science* **344**, 1246752 (2014).

2. M. C. Urban, Accelerating extinction risk from climate change. *Science* **348**, 571–573 (2015).

3. D. Pincheira-Donoso, L. P. Harvey, J. Guirguis, L. E. B. Goodyear, C. Finn, J. V. Johnson, F. Grattarola, Temporal and spatial patterns of vertebrate extinctions during the Anthropocene. bioRxiv 2022.05.05.490605 [Preprint] (2022). https://doi.org/10.1101/2022.05.05.490605.

4. M. Pacifici, W. B. Foden, P. Visconti, J. E. M. Watson, S. H. M. Butchart, K. M. Kovacs, B. R. Scheffers, D. G. Hole, T. G. Martin, H. R. Akcakaya, R. T. Corlett, B. Huntley, D. Bickford, J. A. Carr, A. A. Hoffmann, G. F. Midgley, P. Pearce-Kelly, R. G. Pearson, S. E. Williams, S. G. Willis, B. Young, C. Rondinini, Assessing species vulnerability to climate change. *Nat. Clim. Chang.* **5**, 215–224 (2015).

5. C. D. Thomas, A. Cameron, R. E. Green, M. Bakkenes, L. J. Beaumont, Y. C. Collingham, B. F. N. Erasmus, M. F. Siqueira, A. Grainger, L. Hannah, L. Hughes, B. Huntley, A. S. Jaarsveld, G. F. Midgley, L. Miles, M. A. Ortega-Huerta, A. T. Peterson, O. L. Phillips, S. E. Williams, Extinction risk from climate change. *Nature* **427**, 145–148 (2004).

6. H. O. Portner, R. J. Scholes, A. Arneth, D. K. A. Barnes, M. T. Burrows, S. E. Diamond, C. M. Duarte, W. Kissling, P. Leadley, S. Managi, P. Mcelwee, G. Midgley, H. T. Ngo, D. Obura, U. Pascual, M. Sankaran, Y. J. Shin, A. L. Val, Overcoming the coupled climate and biodiversity crises and their societal impacts. *Science* **380**, eabl4881 (2023).

7. M. F. Biber, A. Voskamp, C. Hof, Potential effects of future climate change on global reptile distributions and diversity. *Glob. Ecol. Biogeogr.* **32**, 519–534 (2023).

8. C. R. Mi, L. Ma, M. Y. Yang, X. H. Li, S. Meiri, U. Roll, O. Oskyrko, D. Pincheira-Donoso, L. P. Harvey, D. J. Jablonski, B. Safaei-Mahroo, H. Ghaffari, J. Smid, S. Jarvie, R. M. Kimani, R. Masroor, S. M. Kazemi, L. M. Nneji, A. M. T. Fokoua, G. C. T. Taboue, A. Bauer, C. Nogueira, D. Meirte, D. G. Chapple, I. Das, L. Grismer, L. J. Avila, M. A. R. Junior, O. J. S.

Tallowin, O. Torres-Carvajal, P. Wagner, S. R. Ron, Y. Z. Wang, Y. Itescu, Z. T. Nagy, D. S. Wilcove, X. Liu, W. G. Du, Global protected areas as refuges for amphibians and reptiles under climate change. *Nat. Commun.* **14**, 1389 (2023).

9. W. Thuiller, S. Lavergne, C. Roquet, I. Boulangeat, B. Lafourcade, M. B. Araujo, Consequences of climate change on the tree of life in Europe. *Nature* **470**, 531–534 (2011).

10. N. J. B. Isaac, S. T. Turvey, B. Collen, C. Waterman, J. E. M. Baillie, Mammals on the EDGE: Conservation priorities based on threat and phylogeny. *PLOS ONE* **2**, e296 (2007).

11. M. Winter, V. Devictor, O. Schweiger, Phylogenetic diversity and nature conservation: Where are we? *Trends Ecol. Evol.* **28**, 199–204 (2013).

12. A. Purvis, P. M. Agapow, J. L. Gittleman, G. M. Mace, Nonrandom extinction and the loss of evolutionary history. *Science* **288**, 328–330 (2000).

13. C. M. Tucker, M. W. Cadotte, S. B. Carvalho, T. J. Davies, S. Ferrier, S. A. Fritz, R. Grenyer, M. R. Helmus, L. S. Jin, A. O. Mooers, S. Pavoine, O. Purschke, D. W. Redding, D. F. Rosauer, M. Winter, F. Mazel, A guide to phylogenetic metrics for conservation, community ecology and macroecology. *Biol. Rev.* **92**, 698–715 (2017).

14. D. P. Faith, Conservation evaluation and phylogenetic diversity. *Biol. Conserv.* **61**, 1–10 (1992).

15. F. Forest, R. Grenyer, M. Rouget, T. J. Davies, R. M. Cowling, D. P. Faith, A. Balmford, J. C. Manning, S. Proches, M. van der Bank, G. Reeves, T. A. J. Hedderson, V. Savolainen, Preserving the evolutionary potential of floras in biodiversity hotspots. *Nature* **445**, 757–760 (2007).

16. D. V. Pio, O. Broennimann, T. G. Barraclough, G. Reeves, A. G. Rebelo, W. Thuiller, A. Guisan, N. Salamin, Spatial predictions of phylogenetic diversity in conservation decision making. *Conserv. Biol.* **25**, 1229–1239 (2011).

17. A. M. Mendoza, H. T. Arita, Priority setting by sites and by species using rarity, richness and phylogenetic diversity: The case of neotropical glassfrogs (Anura: Centrolenidae). *Biodivers. Conserv.* **23**, 909–926 (2014).

18. A. Voskamp, D. J. Baker, P. A. Stephens, P. J. Valdes, S. G. Willis, Global patterns in the divergence between phylogenetic diversity and species richness in terrestrial birds. *J. Biogeogr.* **44**, 709–721 (2017).

19. M. W. Cadotte, B. J. Cardinale, T. H. Oakley, Evolutionary history and the effect of biodiversity on plant productivity. *Proc. Natl. Acad. Sci. U.S.A.* **105**, 17012–17017 (2008).

20. O. Schweiger, S. Klotz, W. Durka, I. Kuhn, A comparative test of phylogenetic diversity indices. *Oecologia* **157**, 485–495 (2008).

21. K. J. Gaston, S. F. Jackson, L. Cantu-Salazar, G. Cruz-Piñón, The ecological performance of protected areas. *Annu. Rev. Ecol. Evol. Syst.* **39**, 93–113 (2008).

22. S. Saout, M. Hoffmann, Y. C. Shi, A. Hughes, C. Bernard, T. M. Brooks, B. Bertzky, S. H. M. Butchart, S. N. Stuart, T. Badman, S. L. Rodrigues, Protected areas and effective biodiversity conservation. *Science* **342**, 803–805 (2013).

23. J. E. M. Watson, N. Dudley, D. B. Segan, M. Hockings, Leveraging long-term data to improve biodiversity monitoring with species distribution models Leveraging long-term data to improve biodiversity monitoring with species distribution models of protected areas. *Nature* **515**, 67–73 (2014).

24. M. B. Araújo, D. Alagador, M. Cabeza, D. Nogués-Bravo, W. Thuiller, Climate change threatens European conservation areas. *Ecol. Lett.* **14**, 484–492 (2011).

25. J. Chen, J. Zhang, J. Jiang, S. Nielsen, F. He, Assessing the effectiveness of China's protected areas to conserve current and future amphibian diversity. *Divers Distrib.* **23**, 146–157 (2017).

26. C. N. Jenkins, L. Joppa, Expansion of the global terrestrial protected area system. *Biol. Conserv.* **142**, 2166–2174 (2009).

27. G. G. Gurney, V. M. Adams, J. G. Alvarez-Romero, J. Claudet, Area-based conservation: Taking stock and looking ahead. *One Earth* **6**, 98–104 (2023).

28. G. Baldi, M. Texeira, O. A. Martin, H. R. Grau, E. G. Jobbagy, Opportunities drive the global distribution of protected areas. *PeerJ* **5**, e2989 (2017).

29. J. Monzón, L. Moyer-Horner, M. B. Palamar, Climate change and speices range dynamics in protected areas. *BioScience* **61**, 752–761 (2011).

30. M. B. Araújo, M. Cabeza, W. Thuiller, L. Hannah, P. H. Williams, Would climate change drive species out of reserves? An assessment of existing reserve-selection methods. *Glob. Chang. Biol.* **10**, 1618–1626 (2004).

31. T. Newbold, P. Oppenheimer, A. Etard, J. J. Williams, Tropical and Mediterranean biodiversity is disproportionately sensitive to land-use and climate change. *Nat. Ecol. Evol.* **4**, 1630–1638 (2020).

32. X. Liu, T. M. Blackburn, T. J. Song, X. Y. Wang, C. Huang, Y. M. Li, Animal invaders threaten protected areas worldwide. *Nat. Commun.* **11**, 2892 (2020).

33. C. J. Carlson, G. F. Albery, C. Merow, C. H. Trisos, C. M. Zipfel, E. A. Eskew, K. J. Olival, N. Ross, S. Bansal, Climate change increases cross-species viral transmission risk. *Nature* **607**, 555–562 (2022).

34. B. Kellenberger, K. Winner, W. Jetz, The performance and potential of deep learning for predicting species distributions. bioRxiv 2024.08.09.607358 [Preprint] (2024). https://doi.org/10.1101/2024.08.09.607358.

35. K. Atsumi, Y. Nishida, M. Ushio, H. Nishi, T. Genroku, S. Fujiki, Boosting biodiversity monitoring using smartphone-driven, rapidly accumulating community-sourced data. *eLife* **13**, RP9394 (2024).

36. J. S. Carvalho, B. Graham, H. Rebelo, G. Bocksberger, C. F. J. Meyer, S. Wich, H. S. Kuhl, A global risk assessment of primates under climate and land use change. *Glob. Chang. Biol.* **25**, 3163–3178 (2019).

37. A. Estrada, P. A. Garber, A. B. Rylands, C. Roos, E. Fernandez-Duque, A. D. Fiore, K. A. Nekaris, V. Nijman, E. W. Heymann, J. E. Lambert, F. Rovero, C. Barelli, J. M. Setchell, T. R. Gillespie, R. A. Mittermeier, L. V. Arregoitia, M. D. Guinea, S. Gouveia, R. Dobrovolski, A. Shanee, N. Shanee, S. A. Boyle, A. Fuentes, K. C. Mackinnon, K. R. Amato, A. L. S. Meyer, S. Wich, R. W. Sussman, R. L. Pan, I. Kone, B. G. Li, Impending extinction crisis of the world's primates: Why primates matter. *Sci. Adv.* **3**, e1600946 (2017).

38. A. Guisan, R. Tingley, J. B. Baumgartner, I. Naujokaitis-Lewis, P. R. Sutcliffe, A. I. T. Tulloch, T. J. Regan, L. Brotons, E. McDonald-Madden, C. Mantyka-Pringle, T. G. Martin, J. R. Rhodes, R. Maggini, S. A. Setterfield, J. Elith, M. W. Schwartz, B. A. Wintle, O. Broennimann, M. Austin, S. Ferrier, M. R. Kearney, H. P. Possingham, Y. M. Buckley, Predicting species distributions for conservation decisions. *Ecol. Lett.* **16**, 1424–1435 (2013).

39. G. Zhu, M. Papes, X. Giam, S. H. Cho, P. R. Armsworth, Are protected areas well-sited to support species in the future in a major climate refuge and corridor in the United States? *Biol. Conserv.* **255**, 108992 (2021).

40. J. E. Lambert, P. A. Garber, Evolutionary and ecological implications of primate seed dispersal. *Am. J. Primatol.* **45**, 9–28 (1998).

41. S. A. Wich, A. J. Marshall, *An Introduction to Primate Conservation* (Oxford Univ. Press, 2016).

42. R. W. Sussman, D. T. Rasmussen, P. H. Raven, Rethinking primate origins again. *Am. J. Primatol.* **75**, 95–106 (2013).

43. E. W. Heymann, Florivory, nectarivory, and pollination-A review of primate-flower interactions. *Ecotropica* **17**, 41–52 (2011).

44. C. A. Chapman, T. R. Bonnell, J. F. Gogarten, J. E. Lambert, P. A. Omeja, D. Twinomugisha, M. D. Wasserman, J. M. Rothman, Are primates ecosystem engineers? *Int. J. Primatol.* **34**, 1–14 (2013).

45. I. Kone, J. E. Lambert, J. Refisch, A. Bakayoko, Primate seed dispersal and its potential role in maintaining useful tree species in the Taï region, Côte-d'Ivoire: Implications for the conservation of forest fragments. *Trop. Conserv. Sci.* **1**, 293–305 (2008).

46. P. A. Garber, A. Estrada, S. Shanee, M. S. Svensson, L. V. Arregoitia, V. Nijman, N. Shanee, S. F. Gouveia, K. A. I. Nekaris, A. Chaudhary, J. C. Bicca-Marques, Global wildlife trade and trafficking contribute to the world's nonhuman primate conservation crisis. *Front. Conserv. Sci.* **5**, 1400613 (2024).

47. L. B. Zhang, E. I. Ameca, G. Cowlishaw, N. Pettorelli, W. Foden, G. M. Mace, Global assessment of primate vulnerability to extreme climatic events. *Nat. Clim. Chang.* **9**, 554–561 (2019).

48. E. I. Ameca y Juarez, G. M. Mace, G. Cowlishaw, W. A. Cornforth, N. Pettorelli, Assessing exposure to extreme climatic events for terrestrial mammals. *Conserv. Lett.* **6**, 145–153 (2013).

49. R. G. Pearson, J. C. Stanton, K. T. Shoemaker, M. E. Aiello-Lammens, P. J. Ersts, N. Horning, D. A. Fordham, C. J. Raxworthy, H. Y. Ryu, J. McNees, H. R. Akçakaya, Life history and spatial traits predict extinction risk due to climate change. *Nat. Clim. Chang.* **4**, 217–221 (2014).

50. P. R. Sesink Clee, E. E. Abwe, R. D. Ambahe, N. M. Anthony, R. Fotso, S. Locatelli, F. Maisels, M. W. Mitchell, B. J. Morgan, A. A. Pokempner, M. K. Gonder, Chimpanzee population structure in Cameroon and Nigeria is associated with habitat variation that may be lost under climate change. *BMC Evol. Biol.* **15**, 2 (2015).

51. W. B. Li, Y. Teng, M.-Y. Zhang, Y. Shen, J.-W. Liu, J.-W. Qi, X.-C. Wang, R.-F. Wu, J.-H. Li, P. A. Garber, M. Li, Human activity and climate change accelerate the extinction risk to non-human primates in China. *Glob. Chang. Biol.* **30**, e17114 (2023).

52. A. Schloss, T. A. Nunez, J. J. Lawler, Dispersal will limit ability of mammals to track climate change in the Western Hemisphere. *Proc. Natl. Acad. Sci. U.S.A.* **109**, 8606–8611 (2012).

53. S. F. Gouveia, J. P. Souza-Alves, L. Rattis, R. Dobrovolski, L. Jerusalinsky, R. Beltrão-Mendes, S. F. Ferrari, Climate and land use changes will degrade the configuration of the landscape for titi monkeys in eastern Brazil. *Glob. Chang. Biol.* **22**, 2003–2012 (2016).

54. Y. Teng, W. B. Li, X. C. Wang, R. S. Zhang, Y. Shen, R. F. Wu, J. W. Liu, M. Y. Zhang, C. Roos, J. H. Li, J. Li, J. W. Qi, M. Li, Integrating population genomics and environmental data to predict adaptation to climate change in post-bottleneck Tibetan macaques. *Sci. Adv.* **11**, eadw0562 (2025).

55. J. M. Tylianakis, R. K. Didham, J. Bascompte, D. A. Wardle, Global change and species interactions in terrestrial ecosystems. *Ecol. Lett.* **11**, 1351–1363 (2008).

56. Y. Malhi, J. T. Roberts, R. A. Betts, T. J. Killeen, W. Li, C. A. Nobre, Climate change, deforestation, and the fate of the Amazon. *Science* **319**, 169–172 (2008).

57. R. Wiederholt, E. Post, Tropical warming and the dynamics of endangered primates. *Biol. Lett.* **6**, 257–260 (2010).

58. W. Xu, Y. H. Wu, W.-W. Zhou, H.-M. Chen, B.-L. Zhang, J.-M. Chen, W. H. Xu, D.-Q. Rao, H. P. Zhao, F. Yan, Z. Y. Yuan, K. Jiang, J.-Q. Jin, M. Hou, D. H. Zou, L.-J. Wang, Y. Zheng, J.-T. Li, J. P. Jiang, X.-M. Zeng, Y. H. Chen, Z.-Y. Liao, C. Li, X.-Y. Li, W. Gao, K. Wang, D. R. Zhang, C. Q. Lu, T. T. Yin, Z. L. Ding, G.-G. Zhao, J. Chai, W.-G. Zhao, Y.-P. Zhang, J. J. Wiens, J. Che, Hidden hotspots of amphibian biodiversity in China. *Proc. Natl. Acad. Sci. U.S.A.* **121**, e2320674121 (2024).

59. H. Wu, L. Yu, X. L. Shen, L. Zhu, T. Hua, J. Q. Zhao, Y. Cao, Z. R. Du, T. Liu, W. C. Qi, S. J. Zheng, Q. Zhao, J. S. Tan, L. J. How, Y. X. Li, Z. F. Shu, A. Abreu, K. P. Ma, Potential of the world network of biosphere reserves to advance the kunming-montreal global biodiversity framework. *Natl. Sci. Rev.* **12**, nwaf449 (2025).

60. S. Álvarez-Carretero, A. U. Tamuri, M. Battini, F. F. Nascimento, E. Carlisle, R. J. Asher, Z. H. Yang, P. C. J. Donoghue, M. dos Reis, A species-level timeline of mammal evolution integrating phylogenomic data. *Nature* **602**, 263–267 (2022).

61. Y. Shao, L. Zhou, F. Li, L. Zhao, B.-L. Zhang, F. Shao, J.-W. Chen, C. Y. Chen, X. P. Bi, X.-L. Zhuang, H.-L. Zhu, J. Hu, Z. Y. Sun, X. Li, D. P. Wang, I. Rivas-González, S. Wang, Y.-M. Wang, W. Chen, G. Li, H. M. Lu, Y. Liu, L. F. K. Kuderna, K. K.-H. Farh, P.-F. Fan, L. Yu, M. Li, Z.-J. Liu, G. P. Tiley, A. D. Yoder, C. Roos, T. Hayakawa, T. Marques-Bonet, J. Rogers, P. D. Stenson, D. N. Cooper, M. H. Schierup, Y.-G. Yao, Y.-P. Zhang, W. Wang, X.-G. Qi, G. J. Zhang, D.-D. Wu, Phylogenomic analyses provide insights into primate evolution. *Science* **380**, 913–924 (2023).

62. S. Harrison, R. Noss, Endemism hotspots are linked to stable climatic refugia. *Ann. Bot.* **119**, 207–214 (2017).

63. S. Acevedo, B. Sandel, Phylogenetic endemism hotspots of North American birds are associated with warm temperatures and long- and short-term climate stability. *Front. Ecol. Evol.* **9**, 645396 (2021).

64. L.-M. Lu, L.-F. Mao, T. Yang, J.-F. Ye, B. Liu, H.-L. Li, M. Sun, J. T. Miller, S. Mathews, H.-H. Hu, Y.-T. Niu, D.-X. Peng, Y.-H. Chen, S. A. Smith, M. Chen, K.-L. Xiang, C.-T. Le, V.-C. Dang, A.-M. Lu, P. S. Soltis, D. E. Soltis, J.-H. Li, Z.-D. Chen, Evolutionary history of the angiosperm flora of China. *Nature* **554**, 234–238 (2018).

65. M. Esperson-Rodriguez, M. G. Tjoelker, J. Lenoir, J. B. Baumgartner, L. J. Beaumont, D. A. Nipperess, S. A. Power, B. Richard, P. D. Rymer, R. V. Gallagher, Climate change increases global risk to urban forests. *Nat. Clim. Chang.* **12**, 950–955 (2022).

66. R. Warren, J. Price, E. Graham, M. Forstenhaeusler, J. Vanderwal, The projected effect on insects, vertebrates, and plants of limiting global warming to 1.5°C rather than 2°C. *Science* **360**, 791–795 (2018).

67. G. R. Walther, E. Post, P. Convey, A. Menzel, C. Parmesan, T. J. C. Beebee, J. M. Fromentin, O. Hoegh-Guldberg, F. Bairlein, Ecological responses to recent climate change. *Nature* **416**, 389–395 (2002).

68. J. Borevitz, Utilizing genomics to understand and respond to global climate change. *Genome Biol.* **22**, 91 (2021).

69. IUCN, The IUCN red list of threatened species (2025).

70. Y. Yang, C. Li, Y.-H. Fang, G.-P. Ren, Z.-P. Huang, Y.-P. Li, L.-W. Cui, M.-R. Chu-yuan, C. A. Chapmman, C. C. Grueter, J. Zhou, W.-B. Yan, X. Liu, L.-X. Zhang, R.-A. Tang, Y.-P. Tian, F. Li, K.-G. Hang, P.-Y. Li, P. A. Garber, R.-D. Wu, W. Xiao, Effect of primate protection on threatened and endemic vertebrates, plants, ecosystem services, and future climate refugia. *Conserv. Biol.* **39**, e70019 (2025).

71. L. Yang, T. Chen, K. C. Shi, L. Zhang, N. Lwin, P.-F. Fan, Effects of climate and land-cover change on the conservation status of gibbons. *Conserv. Biol.* **37**, e14045 (2022).

72. M. Tietje, A. Antonelli, W. J. Baker, R. Govaerts, S. A. Smith, W. L. Eiserhardt, Global variation in diversification rate and species richness are unlinked in plants. *Proc. Natl. Acad. Sci. U.S.A.* **119**, e2120662119 (2022).

73. J. H. Arbour, S. E. Santana, A major shift in diversification rate helps explain macroevolutionary patterns in primate species diversity. *Evolution* **71**, 1600–1613 (2017).

74. A. C. A. Silva, O. Maliet, L. Aristide, D. Nogués-Bravo, N. Upham, W. Jetz, H. Morlon, Negative global-scale association between genetic diversity and speciation rates in mammals. *Nat. Commun.* **16**, 1796 (2025).

75. L. R. Godfrey, K. E. Samonds, J. W. Baldwin, M. R. Sutherland, J. M. Kamilar, K. L. Allfisher, Mid-Cenozoic climate change, extinction, and faunal turnover in Madagascar, and their bearing on the evolution of lemurs. *BMC Evol. Biol.* **20**, 97 (2020).

76. A. Antonelli, W. D. Kissling, S. G. A. Flantua, M. A. Bermúdez, A. Mulch, A. N. Muellner-Riehl, H. Kreft, H. P. Linder, C. Badgley, J. Fjeldså, S. A. Fritz, C. Rahbek, F. Herman, H. Hooghiemstra, C. Hoorn, Geological and climatic influences on mountain biodiversity. *Nat. Geosci.* **11**, 718–725 (2018).

77. J. L. Alfaro, The monkeying of the Americas: Primate biogeography in the neotropics. *Ann. Rev. Anthropol.* **46**, 263–282 (2017).

78. Y. B. Hu, H. Z. Fan, Y. H. Chen, J. Cheng, X. J. Zhan, H. Wu, B. W. Zhang, M. Wang, W. Y. Zhang, L. Yang, X. Hou, X. Shen, T. Pan, W. Wu, J. Li, H. H. Hu, F. W. Wei, Spatial patterns and genetic and phylogenetic diversity of wildlife in China. *Sci. Adv.* **7**, abd5725 (2021).

79. N. E. Heller, E. S. Zavaleta, Biodiversity management in the face of climate change: A review of 22 years of recommendations. *Biol. Conserv.* **142**, 14–32 (2009).

80. J. Gao, Y. H. Liu, Climate stability is more important than water-energy variables in shaping the elevational variation in species richness. *Ecol. Evol.* **8**, 6872–6879 (2018).

81. K. P. Bhatta, B. A. Robson, M. K. Suwal, O. R. Vetaas, A pan-himalayan test of predictions on plant species richness based on primary production and water-energy dynamics. *Front. Biogeogr.* **13**, e49459 (2021).

82. A. Ozgul, C. Fichtel, M. Paniw, P. M. Kappeler, Destabilizing effect of climate change on the persistence of a short-lived primate. *Proc. Natl. Acad. Sci. U.S.A.* **120**, e2214244120 (2023).

83. L. R. Cai, H. Kreft, A. Taylor, J. Schrader, W. Dawson, F. Essl, M. Kleunen, J. Pergl, P. Pyšek, M. Winter, P. Weigelt, Climatic stability and geological history shape global centers of neo- and paleoendemism in seed plants. *Proc. Natl. Acad. Sci. U.S.A.* **120**, e2300981120 (2023).

84. L. Donaldson, J. J. Bennie, R. J. Wilson, I. M. Maclean, Designing effective protected area networks for multiple species. *Biol. Conserv.* **258**, 109125 (2021).

85. J. Haight, E. Hammill, Protected areas as potential refugia for biodiversity under climatic change. *Biol. Conserv.* **241**, 108258 (2020).

86. T. Chen, P. A. Garber, L. Zhang, L. Yang, P. F. Fan, The pattern and drivers of taxonomic bias in global primate research. *Glob. Ecol. Conserv.* **46**, e02599 (2023).

87. V. Popov, P. Shah, R. K. Runting, J. R. Rhodes, Managing risk and uncertainty in systematic conservation planning with insufficient information. *Methods Ecol. Evol.* **13**, 230–242 (2021).

88. J. L. Martin, V. Maris, D. S. Simberloff, The need to respect nature and its limits challenges society and conservation science. *Proc. Natl. Acad. Sci. U.S.A.* **113**, 6105–6112 (2016).

89. B. Czech, P. R. Krausman, P. K. Devers, Economic associations among causes of species endangerment in the United States. *Bioscience* **50**, 593–601 (2000).

90. B. G. Li, M. Li, J. H. Li, P. F. Fan, Q. Y. Ni, J. Q. Lu, X. M. Zhou, Y. C. Long, Z. G. Jiang, P. Zhang, Z. P. Huang, C. M. Huang, X. L. Jiang, R. L. Pan, S. Gouveia, R. Dobeovolski, C. C. Grueter, C. Oxnard, C. Groves, A. Estrada, P. A. Garber, The primate extinction crisis in China: Immediate challenges and a way forward. *Biodivers. Conserv.* **27**, 3301–3327 (2018).

91. X. M. Zhao, P. A. Garber, M. Li, Alleviating human poverty: A successful model promoting wildlife conservation in China. *Conserv. Sci. Pract.* **3**, e511 (2021).

92. F. Affinito, J. M. Williams, J. E. Campbell, M. C. Londono, A. Gonzalez, Progress in developing and operationalizing the monitoring framework of the global biodiversity framework. *Nat. Ecol. Evol.* **8**, 2163–2171 (2024).

93. K. Katoh, K. Misawa, K. Kuma, T. Miyata, MAFFT: A novel method for rapid multiple sequence alignment based on fast Fourier transform. *Nucleic Acids Res.* **30**, 3059–3066 (2002).

94. G. Vaidya, D. J. Lohman, R. Meier, SequenceMatrix: Concatenation software for the fast assembly of multi-gene datasets with character set and codon information. *Cladistics* **27**, 171–180 (2011).

95. A. Stamatakis, RAxML version 8: A tool for phylogenetic analysis and post-analysis of large phylogenies. *Bioinformatics* **30**, 1312–1313 (2014).

96. S. A. Smith, B. C. O'Meara, treePL: Divergence time estimation using penalized likelihood for large phylogenies. *Bioinformatics* **28**, 2689–2690 (2012).

97. S. B. Hedges, J. Marin, M. Suleski, M. Paymer, S. Kumar, Tree of life reveals clock-like speciation and diversification. *Mol. Biol. Evol.* **32**, 835–845 (2015).

98. D. L. Rabosky, Automatic detection of key innovations, rate shifts, and diversity-dependence on phylogenetic trees. *PLOS ONE* **9**, e89543 (2014).

99. J. J. Shi, D. L. Rabosky, Speciation dynamics during the global radiation of extant bats. *Evolution* **69**, 1528–1545 (2015).

100. D. L. Rabosky, J. Chang, P. O. Title, P. F. Cowman, L. Sallan, M. Friedman, K. Kaschner, C. Garilao, T. J. Near, M. Coll, M. E. Alfaro, An inverse latitudinal gradient in speciation rate for marine fishes. *Nature* **559**, 392–395 (2018).

101. D. L. Rabosky, M. Grundler, C. Anderson, P. Title, J. J. Shi, J. W. Brown, H. Huang, J. G. Larson, BAMMtools: An R package for the analysis of evolutionary dynamics on phylogenetic trees. *Methods Ecol. Evol.* **5**, 701–707 (2014).

102. A. Voldoire, E. Sanchez-Gomez, D. Salas y Mélia, B. Decharme, C. Cassou, S. Sénési, S. Valcke, I. Beau, A. Alias, M. Chevallier, M. Déqué, J. Deshayes, H. Douville, E. Fernandez, G. Madec, E. Maisonnave, M.-P. Moine, S. Planton, D. Saint-Martin, S. Szopa, S. Tyteca, R. Alkama, S. Belamari, A. Braun, L. Coquart, F. Chauvin, The CNRM-CM5.1 global climate model: Description and basic evalution. *Climate Dynam.* **40**, 2091–2121 (2013).

103. K. Zhang, L. Yao, J. Meng, J. Tao, Maxent modeling for predicting the potential geographical distribution of two peony species under climate change. *Sci. Total Environ.* **634**, 1326–1334 (2018).

104. J. Elith, J. R. Leathwick, T. Hastie, A working guide to boosted regression trees. *J. Anim. Ecol.* **77**, 802–813 (2008).

105. U. Roll, A. Feldman, M. Novosolov, A. Allison, A. M. Bauer, R. Bernard, M. Böhm, F. Castro-Herrera, L. Chirio, B. Collen, G. R. Colli, R. Dabool, I. Das, T. M. Doan, L. L. Grismer, M. Hoogmoed, Y. Itescu, F. Kraus, M. L. Breton, A. Lewin, M. Martins, E. Maza, D. Meirte, Z. T. Nagy, C. Nogueira, O. S. G. Pauwels, D. Pincheira-Donoso, G. D. Powney, R. Sindaco, O. J. S. Tallowin, O. Torres-Carvajal, J. F. Trape, E. Vidan, P. Uetz, P. Wagner, Y. Wang, C. D. L. Orme, R. Grenyer, S. Meiri, The global distribution of tetrapods reveals a need for targeted reptile conservation. *Nat. Ecol. Evol.* **1**, 1677–1682 (2017).

106. B. Saladin, L. Pellissier, C. H. Graham, M. P. Nobis, N. Salamin, N. E. Zimmermann, Rapid climate change results in long-lasting spatial homogenization of phylogenetic diversity. *Nat. Commun.* **11**, 4663 (2020).

107. T. Y. Zhang, C. X. Cheng, X. D. Wu, Mapping the spatial heterogeneity of global land use and land cover from 2020 to 2100 at a 1 km resolution. *Sci. Data* **10**, 748 (2023).

108. S. Liang, J. Cheng, K. Jia, B. Jiang, Q. Liu, Z. Q. Xiao, Y. J. Yao, W. P. Yuan, X. T. Zhang, X. Zhao, J. Zhou, The global land surface satellite (GLASS) product suite. *Bull. Am. Meteorol. Soc.* **102**, E323–E337 (2021).

109. N. J. Gotelli, D. J. McCabe, Species co-occurrence: A meta-analysis of JM Diamond's assembly rules model. *Ecology* **83**, 2091–2096 (2002).

110. N. J. Gotelli, G. L. Entsminger, Swap algorithms in null model analysis. *Ecology* **84**, 532–535 (2003).

111. R. Molina-Venegas, G. Ottaviani, G. Campetella, R. Canullo, S. Chelli, Biogeographic deconstruction of phylogenetic and functional diversity provides insights into the formation of regional assemblages. *Ecography* **2022**, e06140 (2022).

112. S. A. Fritz, C. Rahbek, Global patterns of amphibian phylogenetic diversity. *J. Biogeogr.* **39**, 1373–1382 (2012).

113. R. Gumbs, C. L. Gray, M. Bohm, M. Hoffmann, R. Grenyer, W. Jetz, S. Meiri, U. Roll, N. R. Owen, J. Rosindell, Global priorities for conservation of reptilian phylogenetic diversity in the face of human impacts. *Nat. Commun.* **11**, 2616 (2020).

114.. Borko, F. Altermatt, M. Zagmajster, C. Fišer, A hotspot of groundwater amphipod diversity on a crossroad of evolutionary radiations. *Divers. Distrib.* **28**, 2765–2777 (2022).

115. W. Dawson, D. Moser, M. van Kleunen, H. Kreft, J. Pergl, P. Pyšek, P. Weigelt, M. Winter, B. Lenzner, T. M. Blackburn, E. E. Dyer, P. Cassey, S. L. Scrivens, E. P. Economo, B. Guénard, C. Capinha, H. Seebens, P. García-Díaz, W. Nentwig, E. García-Berthou, C. Casal,

N. E. Mandrak, P. Fuller, C. Meyer, F. Essl, Global hotspots and correlates of alien species richness across taxonomic groups. *Nat. Ecol. Evol.* **1**, 186 (2017).

116. T. Oberdorff, M. S. Dias, C. Jezequel, J. S. Albert, C. C. Arantes, R. Bigorne, F. M. Carvajal-Valleros, A. D. Wever, R. G. Frederico, M. Hidalgo, B. Hugueny, F. Leprieur, M. Maldonado, J. Maldonado-Ocampo, K. Martens, H. Ortega, J. Sarmiento, P. A. Tedesco, G. Torrente-Vilara, K. O. Winemiller, J. Zuanon, Unexpected fish diversity gradients in the Amazon basin. *Sci. Adv.* **5**, eaav8681 (2019).

117. R. R. Bivand, Packages for analyzing spatial data: A comparative case study with areal data. *Geogr. Anal.* **54**, 488–518 (2022).

118. H. D. Kort, J. G. Prunier, S. Ducatez, O. Honnay, M. Baguette, V. M. Stevens, S. Blanchet, Life history, climate and biogeography interactively affect worldwide genetic diversity of plant and animal populations. *Nat. Commun.* **12**, 516 (2021).

119. J. Lai, Y. Zou, S. Zhang, X. Zhang, L. Mao, glmm.hp: An R package for computing individual effect of predictors in generalized linear mixed models. *J. Plant Ecol.* **15**, 1302–1307 (2022).

120. B. H. Daru, J. Rodriguez, Mass production of unvouchered records fails to represent global biodiversity patterns. *Nat. Ecol. Evol.* **7**, 816–831 (2023).

121. G. Murali, R. Gumbs, S. Meiri, U. Roll, Global determinants and conservation of evolutionary and geographic rarity in land vertebrates. *Sci. Adv.* **7**, eabe5582 (2021).

122. A. S. L. Rodrigues, S. J. Andelman, M. I. Bakarr, L. Boitani, T. M. Brooks, R. M. Cowling, L. D. C. Fishpool, G. A. B. Fonseca, K. J. Gaston, M. Hoffmann, J. S. Long, P. A. Marquet, J. D. Pilgrim, R. L. Pressey, J. Schipper, W. Sechrest, S. N. Stuart, L. G. Underhill, R. W. Waller, M. E. J. Watts, X. Yan, Effectiveness of the global protected area network in representing species diversity. *Nature* **428**, 640–643 (2004).

123. W. H. Xu, Y. Xiao, J. J. Zhang, W. Yang, L. Zhang, V. Hull, Z. Wang, H. Zheng, J. G. Liu, S. Polasky, L. Jiang, Y. Xiao, X. W. Shi, E. Rao, F. Lu, X. K. Wang, G. C. Daily, Z. Y. Ouyang,

Strengthening protected areas for biodiversity and ecosystem services in China. *Proc. Natl. Acad. Sci. U.S.A.* **114**, 1601–1606 (2017).

124. J. H. Martins, A. S. Camanho, M. B. Gaspar, A review of the application of driving forces-pressure-state-impact-response framework to fisheries management. *Ocean Coast. Manag.* **69**, 273–281 (2012).

125. Y. Kitazoe, H. Kishino, P. J. Waddell, N. Nakajima, T. Okabayashi, T. Watabe, Y. Okuhara, Robust time estimation reconciles views of the antiquity of placental mammals. *PLOS ONE* **2**, e384 (2007).

126. S. Y. Wu, W. Y. Wu, F. C. Zhang, J. Ye, X. J. Ni, J. M. Sun, S. V. Edwards, J. Meng, C. L. Organ, Molecular and paleontological evidence for a post-Cretaceous origin of rodents. *PLOS ONE* **7**, e46445 (2012).

127. G. V. Glazko, M. Nei, Estimation of divergence times for major lineages of primate species. *Mol. Biol. Evol.* **20**, 424–434 (2003).

128. W. J. Bailey, D. H. Fitch, D. A. Tagle, J. Czelusniak, J. L. Slightom, M. Goodman, Molecular evolution of the psi eta-globin gene locus: Gibbon phylogeny and the hominoid slowdown. *Mol. Biol. Evol.* **8**, 155–184 (1991).

129. C. Poux, E. J. P. Douzery, Primate phylogeny, evolutionary rate variations, and divergence times: A contribution from the nuclear gene IRBP. *Am. J. Phys. Anthropol.* **124**, 01–16 (2004).

130. P. L. Babb, E. Fernandez-Duque, C. A. Baiduc, P. Gageux, S. Evans, T. G. Schurr, mtDNA diversity in Azara's owl monkeys (Aotus azarai azarai) of the Argentinean Chaco. *Am. J. Phys. Anthropol.* **146**, 209–224 (2011).

131. C. Poux, P. Chevret, D. Huchon, W. W. de Jong, E. J. P. Douzery, Arrival and diversification of caviomorph rodents and platyrrhine primates in South America. *Syst. Biol.* **55**, 228–244 (2006).

132. P. H. Fabre, A. Rodrigues, E. J. P. Douzery, Patterns of macroevolution among primates inferred from a supermatrix of mitochondrial and nuclear DNA. *Mol. Phylogenet. Evol.* **53**, 808–825 (2009).

133. C. G. Schrago, On the time scale of New World primate diversification. *Am. J. Phys. Anthropol.* **132**, 344–354 (2007).

134. M. C. Janiak, F. E. Silva, R. M. D. Beck, D. Vries, L. F. K. Kuderna, N. S. Torosin, A. D. Melin, T. Marques-Bonet, I. B. Goodhead, M. Messias, M. N. F. Silva, I. Sampaio, I. P. Farias, R. Rossi, F. R. Melo, J. Valsecchi, T. Hrbek, J. P. Boubli, Two hundred and five newly assembled mitogenomes provide mixed evidence for rivers as drivers of speciation for Amazonian primates. *Mol. Ecol.* **31**, 3888–3902 (2022).

135. C. G. Schrago, B. Mello, A. E. R. Soares, Combining fossil and molecular data to date the diversification of New World Primate. *J. Evol. Biol.* **26**, 2438–2446 (2013).

136. M. Goodman, C. A. Porter, J. Czelusniak, S. L. Page, H. S. Schneider, J. Shoshani, G. Gunnell, C. P. Groves, Toward a phylogenetic classification of Primates based on DNA evidence complemented by fossil evidence. *Mol. Phylogenet. Evol.* **9**, 585–598 (1998).

137. R. W. Meredith, J. E. Janečka, J. Gatesy, O. A. Ryder, C. A. Fisher, E. C. Teeling, A. Goodbla, E. Eizirik, T. L. L. Simao, T. Stadler, D. L. Rabosky, R. L. Honeycutt, J. J. Flynn, C. M. Ingram, C. Steiner, T. L. Williams, T. J. Robinson, A. Burk-Herrick, M. Westerman, N. A. Ayoub, M. S. Springer, W. J. Murphy, Impacts of the cretaceous terrestrial revolution and KPg extinction on mammal diversification. *Science* **334**, 521–524 (2011).

138. K. L. Chiou, L. Pozzi, J. W. L. Alfaro, A. D. Fiore, Pleistocene diversification of living squirrel monkeys (*Saimiri* spp.) inferred from complete mitochondrial genome sequences. *Mol. Phylogenet. Evol.* **59**, 736–745 (2011).

139. J. A. Hodgson, K. N. Sterner, L. J. Matthews, A. S. Burrell, R. A. Jani, R. L. Raaum, C. B. Stewart, T. R. Disotell, Successive radiations, not stasis, in the South American primate fauna. *Proc. Natl. Acad. Sci. U.S.A.* **106**, 5534–5539 (2009).

140. K. Mendez-Camacho, O. Leon-Alvarado, D. R. Miranda-Esquivel, Biogeographic evidence supports the Old Amazon hypothesis for the formation of the Amazon fluvial system. *PeerJ* **9**, e12533 (2021).

141. P. Perelman, W. E. Johnson, C. Roos, H. N. Seuánez, J. E. Horvath, M. A. M. Moreira, B. Kessing, J. Pontius, M. Roelke, Y. Rumpler, M. P. C. Schneider, A. Silva, S. J. O'Brien, J. Pecon-Slattery, A molecular phylogeny of living primates. *PLOS Genet.* **7**, e1001342 (2011).

142. F. Casado, C. R. Bonvicino, C. Nagle, B. Comas, T. D. Manzur, M. M. Lahoz, H. N. Seuanez, Mitochondrial divergence between 2 populations of the hooded capuchin, *Cebus* (*Sapajus*) cay (Platyrrhini, primates). *J. Hered.* **101**, 261–269 (2010).

143. H. Israfil, S. M. Zehr, A. R. Mootnick, M. Ruvolo, M. E. Striper, Unresolved molecular phylogenies of gibbons and siamangs (family: Hylobatidae) based on mitochondrial, Y-linked, and X-linked loci indicate a rapid Miocene radiation or sudden vicariance event. *Mol. Phylogenet. Evol.* **58**, 447–455 (2011).

144. M. S. Springer, R. W. Meredith, J. Gatesy, C. A. Emerling, J. Park, D. L. Rabosky, T. Stadler, C. Steiner, O. A. Ryder, J. E. Janečka, C. A. Fisher, W. J. Murphy, Macroevolutionary dynamics and historical biogeography of primate diversification inferred from a species supermatrix. *PLOS ONE* **7**, e49521 (2012).

145. S. Easteal, G. Herbert, Molecular evidence from the nuclear genome for the time frame of human evolution. *J. Mol. Evol.* **44**, S121–S132 (1997).

146. S. Kumar, S. B. Hedges, A molecular timescale for vertebrate evolution. *Nature* **392**, 917–920 (1998).

147. M. Hasegawa, H. Kishino, T. Yano, Dating of the human-ape splitting by a molecular clock of mitochondrial DNA. *J. Mol. Evol.* **22**, 160–174 (1985).

148. P. L. Babb, E. Fernandez-Duque, T. G. Schurr, AVPR1A sequence variation in monogamous owl monkeys (*Aotus azarai*) and its implications for the evolution of platyrrhine social behavior. *J. Mol. Evol.* **71**, 279–297 (2010).

149. C. Roos, D. Zinner, L. Kubatko, C. Schwarz, M. Y. Yang, D. Meyer, S. D. Nash, J. C. Xing, M. A. Batzer, M. Brameier, F. H. Leendertz, T. Ziegler, D. Perwitasari-Farajallah, T. Nadler, L. Walter, M. Osterholz, Nuclear versus mitochondrial DNA: Evidence for hybridization in colobine monkeys. *BMC Evol. Biol.* **24**, 17 (2011).

150. A. J. Tosi, K. M. Detwiler, T. R. Disotell, X-chromosomal window into the evolutionary history of the guenons (primates: Cercopithecini). *Mol. Phylogenet. Evol.* **36**, 58–66 (2005).

151. W. M. Switzer, M. Salemi, V. Shanmugam, F. Gao, M. E. Cong, C. Kuiken, V. Bhullar, B. E. Beer, D. Vallet, A. Gautier-Hion, T. Tooze, F. Villinger, E. C. Holmes, W. Heneine, Ancient co-speciation of simian foamy viruses and primates. *Nature* **434**, 376–380 (2005).

152. R. D. Wilkinson, M. E. Striper, C. Soligo, R. D. Martin, Z. H. Yang, S. Tavare, Dating primate divergences through an integrated analysis of palaeontological and molecular data. *Syst. Biol.* **60**, 16–31 (2011).

153. N. Ting, Mitochondrial relationships and divergence dates of the African colobines: Evidence of Miocene origins for the living colobus monkeys. *J. Hum. Evol.* **55**, 312–325 (2008).

154. K. He, N. Q. Hu, J. D. Orkin, D. T. Nyein, C. Ma, W. Xiao, P. F. Fan, X. L. Jiang, Molecular phylogeny and divergence time of Trachypithecus: With implications for the taxonomy of *T. phayrei*. *Zool. Res.* **33**, E104–E110 (2012).

155. V. N. Thinh, A. R. Mootnick, T. Geissmann, M. Li, T. Ziegler, M. Agil, P. Moisson, T. Nadler, L. Walter, C. Roos, Mitochondrial evidence for multiple radiations in the evolutionary history of small apes. *BMC Evol. Biol.* **12**, 74 (2010).

156. M. D. Reis, G. F. Gunnell, J. Barba-Montoya, A. Willkins, Z. H. Yang, A. D. Yoder, Using Phylogenomic data to explore the effects of relaxed clocks and calibration strategies on divergence time estimation: Primates as a test case. *Syst. Biol.* **67**, 594–615 (2018).

157. R. Liedigk, M. Y. Yang, N. G. Jablonski, F. Momberg, T. Geissmann, N. Lwin, T. H. Hla, Z. J. Liu, B. Wong, M. Li, Y. C. Long, Y. P. Zhang, N. Tilo, D. Zinner, C. Roos, Evolutionary history of the odd-nosed monkeys and the phylogenetic position of the newly described Myanmar snub-nosed monkey *Rhinopithecus strykeri*. *PLOS ONE* **7**, e37418 (2012).

158. M. Y. Yang, Y. Q. Yang, D. Y. Cui, G. Fickenscher, D. Zinner, C. Roos, M. Brameier, Population genetic structure of Guizhou snub-nosed monkeys (*Rhinopithecus brelichi*) as inferred from mitochondrial control region sequences, and comparison with *R. roxellana* and *R. bieti*. *Am. J. Phys. Anthropol.* **147**, 1–10 (2012).

159. L. Pozzi, T. R. Disotell, J. C. Masters, A multilocus phylogeny reveals deep lineages within African galagids (primates: Galagidae). *BMC Evol. Biol.* **2**, 72 (2014).

160. L. Pozzi, K. A.-I. Nekaris, A. Perkin, S. K. Bearder, E. R. Pimley, H. Schulze, U. Streicher, T. Nadler, A. Kitchener, H. Zischler, D. Zinner, C. Roos, Remarkable ancient divergences amongst neglected lorisiform primates. *Zool. J. Linn. Soc.* **175**, 661–674 (2015).
